# Co-templating of polyoxoniobates and silicate/germanate trimer-rings in crystals and inorganic gels

**DOI:** 10.1039/d6sc02933g

**Published:** 2026-05-19

**Authors:** Makenzie T. Nord, Andrew P. Porter, Detcho J. N. Aboa, Robert S. Minning, Karlie Bach, Emily Hiatt, Wesley T. Surta, Lev N. Zakharov, Aaron J. Rossini, May Nyman

**Affiliations:** a Department of Chemistry, Oregon State University Corvallis OR 97331 USA may.nyman@oregonstate.edu; b Department of Chemistry, Iowa State University Ames IA 50011 USA; c Ames National Laboratory, Division of Materials Science and Engineering Ames Iowa 50011 USA; d Phosio Corporation Corvallis OR 97330 USA; e Department of Mechanical, Industrial, and Manufacturing Engineering, Oregon State University Corvallis OR 97331 USA

## Abstract

Polyoxometalate (POM) supramolecular gels are a growing family of materials, both for understanding fundamental self-assembly and fabricating flexible monolithic materials that retain the function of metal oxides. Here, we exploited the pH-dependent speciation of polyoxoniobates (PONbs), targeting the formation of PONb-containing supramolecular gels that contain other low molecular weight components (silicate, germanate, phosphate, and carbonate). The introduction of gaseous CO_2_ into aqueous hexaniobate solutions resulted in the formation of both new crystalline phases and highly transparent gels. The crystalline phases, formulated Cs_24_[Nb_7_O_22_(NbO(CO_3_)_2_)_9_(Si_3_O_9_)]·19.6H_2_O and Cs_21_Na_3_[Nb_7_O_22_(NbO(CO_3_)_2_)_9_(Ge_3_O_9_)]·33.6H_2_O are templated by a rare planar [X_3_O_9_]^6−^ (X = Si, Ge) ring. Crystalline phases were not obtained with phosphate; instead, the gels contain a mixture of phosphate-centred PONbs and network-forming phosphate. POM speciation within the gels, physical properties, and assembly mechanisms were benchmarked by solution and solid-state nuclear magnetic resonance (NMR) spectroscopy, vibrational spectroscopies, small-angle X-ray scattering (SAXS), and thermogravimetry-mass spectroscopy. Optical analysis and dielectric behavior of the gels confirmed that they are highly transparent ionic and electronic conductors. The alkali and hydroxide concentration controls the formation of crystalline materials or supramolecular gels while maintaining the same network building blocks, providing a rare opportunity to describe the molecular-level structure of inorganic amorphous materials.

## Abstract 2

Polyoxometalate (POM) supramolecular gels are a growing family of materials, both for understanding fundamental self-assembly and fabricating flexible monolithic materials that retain the function of metal oxides.

## Graphical abstract

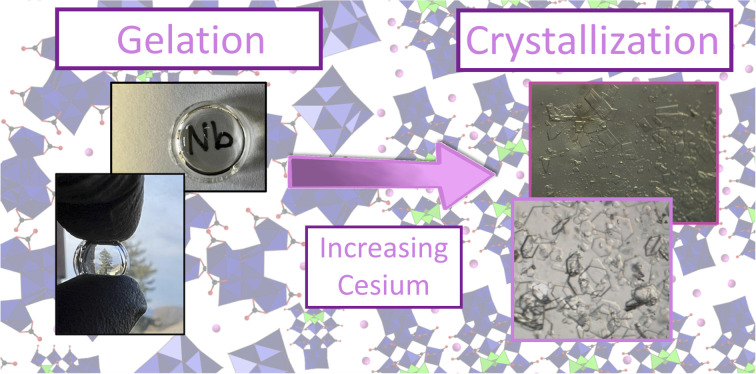

## Introduction

Supramolecular gels are solid-like networks composed of molecules associated by non-covalent interactions, including hydrogen bonding, electrostatics, π–π stacking, and hydrophobicity.^1–3^ While gels are difficult to define, a standard qualitative method involves the ‘inversion test’, where a lack of flow signifies gelation.^2^ Supramolecular gels self-assemble primarily through molecular recognition, with higher order assembly into secondary structures including fibres, micelles, ribbons, and sheets. The secondary structures form tertiary networks that trap the solvent.^1,3–5^ The multicomponent nature of supramolecular gels, giving rise to flexibility and responsiveness to external stimuli, is exploited for drug delivery, electronics, and templating.^1–3,6,7^ However, the lack of long-range order challenges structural characterization, and therefore, understanding of form–function relationships is lacking.^1,6^

More recently, supramolecular metallogels constructed from polyoxometalates (POMs)^8–13^ have brought forth applications in sensing,^14–16^ electronics,^17–19^ catalysis,^20,21^ and biomedicine.^22,23^ POMs are defined as inorganic molecular oxoclusters built of group 5 and group 6 transition metals in their highest d^0^ oxidation state. The d^0^-metyl species, V=O^3+^, Nb=O^3+^, Ta=O^3+^, Mo=O^4+^, or W=O^4+^, cap and stabilize polynuclear oxoclusters without organic ligands that hinder reactivity. The ability of POMs to interact through protonation/H-bonding and counterion association makes them ideal building blocks in supramolecular systems.^11,24^

Despite the yl-oxo capping groups, many POM-gels additionally rely on modification with an organic moiety,^8,10^ require organic solvent,^25^ or consist of POMs embedded within a polymer network.^12,24,26^ Purely inorganic gels^13,27,28^ are limited, and supramolecular gels of polyoxoniobates (PONbs) are virtually unknown. Although PONb chemistry is now well-established, its structure–function relationships in extended or soft materials are emerging.^29,30^

We are currently developing room temperature solution routes for supramolecular assembly of PONbs into transparent gels and glasses. PONbs (and Ta-POMs), unlike W, Mo, and V POMs, are redox inert and stable in alkaline conditions,^31^ offering differentiating properties including pH-compatibility with inorganic glass network formers such as silicates, borates, and phosphates.

The solubility of PONbs is primarily controlled by their countercations,^31–36^ and our early studies exploited tetramethylammonium (TMA), which enabled dissolution of up to 3 M niobium at room temperature in neutral to slightly alkaline conditions. In those studies, gelation proceeded by conversion of [Nb_10_O_28_]^6−^ (Nb_10_) to [H_*x*_Nb_24_O_72_]^(24−*x*)–^ (Nb_24_) building blocks.^37^ The gelation behaviour is ideal for deposition of conformal, smooth thin films in mild conditions.^32,37,38^ Molecular-level detail further described that the lability of Nb_24_ in water leads to network formation. Furthermore, ESI-MS detected cationic Nb_3_ ^37^ charge-balancing Nb_24_ and its Nb_7_ subunits, leading to ion-association and polymerization. However, creating niobia networks using Nb_10_ is not ideal because Nb_10_ has only ever been isolated in high yields as a TMA salt. The organic TMA must subsequently be eliminated from inorganic networks for many targeted applications by either high-temperature combustion^39^ or ion exchange,^40^ where the former leads to crystallization and the latter disrupts transparent, defect-free morphologies.

**Fig. 1:**
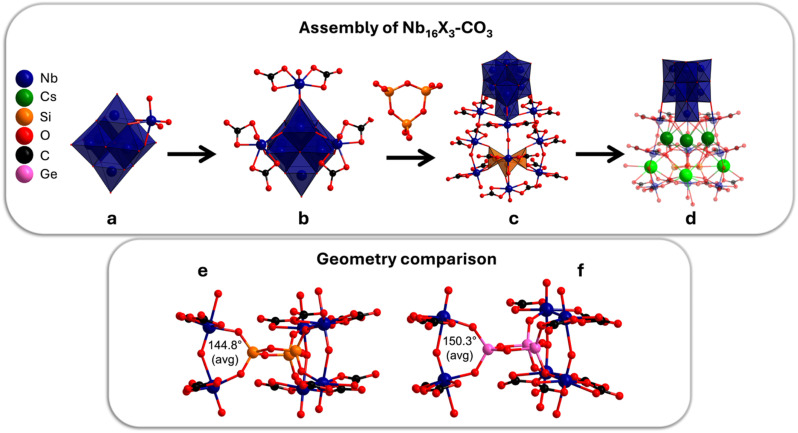
Overview of Nb_16_X_3_–CO_3_ (X = Si or Ge) structure. (a) The Nb_7_ unit consists of the Nb_6_-Lindqvist ion with one additional NbO_6_-octahedron that shares three edges with Nb_6_. (b) Nb_10_–CO_3_: three NbO(CO_3_)_2_ cap the Nb_7_-unit. (c) Two additional Nb(CO_3_)_2_O_2_ monomers extend each of the three aforementioned NbO(CO_3_)_2_ caps, forming the ‘tentacles’ that encapsulate a cyclic X_3_O_9_ trimer. (d) Cs^+^ countercations associated with the tentacles of Nb_16_X_3_–CO_3_. Comparison of the Nb–O–Nb bond angles resulting from accommodation of the different-sized X_3_O_9_ trimers in Nb_16_Si_3_–CO_3_ (e) and Nb_16_Ge_3_–CO_3_ (f).

**Fig. 2:**
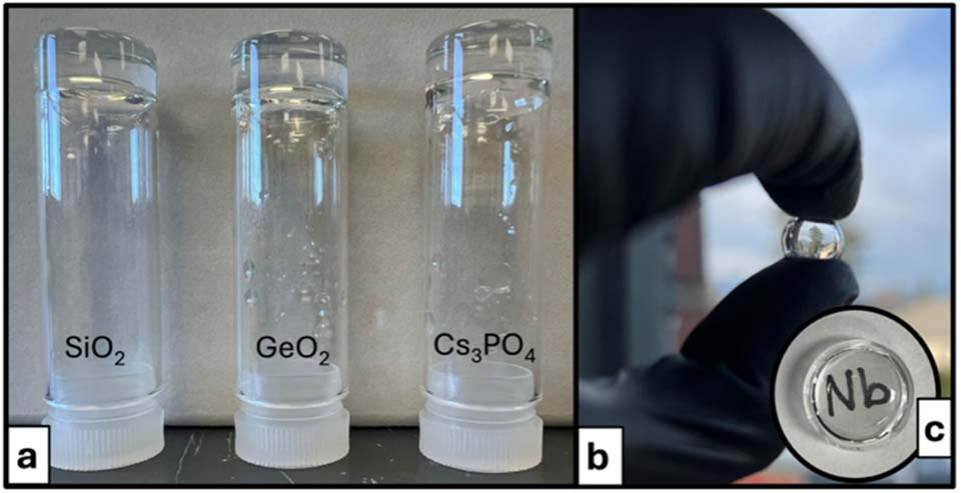
(a) The gels formed from Cs–Nb_6_ with SiO_2_, GeO_2_, or Cs_3_PO_4_ and CO_2_ infusion. (b) and (c) highlight gel transparency and retention of monolithic shape when removed from their container.

In recent studies, we demonstrated that infusion of aqueous Nb_6_ ([Nb_6_O_19_]^8−^) solutions with CO_2_ lowers the pH and converts Nb_6_ to Nb_24_ (TMA counter cations) or Nb–carbonate POMs (alkali counter cations),^41^ completing bidirectional, pH-driven, and countercation-dependent PONb speciation change between pH 7 and 14. In this current work, we utilize Nb_6_ and inorganic oxoanions (silicate, germanate, phosphate, and carbonate) as building blocks to create all-inorganic, PONb supramolecular gels, exploring how local coordination chemistry drives assembly. Carbon dioxide infusion into Cs(Na)–Nb_6_–GeO_2_, Cs–Nb_6_–SiO_2_, and Cs–Nb_6_–P_2_O_5_ solutions produced either novel PONbs (crystallized) at higher alkali and hydroxide concentration (Fig. 1) or transparent gels at lower alkali and hydroxide concentration (Fig. 2). In contrast to previously reported all-inorganic supramolecular gels that are translucent (the exception being recent Mo_7_–Fe Hofmeister gels^13^),^27,28^ our gels are highly transparent, enabling analysis of optical properties. The PONbs, respectively formulated Cs_24_[Nb_7_O_22_(NbO(CO_3_)_2_)_9_(Si_3_O_9_)]·19.6H_2_O and Cs_21_Na_3_[Nb_7_O_22_(NbO(CO_3_)_2_)_9_(Ge_3_O_9_)]·33.6H_2_O (Nb_16_Si_3_–CO_3_ and Nb_16_Ge_3_–CO_3_ for brevity), are templated by a rare planar [X_3_O_9_]^6−^ (X = Si, Ge) ring observed prior only in minerals^42^ and other high temperature/pressure synthetic phases.^43–46^ In addition to single-crystal X-ray diffraction, solution characterization of the PONbs demonstrates assembly pathways. Solutions, crystals, and gels were characterized by nuclear magnetic resonance (NMR) spectroscopy (^133^Cs, ^29^Si, ^31^P, solution and solid-state), small-angle X-ray scattering (SAXS), Raman and Fourier-transform infrared (FTIR) spectroscopies, thermogravimetry-differential scanning calorimetry-mass spectrometry (TGA-DSC-MS), CHN combustion analysis and scanning electron microscopy/energy-dispersive X-ray spectroscopy (SEM/EDS). Using the PONbs to benchmark the structural characterization of the gels reveals amorphous networks composed of POM building blocks. Optical measurements demonstrated high optical clarity, while impedance spectroscopy benchmarks conductivity in the gel matrices.

## Experimental

Details of synthesis and characterization are included in the SI and summarized here. Cs–Nb_6_ was synthesized as previously reported.^47^ Cs_4_SiO_4_ or Na_4_GeO_4_ solutions were made by dissolving SiO_2_ or GeO_2_ in CsOH (5.7 M) or NaOH (4 M) in a ratio of four alkali to one Si or Ge. For the crystalline systems, Cs_4_SiO_4_ or Na_4_GeO_4_ (Nb_16_Si_3_–CO_3_ and Nb_16_Ge_3_–CO_3_, respectively, Fig. 1) was added to an aqueous solution of Cs–Nb_6_ (1 M) and placed in a desiccator with dry ice overnight. Nb_16_Si_3_–CO_3_ and Nb_16_Ge_3_–CO_3_ crystals are observed within 24 hours. For the gels (Fig. 2), SiO_2_ (35 mg), GeO_2_ (12 mg), or Cs_3_PO_4_ (1.4 M, 125 µL) was added to a warm (60 °C) Cs–Nb_6_ solution (1 M) and stirred for ∼20 minutes. This solution was then placed in a desiccator with dry ice overnight. The gels remain viscous for approximately 18 hours and fully solidify (per the inversion test) within 24 to 48 hours. Contrary to Nb_16_Si_3_–CO_3_, Nb_16_Ge_3_–CO_3_ did not crystallize from a caesium germanate source, and only gels formed. However, sodium germanate (Na_4_GeO_4_) yielded Nb_16_Ge_3_–CO_3_ crystals, even though the addition of Na^+^ to Cs–Nb_6_ initially caused precipitation (due to the low solubility of Na^+^–PONbs).^33,34,47^ This result defies easy explanation because the sodium sites in the crystalline lattice are partially occupied, do not exhibit regular polyhedra, and do not connect the POMs within the ordered lattice. Oftentimes, when a specific alkali is the key to POM crystallization for mixed alkali lattices, its role is evident through its full occupancy in lattice positions that link the POMs into one, two or three dimensional lattices. The initial partial precipitation of Na^+^–PONbs may be a key step in crystal growth, followed by growth of the Nb_16_Ge_3_–CO_3_ crystals at the solid–liquid interface. This growth mechanism was studied indepth for Nb_16_Si_3_–CO_3_ and is discussed later. With CO_2_ infusion, this precipitate redissolves (Fig. S1), indicating that the Nb–CO_3_–POMs are more soluble than Nb_6_. Only gels were obtained with phosphate.

## Results and discussion

### Crystal structure descriptions

The Nb_16_Si_3_–CO_3_ and Nb_16_Ge_3_–CO_3_ lattices are not isostructural due to the Cs/Na^+^ countercations for the Ge-analogue and Cs^+^ countercations for the Si-analogue (Table S1 and S2). Nb_16_Si_3_–CO_3_ crystallizes in the monoclinic *C*2/*c* space group (*V* = 30 823.44(66) Å^3^, *Z* = 8), while Nb_16_Ge_3_–CO_3_ crystallizes in the triclinic *P*1̄ space group (*V* = 8194.86(21) Å^3^, *Z* = 2). This is also reflected in the distinct crystal morphologies (Fig. S2). All 24 countercations were located in the electron density maps for both lattices, meaning charge-balance did not necessitate attributing disordered protonation, as is often the case with prior-reported high-nuclearity PONbs,^29,48,49^ and BVS of the oxygens confirms this (between 1.5–2.2 Tables S3 and S4). We note the similarity of the PONbs topology to a jellyfish and will use this analogy as a convenient way to describe the structure (Fig. 1).

The Nb_6_-unit, the Lindqvist ion,^50^ is a superoctahedron of six edge-sharing octahedra. A 7th NbO_6_-octahedron that shares three edges with the Nb_6_ core comprises the Nb_7_-unit (Nb_7_O_22_, Fig. 1a), which is always stabilized by further polymerization; *i.e.*, in Nb_24_.^37^ Nb_7_ resembles the gastrovascular cavity, commonly known as the head of the jellyfish. The three tentacles are linear trimers of pentagonal bipyramidal Nb(CO_3_)_2_O_3_. The Nb_7_-unit plus three directly bonded Nb–carbonate polyhedra comprise Nb_10_–carbonate, [Nb_10_O_25_(CO_3_)_6_]^12−^ (Fig. 1b) previously isolated from the reaction between Nb_10_ and K_2_CO_3_.^51^

A key feature of the PONb is the X_3_O_3_ (X = Si, Ge) ring coordinated inside the three tentacles and oxo-bridged to the 2nd and the 3rd Nb(CO_3_)_2_O_3_ unit of the tentacles (Fig. 1c, e and f). The Si–O bond lengths range from 1.58–1.64 Å (avg = 1.62 ± 0.02 Å), and the Ge–O bond lengths range from 1.67–1.76 Å (avg = 1.73 ± 0.03 Å, Table S5). The difference in XO_4_ bond lengths is accommodated by the Nb–O–Nb bond angles between the 2nd and the 3rd Nb(CO_3_)_2_O_3_ units, which are smaller for the Si-analogue (avg = 144.8°) compared to the Ge-analogue (avg = 150.3°, Table S6 and Fig. 1e). The bond distances for the X_3_O_9_ rings of prior-reported Cs_6_X_3_O_9_ phases that also feature the trimer ring^45,46^ are quite similar (Table S5). The average Nb–O bond distances within the Nb_7_-unit are ∼1.76 Å, ∼2.40 Å, and 1.89–2.40 Å, respectively, for Nb=O_yl_, µ_6_-O-Nb, and µ_2_-O-Nb. Interestingly, the Nb–O bonds down the length of the three tentacles alternate between short and long, ∼1.8 Å and ∼2.2 Å (Fig. S3), preserving the second-order Jahn–Teller distortion. This also suggests the tentacles may be labile in solution, explaining the facile conversion to Nb_10_–carbonate, discussed later. The equatorial Nb–O_carbonate/oxo_ bonds perpendicular to the aforementioned axial bonds of these pentagonal bipyramids are consistently ∼2.0–2.1 Å.

Arrangement of Nb_16_Si_3_–CO_3_ clusters within the crystalline lattice is best viewed down the *b*-axis (Fig. S4a and b). The clusters are aligned in rows along the *a*-direction, and the rows are stacked in the *c*-direction, with the jellyfish heads pointing in opposite directions in every other row. Cs^+^ cations provide both head-to-head and tentacle-to-tentacle linking in the *c*-direction. The lattice organization of Nb_16_Ge_3_–CO_3_ is notably different than that of Nb_16_Si_3_–CO_3_. Rows of POMs are best observed along the *a*-axis (Fig. S4c and d) and are aligned in the (011) direction. The direction of the head alternates along these rows. In the perpendicular direction (approximately (01−1)), the POMs are arranged in pairs, tentacle-to-tentacle. Like the Nb_16_Si_3_–CO_3_ lattice, the clusters are bridged by Cs^+^ cations.

Of the 24 (Nb_16_Si_3_–CO_3_) and 21 (Nb_16_Ge_3_–CO_3_) Cs^+^ cations, three are bonded entirely between the tentacles, and three are bonded to both the tentacles and lattice water (Fig. 1d and S5). Cs^+^ cations fully bonded to the POM oxygens (Table S7) exhibit a crown-like arrangement. These six bonded Cs^+^ reflect the pseudo-trigonal arrangement of the tentacles, observed in Fig. S5.

### Probing Cs^+^ coordination in solution

**Fig. 3:**
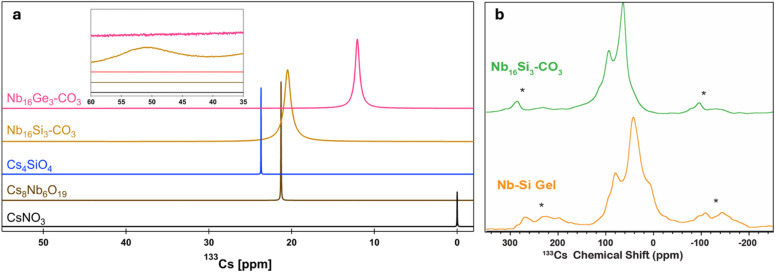
(a) Solution and (b) MAS ^133^Cs ssNMR spectra of Nb_16_X_3_–CO_3_. (a) Full spectrum of reference materials and redissolved Nb_16_X_3_–CO_3_ crystals. Inset of (a) shows the second, broad peak downfield from the primary ^133^Cs peak (55 ppm) in Nb_16_Si_3_–CO_3_ that is absent in the Ge analogue. (b) MAS ^133^Cs spin echo ssNMR spectra of Nb_16_Si_3_–CO_3_ (green) and Nb–Si gel (orange), revealing two distinct Cs environments, consistent with solution. All ^133^Cs ssNMR spectra were acquired at 9.4 T with a MAS frequency of 10 kHz. Asterisks denote spinning sidebands.

Solution (Fig. 3a) and solid-state (Fig. 3b and Table S8) ^133^Cs NMR spectra differentiate these coordination environments from those bonded solely to lattice water (solid-state) or free in solution. Solution ^133^Cs NMR of Nb_16_Si_3_–CO_3_ redissolved in water (0.1 M Nb_16_Si_3_–CO_3_) exhibits a major broad peak at 20.5 ppm (FWHM = 62.1 Hz), referenced to 0.1 M CsNO_3_ (0 ppm) (Fig. 3a and Table S9). Expanding the baseline reveals a second, even broader peak at 51.2 ppm (FWHM = 451.5 Hz). It was not possible to accurately integrate these peaks. However, based on comparison to the ^133^Cs ssNMR spectra, the peak at 51.2 ppm is attributed to Cs^+^ bonded to the PONbs in the crown-like coordination environments created by the tentacles. For comparison, a 0.1 M solution of Cs–Nb_6_ exhibits a similar ^133^Cs peak position as the free Cs for Nb_16_Si_3_–CO_3_, but it is much sharper (21.3 ppm, FWHM = 3.74).

Variable-temperature ^133^Cs solution NMR spectra recorded at 5, 10, 25, 35, 45, and 55 °C (Fig. S6 and Table S9) yielded exchange information. At 5 and 10 °C, the broad peak at ∼51 ppm sharpens (FWHM = 423.4, 432.2, respectively). The NMR signal broadens at higher temperatures, due to chemical exchange of Cs^+^ ions between binding sites on the POM and free, solvated Cs^+^ ions. The broad peak disappears entirely when heated to 35 °C, indicating that the POM-bound Cs^+^ exchanges rapidly with free Cs^+^ or is completely dissociated from the POM. Consistent with this observation, the primary ‘free’ Cs^+^ peak shifts from 20.5 to 17.9 ppm toward the CsNO_3_ peak position (Fig. S6). Cooling Nb_16_Si_3_–CO_3_ to 25 °C after heating shifts the primary peak back to its original position (20.2 ppm). However, the broad peak at ∼53 ppm is barely visible, indicating that Cs^+^ exchange is not fully reversible. SAXS confirmed that the cluster doesn't change with heating, as its scattering pattern closely resembled that of the freshly prepared Nb_16_Si_3_–CO_3_ solution (Fig. S7).

Magic angle spinning (MAS) ^133^Cs solid-state NMR (ssNMR) experiments were performed on Nb_16_Si_3_–CO_3_ to validate the ^133^Cs chemical shift assignments made by solution NMR spectroscopy. The MAS ^133^Cs ssNMR of Nb_16_Si_3_–CO_3_ shows two broad peaks centred at chemical shifts of 94 ppm and 63 ppm, with the lower frequency signal being most intense (Fig. 3b). ^133^Cs ssNMR spectra recorded at two fields show similar peak widths (Fig. S8), suggesting that the ^133^Cs ssNMR spectra consist of nearly isotropic NMR signals, where changes in the peak positions may be interpreted primarily as differences in chemical shift arising from changes in the local chemical environment of the Cs^+^. Based upon the solution ^133^Cs NMR results, single-crystal X-ray structure, and relative peak intensities, we assign the higher-frequency ^133^Cs NMR signal to Cs^+^ in the crown-like coordination environments created by the tentacles and the lower-frequency ^133^Cs NMR signal to the lattice Cs^+^ ions.

In contrast, solution ^133^Cs NMR of Nb_16_Ge_3_–CO_3_ only shows a single peak at 12 ppm (Fig. 3a), even upon decreasing the temperature (Fig. S9). In addition, the chemical shift of the Cs^+^(aq) peak is shifted to lower frequency and is narrower compared to the analogous Nb_16_Si_3_–CO_3_ peak (12 ppm, 42.2 Hz), similar to CsNO_3_. These observations indicate weaker association of Cs^+^ with Nb_16_Ge_3_–CO_3_ in solution, compared to the silicate analogue. The similar Cs–O bond lengths and coordination number cannot explain the lack of two distinct Cs^+^ environments in the solution NMR spectra for Nb_16_Ge_3_–CO_3_ (Table S9). However, concentration differences play a role. The Nb_16_Ge_3_–CO_3_ measurement was necessarily performed at a lower concentration, due to its poorer solubility, even with added quaternary ammonium. Lower solubility is attributed to the Na^+^ countercations that decrease PONb solubility.^31,33,34^

^133^Cs ssNMR spectroscopy was also used to investigate the structure of the gel-phase POM, with crystalline Nb_16_Si_3_–CO_3_ as a reference point (Fig. 3b). The ^133^Cs ssNMR spectrum of Nb–Si gel shows two distinct NMR signals, with similar intensity patterns as observed for Nb_16_Si_3_–CO_3_. However, the peaks for the gel are shifted to lower frequencies (*δ*_iso_ = 79 and 40 ppm) compared to the crystalline Nb_16_Si_3_–CO_3_. The reduced chemical shift for the NMR signals of the gel suggests that there is a change in the local structure of the Cs^+^ ions, possibly due to incorporation of additional waters of hydration around the Cs^+^ ions within the gel phase. The ^133^Cs NMR signals are also broader than those observed for the crystalline Nb_16_Si_3_–CO_3_ phase, suggesting increased disorder in the gel phase, consistent with its amorphous structure. However, the observation of two distinct groups of ^133^Cs NMR signals suggests that Nb_16_Si_3_–CO_3_ also dominates the gel phase, and some of the Cs^+^ ions remain coordinated to the tentacles within the gel. The ^29^Si ssNMR spectra discussed later agrees with this interpretation.

### SAXS of Nb_16_Si_3_–CO_3_ and Nb_16_Ge_3_–CO_3_

SAXS of the Nb_16_Si_3_–CO_3_ and Nb_16_Ge_3_–CO_3_ redissolved crystals was limited to 5 mM POM due to the high X-ray absorption of caesium (Fig. S10). Comparison of the experimental scattering curves with various simulated scattering curves of Cs-associated POMs derived from the lattice implies extensive non-specific connectivity of the POMs by the caesium cations. The scattering curve slope never reaches a plateau at low *q* (*i.e.*, *q*_0_), suggesting polydispersity due to aggregation. A size distribution analysis was performed on both Nb_16_Si_3_–CO_3_ and Nb_16_Ge_3_–CO_3_ SAXS with Irena,^52^ using a spheroid form factor to estimate aggregate sizes (Fig. S11). The smallest scatters have average diameters of 10.3 and 10.7 Å for Nb_16_Si_3_–CO_3_ and Nb_16_Ge_3_–CO_3_, respectively. The POM is ∼14 × 7 Å, as measured from Nb to Nb in the crystal structure (Fig. S11); consistent with the average scattering diameter of ∼10 Å. Larger aggregates, determined from the size distribution, have diameters ranging from 19 to 55 Å for Nb_16_Si_3_–CO_3_, whereas Nb_16_Ge_3_–CO_3_ shows two distinct populations with diameters of 25 and 37 Å.

The experimental scattering curve is always dominated by the largest aggregates over the non-associated POM because *I*(*q*)*α* (particle volume)^3^, compared to *I*(*q*)*α* (particle concentration)^1^.^53^ Therefore, the simulated scattering for the single POM does not match that of the experimental scattering, even though the size distribution suggests these smaller clusters are the most abundant in solution.

The Guinier elbow for Nb_16_Si_3_–CO_3_ scattering most closely matches that of the simulated hexamer aggregate with a maximum diameter of 65 Å (Fig. S10 and S11). Nb_16_Ge_3_–CO_3_ scattering is most similar to the simulated tetramer aggregate scattering, with a maximum diameter of 40 Å (Fig. S10 and S11). This is consistent with the data-fitting results, which show that the probability of scattering approaches zero at a diameter of 60 Å. In summary, the dissolved crystals form large networks of Cs-connected clusters, which are also important for the formation of the gels, as discussed later.

### Assembly pathway

#### ^29^Si NMR – crystalline conditions

Solution and solid-state ^29^Si NMR spectra provide information about the assembly pathway of Nb_16_Si_3_–CO_3_ and, by inference, Nb_16_Ge_3_–CO_3_. For reference, a 0.1 M Nb_16_Si_3_–CO_3_ solution was prepared by dissolving crystals in 90–10 H_2_O–D_2_O, yielding a solution ^29^Si NMR spectrum with a single peak at −88.6 ppm, which we attribute to Si_3_O_9_ bound within the PONb.

**Fig. 4:**
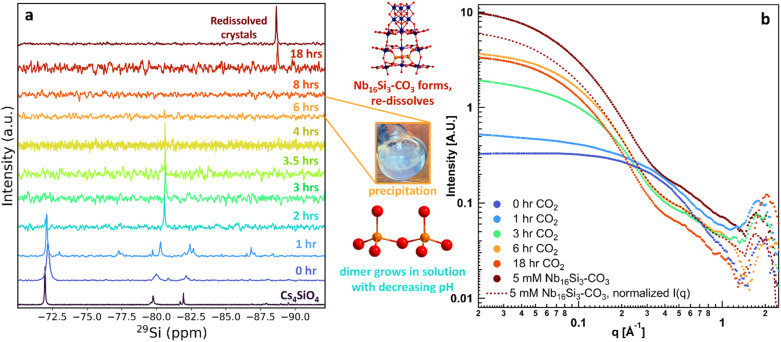
Solution state reaction pathway of Nb_16_Si_3_–CO_3_ tracked by (a) solution ^29^Si NMR spectra and (b) SAXS patterns. Red spheres are oxygen, orange spheres are silicon, blue spheres are niobium.

The ^29^Si NMR spectrum of stock Cs_4_SiO_4_ shows several peaks that are assigned to [SiO_4_]^4-^ (−71.9 ppm), plus minimal dimers [Si_2_O_7_]^6−^ (−79.7 ppm) and trimers [Si_3_O_9_]^6−^ (−81.7 ppm) (Fig. 4a).^54–56^ There are also very minor peaks around −90 ppm, consistent with higher-order oligomers.^57^ Introducing Cs–Nb_6_ (*t* = 0 h CO_2_) to the silicate solution broadens and slightly shifts the ^29^Si peak (Table S10 and Fig. S12 for full spectral window) compared to pristine Cs_4_SiO_4_. After 2 hours of CO_2_ infusion, the dimer peak at −80.5 ppm becomes dominant, indicating polymerization of monomers,^54–56^ followed by complete disappearance of this peak after 8 hours of CO_2_ exposure. Visual inspection of the NMR tube showed the presence of a precipitate. The gel-like precipitate (Fig. S13a) persists if the reaction is concluded before 18 hours and redissolves if the reaction is left to proceed to crystallization *via* continued CO_2_ exposure. After 18 hours, a ^29^Si NMR signal re-emerges, shifted to −88.7 ppm, precisely matching that of redissolved Nb_16_Si_3_–CO_3_ crystals, and concomitant with redissolution of the precipitate. This indicates complete PONb assembly, followed by crystallization (∼80% yield, see SI).

In a control experiment, the Cs_4_SiO_4_ solution (without Nb) precipitates upon exposure to CO_2_ (Fig. S13b). The solution ^29^Si NMR of the Cs_4_SiO_4_ solution after CO_2_ exposure shows that the monomer persists with no evidence for dimerization after 4 hours of CO_2_ exposure (Fig. S14). We conclude that the solution conditions with the PONb provide some stabilization of small oligomers, *i.e.*, dimers.

We compared the precipitate from the CO_2_-infused Cs_4_SiO_4_ solution to the intermediate precipitate from the CO_2_-infused Nb_16_Si_3_–CO_3_ reaction solution. The precipitate from the Cs_4_SiO_4_ solution has a Cs : Si ratio of ∼1 : 1 (determined from SEM-EDS analysis, Fig. S15) and is X-ray amorphous (Fig. S16). Both the Cs : Si ratio and the ssNMR chemical shift^54,58^ suggest the silica is predominantly the octamer cubane [Si_8_O_20_]^8–^ (Fig. S17), originally crystallized as TMA_8_[Si_8_O_20_].^58,59^ The ^29^Si ssNMR spectrum (Table S11 and Fig. S18) of the precipitate obtained from the CO_2_-infused Cs_4_SiO_4_ solution reveals a broad resonance centred at −96 ppm with a distinct shoulder near −89 ppm. The NMR signal at −96 ppm is assigned to the [Si_8_O_20_]^8−^ units, while the higher frequency (less negative chemical shift) signals are assigned to incompletely condensed dimeric or trimeric silicate units, which likely precipitate over the course of the reaction.

The precipitate from the Nb_16_Si_3_–CO_3_ reaction solution (6-hour time stamp, where no solution ^29^Si peak is observed, Fig. 4a) was challenging to analyse due to difficulty in separating the Nb-rich solution from the precipitate before Nb_16_Si_3_–CO_3_ crystallized. SEM images of this gel-like precipitate, with crystals nucleating on the surface, are shown in Fig. S19. Multipoint SEM-EDS analyses of the regions of the precipitate that appear crystalline (points 3 & 8) and amorphous (points 1, 2, and 6) are shown in Fig. S20 and summarized in Table S12. The amorphous regions have Nb : Si ratios of approximately 1 : 1, while the crystalline regions have Nb : Si ratios of approximately 4.5 : 1. This supports our hypothesis that the amorphous precipitate is caesium silicate with minimal niobium. Anecdotally, if this gel-like precipitate is removed from the mother liquor prior to crystallization, the yield is either much poorer, or no crystal growth occurs. These data, taken together, suggest that the POM tentacles support the dissolution and assembly of otherwise insoluble Si_3_-rings, which may, in turn, template the assembly of the tentacles.

Parallel SAXS analysis of the Nb_16_Si_3_–CO_3_ reaction solution during CO_2_ infusion and in contact with the silicate precipitate provided additional insights (Fig. 4b). Initially, the scattering is poor due to X-ray absorption from the high Cs concentration in the presence of smaller scatterers (Nb_6_). With CO_2_ infusion, the scattering intensity increases rather than decreases, despite loss of silicate from the solution. The Guinier elbow shifts from ∼0.3 Å^−1^ to 0.06 Å^−1^, indicating growth of larger PONbs with CO_2_ infusion. At 6 hours of CO_2_ exposure, when silicate is completely absent from the solution, the *I*_0_ (at *q* = 0.02 Å^−1^) reaches its maximum (Table S13). At 18 hours, the *I*_0_ decreases slightly due to the redissolution of the Cs silicate, which increases beam attenuation. Various analyses of the 6-hour and 18-hour CO_2_ exposure solution SAXS showed them to be very similar, with a slight increase in size for the 18-hour CO_2_ exposure. For example, PDDF (pair distance distribution function) analysis yielded a radius of gyration (*R*_g_) of 17 Å at 6 hours and 20 Å at 18 hours (Fig. S21a). As discussed above, the redissolved crystals form aggregates of approximately six POMs by Cs-linking, and size information is not meaningful except for comparison with related solutions. One additional SAXS simulation we performed was the Nb_16_Si_3_–CO_3_ cluster with and without the Si_3_-ring (Fig. S21b). The difference is minimal, as expected. Finally, in Fig. 4b, we compare SAXS of the time-dependent CO_2_-infused Nb_16_Si_3_–CO_3_ reaction solutions to that of redissolved crystals. There is a good match up to *q* ∼ 1.5 Å^−1^, but a continued rise in intensity at *q* < 1.5 Å^−1^ for the redissolved crystals. This suggests that similar species are present in the reaction solution and the redissolved crystals, with greater aggregation in the redissolved-crystal solution.

To summarize the Nb_16_Si_3_–CO_3_ assembly study, (1) CO_2_ infusion promotes [Si_2_O_7_]^6−^, [Si_3_O_9_]^6−^, and [Si_8_O_20_]^8–^ formation from [SiO_4_]^4−^ by acidification, followed by precipitation as a Cs-salt. (2) Simultaneously, PONbs evolve to primarily Nb_10_–CO_3_ and Nb(CO_3_)_2_O_3_ monomer units. (3) Symbiotic assembly of Nb_16_Si_3_–CO_3_ occurs with tentacle formation (Nb(CO_3_)_2_O_3_ chains), which assemble and sequester the Si_3_O_9_ trimers. Because Si_3_O_9_ is otherwise insoluble in these solutions, we presume the assembly occurs at the gel–solution interface, representing a sort of non-classical crystal growth that is now widely recognized in crystallization of natural and synthetic materials.^60,61^

### Structure and composition description of the PONb–X gels

By simply dissolving SiO_2_ or GeO_2_ in Cs–Nb_6_ (rather than with excess alkali) and following the same CO_2_ exposure regime, we obtain clear, rigid gels rather than crystals. Phosphate was added to aqueous Cs–Nb_6_ as CsOH-neutralized H_3_PO_4_, and this solution also formed a transparent gel. PONbs incorporating phosphate were never crystallized; only the previously described Nb_10_–CO_3_ and Nb_22_–CO_3_ POMs were obtained from these solutions.^41,51^

Here, we consider the driving forces of crystallization *vs.* gelation. The major difference between solutions from which crystals or gels are derived is the addition of excess CsOH or NaOH (crystallization) or not (gelation). The relative amounts of Cs (+Na), Nb, Si(Ge), explicitly added hydroxide and pH are summarized in Table 1, and detailed in the SI.

**Table 1:** Relative equivalents (rounded) of ions in reaction solutions for crystals and gels, normalized to Si or Ge

Phase	Cs	Nb	Si/Ge	Cs : Nb	Added OH	pH^b^
Nb_16_Si_3_–CO_3_ crystals	15	8	1	1.9	4	14.0/10.0
Nb–Si gel	4	3	1	1.3	0	14.0/11.5
Nb_16_Ge_3_–CO_3_ crystals	24^a^	15	1	1.6	4	13.5/10.0
Nb–Ge gel	17	13	1	1.3	0	13.0/10.0

a 4Na + 20 Cs.

b Before/after CO_2_ infusion.

In addition to explicitly added hydroxide (in the form of CsOH or NaOH), hydroxide is present from the self-buffering behaviour of the basic Nb-POMs and XO_4_^4−^ oxygens (X = Si, Ge), for example:

1 Nb_6_O_19,aq_^8−^ + H_2_O → Nb_6_(OH)O_18,aq_^7−^ + OH^−^

2 SiO_4,aq_^4−^ + H_2_O → Si(OH)O_3,aq_^3−^ + OH^−^

This protonation behaviour, *i.e.*, hydrolysis, can be followed by condensation *via* water release, *i.e.*:

3 2[Nb_6_(OH)O_18_^7−^]_aq_ → [Nb_6_O_18_^7−^–O–Nb_6_O_18_^7−^]_aq_ + H_2_O

The behaviour is especially prevalent for Nb-POMs, given their high negative charges. Explicitly added hydroxide works counter to the self-buffering process; *i.e.*:

4 [Nb_6_(OH)O_18_^7−^]_aq_ + OH^−^ → Nb_6_O_19,aq_^8−^ + H_2_O

Therefore, added hydroxide prevents POM and silicate polymerization that drives polydispersity and challenges crystallization. On the other hand, with less hydroxide, forming oxo-bridges between POMs and silicates promotes network formation and gelation.

The crystallization conditions also have higher alkali : Nb-POM ratios compared to gelation conditions (Table 1), and alkali concentration also dictates crystallization *vs.* gelation paths. It is noteworthy in Table 1 that the explicitly added base (crystal-growth conditions) does not necessarily result in higher pH. This suggests the self-assembly and self-buffering behaviour of the oxoanions has a stronger influence on the recorded pH, and other factors such as alkali concentration must be considered. More alkalis shield repulsion between anionic POMs, increase contact ion-pairing with anionic POMs, and bridge anionic POMs, leading to crystallization. Alkalis also replace protons on the POMs, whereas the protonated state favours gelation.

Compositions of the characterized gels are 49% Cs, 14% Si, 37% Nb (Nb–Si gel), 55% Cs, 3.2% Ge, 41% Nb (Nb–Ge gel), and 53% Cs, 6.8% PO_4_, 40% Nb (Nb–PO_4_ gel) (Table S14). These compositions were chosen primarily based on the maximum solubility of SiO_2_ and GeO_2_ in the concentrated Nb_6_ solutions, and optimized for gelation (*i.e.*, below a particular concentration of Si, Ge, or P, gelation does not occur). Gelation also occurs without direct CO_2_ introduction *via* evaporation. However, it takes much longer, *i.e.*, 10–14 days instead of 24–48 hours with CO_2_.

**Fig. 5:**
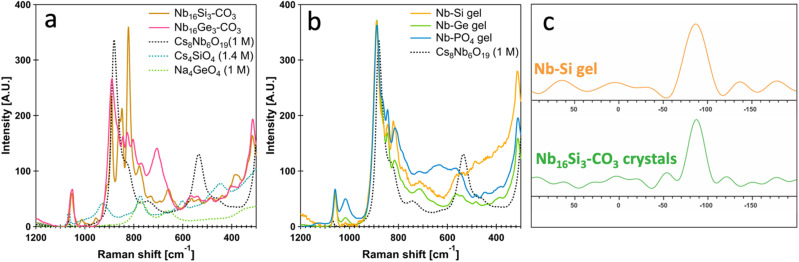
Raman spectra of (a) Nb_16_Si_3_–CO_3_ and Nb_16_Ge_3_–CO_3_ crystals and (b) the Nb–X gels. (c) MAS ^29^Si ssNMR spectra Nb_16_Si_3_–CO_3_ crystals and Nb–Si gel. The ^29^Si chemical shift of the Nb_16_Si_3_–CO_3_ crystals in solid-state is similar to that observed in solution. The nearly identical ^29^Si chemical shift in the Nb–Si gel indicates that the Si_3_O_9_ trimer remains intact within the gel network and likely binds to niobium, meaning the ‘jellyfish’ structure is retained within the gel network.

The Raman spectra of Nb_16_Si_3_–CO_3_ and Nb_16_Ge_3_–CO_3_ show new features in the ∼900–650 cm^−1^ range, distinct from the Nb–O bonding environments in Cs–Nb_6_ (Fig. 5). These arise from the Nb_10_–CO_3_ unit as well as the Nb(CO_3_)_2_O_3_ tentacles (Tables S15 and S16). Detailed peak assignments are provided in the SI but are complicated by overlapping features of different Nb–O and X–O bonding frequencies. The most intense peak in the Raman spectrum of Nb_16_Si_3_–CO_3_ (822 cm^−1^) is also present in the Nb_16_Ge_3_–CO_3_ spectrum, although it is less prominent. This peak correlates with bonding modes of the PONb and X_3_O_9_ ring (*i.e.*, Nb–O–X), since the Raman frequency does not match any prior reports for the alkali-Si_3_O_9_ trimer alone. Despite this, multiple Raman frequencies in the Nb_16_Si_3_–CO_3_ and Nb_16_Ge_3_–CO_3_ spectra match those of previously reported trimer phases^62–65^ in the 780–440 cm^−1^ region (Tables S15 and S16). These features are likewise present in the Nb–Si and Nb–Ge gel phases (Fig. S22), showing the X_3_O_9_ observed in the crystalline state is also present in the gel networks. All three gels have bands at ∼900–880 cm^−1^, attributed to PONbs mixtures including Nb_10_–CO_3_, Nb_22_–CO_3_, and Nb_16_X_3_–CO_3_. In fact, Nb_10_–CO_3_, closely related to Nb_16_X_3_–CO_3_, crystallized from spin-coated thin films of the Nb–Ge gel (Fig. S23, confirmed by SCXRD).^41^

^29^Si ssNMR spectroscopy confirmed the preservation of the Si_3_O_9_ trimer motif in the Nb–Si gel (Fig. 5c). The direct excitation ^29^Si NMR spectrum for both crystalline Nb_16_Si_3_–CO_3_ and Nb–Si gel exhibits a single ^29^Si NMR signal centred at −87 ppm (Fig. 5c). We note that the broadening of the ^29^Si NMR signals of Nb_16_Si_3_–CO_3_ and Nb–Si gel is not necessarily reflective of disorder or the presence of multiple independent Si sites in these samples. The spectra were acquired with CPMG detection to enhance sensitivity. The truncation of the individual spin echoes in the CPMG train results in signal broadening.^66^ Therefore, we cannot conclude that the Nb–Si gel is more disordered based upon the observed ^29^Si peak widths. Stirring the gel monoliths in warm DI water (∼60 °C) overnight led to dissolution (*i.e.*, gelation is reversible). This further validates that the PONbs are intact, as we do not expect oxides to redissolve in neat water.

The Raman spectrum of the Nb–PO_4_ gel exhibits Nb–O stretches of POMs, similar to those of the Nb–Si and Nb–Ge gels. The phosphate species do not appear to be self-polymerized, unlike silicate and germanate (Table S17).^67–70^ The Raman peaks from 866–1016 cm^−1^ in the Nb–PO_4_ are consistent with PO_4_ encapsulated in niobate Keggin ions.^71^ Solution-phase ^31^P NMR (prior to gelation; Fig. S24a, b, Tables S18 and S19) confirms this. The major peak at 2.5 ppm coincides with [PNb_12_O_40_]^15−^, while the minor peak at 5.2 ppm corresponds also with a phosphate-centred Keggin ion, but likely one that is bi-capped with niobyl (*i.e.*, [PNb_14_O_42_]^7−^).^72^ Finally, the broad peak indicates polymerization of phosphorus-centred Keggin ions, *i.e.*, *via* the Nb=O caps or Nb_2_O_2_ bridges.^73^

The ^31^P ssNMR of the Nb–PO_4_ ‘wet’ gel features a sharp NMR peak with a broad shoulder at 2.59 ppm ([PNb_12_O_40_]^15−^) and −1.77 ppm (Fig. S24c). Upon drying the gel overnight at 900 °C, the two NMR signals remain but are shifted to a higher frequency at 4.3 ppm and 0.0 ppm, respectively. The retention of the NMR lineshape in both spectra suggests that the local environment of the PONb around PO_4_ is retained within the gel.

The minor broader feature at −1.8 ppm in the wet gel (∼0 ppm in the dry gel) identifies a phosphorous environment not observed in solution, before drying. ^1^H → ^31^P cross polarization (CP) NMR experiments performed on the dried gel^74,75^ with varying the CP contact time reveal an increase in intensity of the 0 ppm NMR signal at shorter contact times, indicating a protonated environment. Meanwhile, the Keggin-centred phosphate peak is unchanged. Because a Keggin-centred phosphate cannot be protonated, we assume the Nb–P gels also contain phosphates that link the POM units, more similar to traditional phosphate glasses. This differs from the Nb–Si gel that features only polymerized silicate within the PONb units.

Oligomeric silicate, germanate, phosphate, and intact PONbs are also seen in the FTIR spectra (Fig. S25 and Tables S20–22). There are FTIR features unique to the Nb_6_ unit, the common building block of each known Nb–carbonate POM (Nb_10_–CO_3_, Nb_22_–CO_3_, and Nb_16_X_3_–CO_3_). FTIR bending and stretching modes for the X_3_O_9_ (Nb–Si and Nb–Ge gels) match those reported in the literature.^62,64,65,69,70,76^

We used TGA-MS and CHN analyses to quantify the carbonate content of the crystals and gels. From DSC and TGA, we determined that the weight loss from 25 °C to 200 °C is due to water, either from the surface or lattice/network-bound (Fig. S26). The CO_2_ release temperature from TGA-MS-DSC allowed identification of carbonate bonding, either with niobium or the alkali. Alkali carbonate (*i.e.*, Cs_2_CO_3_) is released at temperatures greater than 600 °C, while niobium-bound carbonate is released at lower temperatures, between 350–600 °C (Table S23), consistent with previous Nb–carbonate POMs.^41^ The Nb_16_Si_3_–CO_3_ and Nb_16_Ge_3_–CO_3_ crystals have similar CO_2_ release temperatures, but Nb_16_Ge_3_–CO_3_ has a broad CO_2_ peak with multiple release temperatures.

Comparing TGA-DSC-MS (Fig. S26) of crystalline Nb_16_Si_3_–CO_3_ and Nb_16_Ge_3_–CO_3_ to either the wet or dry gels, two trends emerge. First, there is a greater range of CO_2_ release temperatures for the crystals than for the gels, with the peaks being more distinct for the crystals, as expected for an ordered material. Second, in general, the crystals have lower release temperatures than the gel species, suggesting that some Nb-bound carbonate within the bulk crystals is less thermodynamically stable than in the gel system, with niobium sites acting as better catalysts to facilitate CO_2_ release. The Nb–Si and Nb–Ge wet and dry gels exhibit one or two CO_2_ release temperatures ranging from 460 to 580 °C (Table S23). Interestingly, the TGA-MS-DSC analysis of the Nb–PO_4_ wet gel shows no CO_2_ release temperature that could be considered as niobium-bound carbonate. As noted earlier, ^31^P NMR suggests PONbs in the pre-gel solutions are phosphate-centred Keggin ions (Fig. S24). However, the Nb–PO_4_ dry gel has two CO_2_ release temperatures associated with niobium, indicating at least partial conversion of the PNb_12_ Keggin ions and related species to Nb_10_–CO_3_ upon drying, also observed by vibrational spectroscopy.

Quantification of carbon content in each sample was performed by CHN analysis (Table S24). The weight percent carbon (in the form of carbonate, wt% C) for Nb_16_Si_3_–CO_3_ and Nb_16_Ge_3_–CO_3_ (2.9 and 2.8 wt% C, respectively) is close to what we expect based on the crystal structure (3.2 and 3.1 wt% C, respectively). The gel materials (including Nb–P gel) have slightly less carbon content than the crystalline materials (1.6 wt% C on average). This is not surprising, since the solutions for gel preparation are less basic than those for crystallization.

It is difficult to determine precisely how the gel networks change as they dry. Considering that the gels are supramolecular (*i.e.*, multiple components interacting non-covalently), they may react and change over time.^1,10,77,78^ We expect water vaporization leads to Nb–O–Nb bond formation *via* oxos or carbonate. However, there is substantial evidence that our transparent networks are mixed POMs, including those with carbonate ligands and those without (*i.e.*, PNb_12_), and their polydispersity distinguishes them from crystalline materials and is conducive to gelation.

Finally, Cs_6_Nb_6_O_19_, Cs_2_CO_3_, Nb_16_Ge_3_–CO_3_, Nb–Ge gel, and Nb–P gel all exhibit multiple sharp melting endotherms, especially around 800 °C. While this is known for Cs_2_CO_3_, it is generally not a known property of POMs. Low melting temperature is likely unique to POMs with soft alkali counter cations (*i.e.*, Cs, maybe Rb) and could be further exploited to prepare anhydrous POM glasses.

### Optical properties of the PONb–X gels

**Fig. 6:**
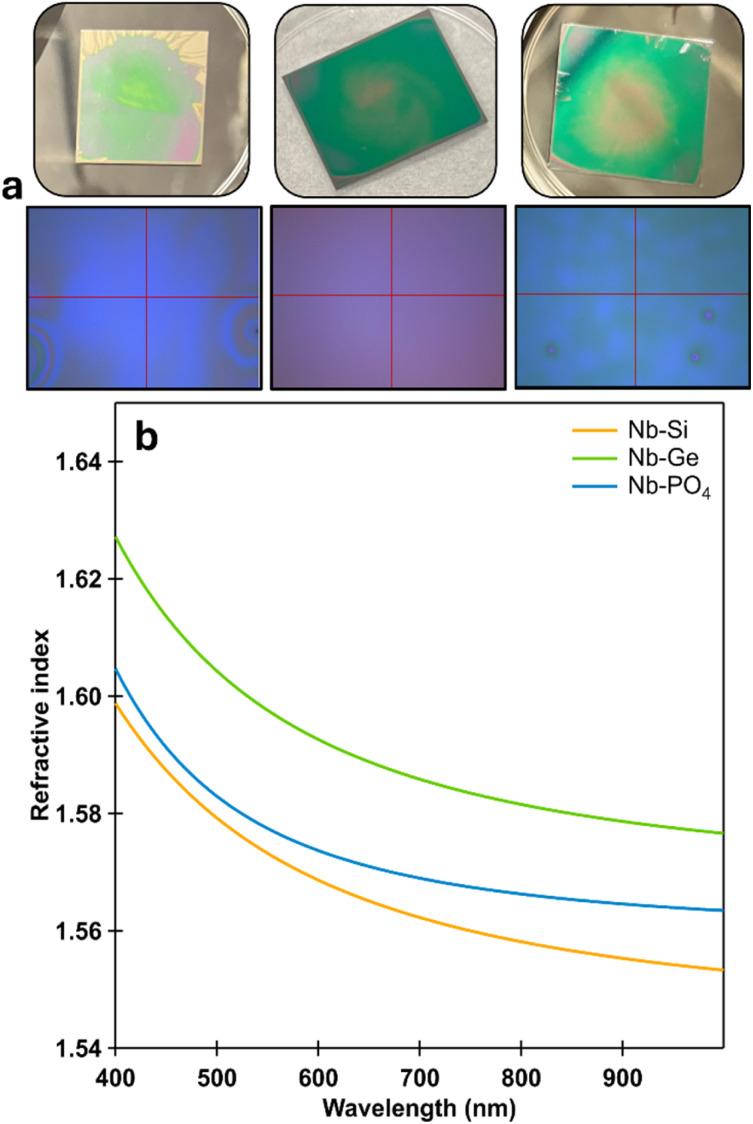
(a) Images of each Nb–X thin film, either the entire film (top), or focused to a specific area for measurements (bottom). (b) Dispersion curves of each Nb–X thin film.

Intrigued by the optical clarity of the gel monoliths, we investigated their properties as thin films (Fig. 6a) using spectroscopic ellipsometry (SE) and UV-vis spectroscopy. Traditional dispersion diagrams (Fig. 6b) show that all three gels exhibit a monotonic decrease in refractive index with increasing wavelength, consistent with normal dispersion behaviour observed in the visible to near-infrared spectral range. Among the films, Nb–Ge shows the highest refractive index across the entire wavelength range, while Nb–Si exhibits the lowest (Table S25). Notably, the Nb–Ge film also has the lowest surface roughness and the smallest thickness (∼806 nm). The combination of high refractive index and smooth surface suggests that Nb–Ge is a candidate for applications requiring enhanced optical confinement and minimal scattering loss. In contrast, the relatively thicker Nb–Si film may influence mechanical stability, thermal characteristics, or optical interference effects when used in multilayer coatings.

From SE, the *ψ* (psi) spectrum for the Nb–Si thin film exhibits pronounced and periodic oscillations characteristic of thin film interference effects (Fig. S27a). The *Δ* (delta) spectrum displays broad modulation with distinct interference features. Similar to the *ψ* response, a minor deviation from the model is evident at an incident angle of 55°, though it remains within acceptable limits. Additionally, the film demonstrates high optical transparency, with no significant absorption features detected across the visible to near-infrared (NIR) spectral range.

Clear interference fringes are observed in the *Ψ* spectra at both 55° and 60° angles of incidence for the Nb–Ge thin film (Fig. S27b). The fringe spacing is narrower at 55°, consistent with the expected dependence of optical path length on the angle of incidence. The oscillation amplitude remains similar across both angles, indicating that the film reflects a significant portion of light in both s- and p-polarizations. The excellent agreement between the experimental and modelled data confirms the accuracy of the extracted film thickness and refractive index values.

The Nb–P thin film exhibits well-defined and consistent interference oscillations in the *Ψ* spectra at both 55° and 60° angles of incidence, indicative of the film's high optical transparency (Fig. S27c). The *Δ* spectra similarly show interference-related modulations, with a subtle angular dependence. The experimental and model-generated curves are in excellent agreement, indicating the accuracy of the extracted refractive index (*n*) and the determined film thickness (Table S25). Across the measured spectral range (300–950 nm), the Nb–P films demonstrate high transparency and the absence of a pronounced absorption edge, as evidenced by the lack of abrupt phase shifts in the *Δ* response. These characteristics are consistent with the expected behaviour of dielectric oxides and phosphate-based materials.

### Electronic properties of the PONb–X gels

The optical transparency and lack of absorption in the UV-Vis spectra (Fig. S27d) of the gels prompted the measurement of dielectric properties. Niobium-based insulators commonly have high dielectric permittivity due to the second-order Jahn–Teller effects of Nb=O. The dielectric loss (tan(*δ*)) data (Fig. S28) for Nb–Si and Nb–Ge materials is high, >0.5 at all measured frequencies. Dielectric loss is a function of electronic, thermal, or ionic conduction that screens the applied field, preventing the activation of dipoles.

**Fig. 7:**
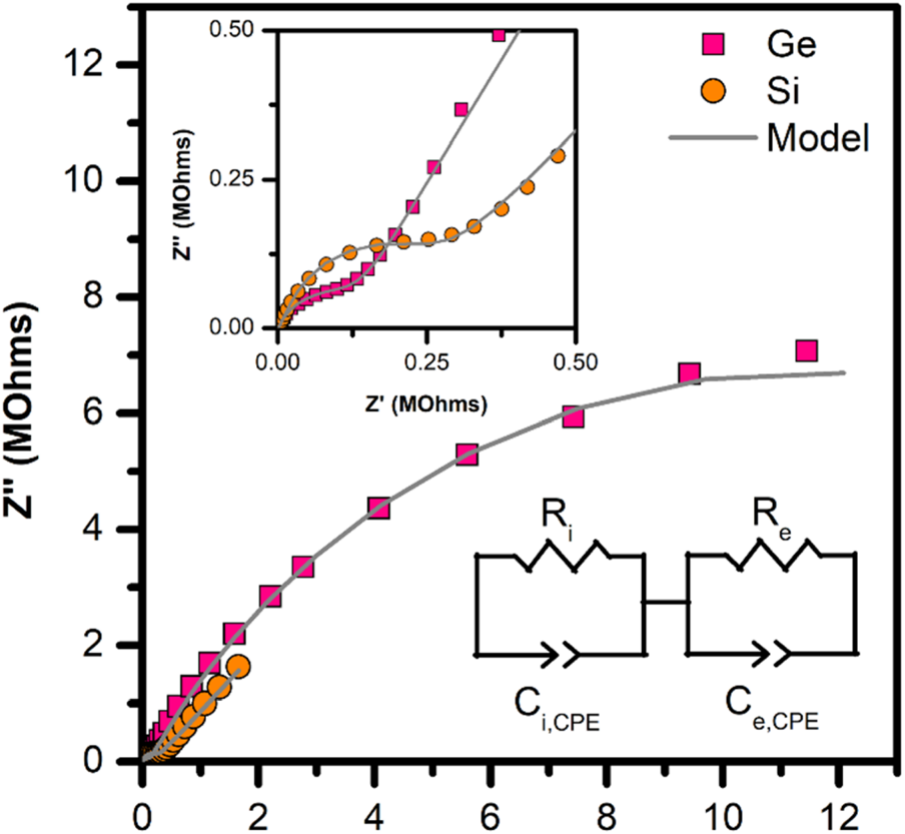
Room temperature impedance data for Nb–Si (orange circles) and Nb–Ge (pink squares) gels, plotted in the complex plane. Data are fit using an equivalent circuit, shown in the bottom right corner, and the fit line is shown in grey. The inset shows the high-frequency region, which has a semicircular feature.

To understand the high loss of these monoliths, impedance spectroscopy was measured. Impedance data in the complex plane for the Nb–Si and Nb–Ge are shown in Fig. 7 – the Nb–P gel could not be measured due to its hygroscopic behaviour. Both the Nb–Ge and Nb–Si data exhibit a small arc (Fig. 7 inset). The Nb–Ge sample has a larger arc, indicating two conductivity mechanisms present, due to the presence of two semicircles.

Further understanding of the electronic properties was developed by fitting the data with equivalent circuit models. An *R*–*C* circuit, a resistor and a capacitor in parallel, was used to model the first, smaller semicircle, followed by another *R*–*C* circuit to model the second, larger semicircle. These two circuits represent two conductivity mechanisms, which are presumed to be electronic and ionic conductivity.

The Nb–Ge gel shows a higher conductivity for both mechanisms. The *σ*_e_ is determined to be 3.8 × 10^−6^ S cm^−1^, and the Nb–Si gel has a similar *σ*_i_ of 8.3 × 10^−6^ S cm^−1^ (Table S26). While not particularly high, the ionic conductivity of both systems is similar to that of other POMs where the conductivity was measured.^79–81^ The mechanism is presumed to be ionic due to the presence of high concentrations of mobile species such as Cs^+^ and OH^−^. However, to isolate which ionic species is responsible; *p*_H_2_O_, variable temperature impedance, or line-broadening NMR experiments would be needed.

The second semicircle in Nb–Ge gel is believed to arise from electronic conductivity, and the *σ*_e_ = 2 × 10^−8^ is consistent with a lossy insulator.^82,83^ Our presumption is based on the extracted capacitance of 11 nF, which is high for a dielectric ceramic and is closer to that of a high-*K* or ferroelectric ceramic.^84^ This is consistent with the strong second-order Jahn–Teller effects of the d^0^ Nb^5+^ ion, which imparts ferroelectric and dielectric properties in many compounds, such as Nb_2_O_3_, LiNbO_3_, and BaNb_2_O_6_. The electronic conductivity is higher than expected for a material with closed-shell cations, a large optical bandgap, and exists as a clear gel. However, an amorphous structure is well known to produce higher conductivities relative to the crystalline analogue, a notable example of this being InGaZnO.^85–87^ The mechanism is understood to arise from a variety of metal coordination environments producing donor and acceptor states within the bandgap, thereby increasing the carrier concentrations without compromising the optical properties.

## Conclusions

Here we present new PONbs topologies (in high yield) where pH is controlled by CO_2_ infusion instead of the addition of aqueous acid/base. The PONbs are ligated with carbonate and encapsulate a Si_3_O_3_ or Ge_3_O_3_ trimer ring, a building block previously observed only in rare mineral phases or from high-temperature-high-pressure syntheses. Altering only the Cs-concentration, we obtain highly transparent gels instead that feature mixtures of PONbs. These were obtained with three different network formers/POM templates: germanate, silicate, and phosphate. While the germanate and silicate gel contain the same PONbs as the crystalline phases (or subunits thereof) plus carbonate ligands, the phosphate gels feature phosphate-centred Nb Keggin ions, and minimal carbonate ligands. While silicate and germanate locate only inside the POMs, the phosphate both templates Keggin ions and serves as a network former within the Nb–P gels. Alkali concentration is a crucial variable distinguishing gel and crystal formation, with the crystal-forming conditions having approximately 10–20% more alkali (Cs^+^) than the gel-forming conditions. This could be attributed to the salting-in/out effect. Low alkali concentration leads to salting in, enabling components to link into a sample-spanning network, while high alkali concentration leads to salting out into an ordered lattice. Additionally, higher alkali plus associated hydroxide concentration leads to high basicity, preventing condensation reactions that also link components together into an extended network. Our next steps will include two paths to solvent-free inorganic glasses that preserve POMs and endow polarizability from the Nb=O Jahn–Teller distortion, characteristic of all POMs. Strategies will include solid-state routes exploiting the low melting temperatures observed in the current study, and countercations that are volatilized at a low temperature.

## Author contributions

M. N. conceptualized and supervised the project, acquired funding, and aided in the formal data analysis and writing. M. T. N. conducted the investigation, collected the data, and led the writing. A. P. P. and A. J. R. acquired ssNMR data, interpreted data, and contributed to writing. N. D. J. A. and R. M. contributed to the collection, analysis, and writing of optical spectroscopic data. K. B. and E. H. assisted with data collection, and E. H. designed the TOC. W. T. S. collected, analysed, and assisted with the writing of the impedance spectroscopy data.

## Conflicts of interest

There are no conflicts to declare.

## Supplementary Material

SC-OLF-D6SC02933G-s001

SC-OLF-D6SC02933G-s002

## Data Availability

CCDC 2494764 (Nb_16_Si_3_–CO_3_Nb_16_Si_3_–CO_3_) and CCDC 2494765 (Nb_16_Ge_3_–CO_3_) contain the supplementary crystallographic data for this paper.^88*a*,*b*^ The supporting data has been provided as part of the supplementary information (SI). Supplementary information: synthesis, instrumentation details and presentation of data and images including NMR, SAXS, SCXRD, SEM/EDS, Raman and FTIR, TGA, CHN analysis, optical spectroscopy and conductivity measurements in Fig. S1–S29 and Tables S1–S26. See DOI: https://doi.org/10.1039/d6sc02933g.
