# Supplementary material for: Co-templating of polyoxoniobates and silicate/germanate trimer-rings in crystals and inorganic gels
Source: Chem Sci. 2026 May 19;17(25):12558–70. doi: 10.1039/d6sc02933g (PMC13185001; doi:10.1039/d6sc02933g)
Supplement: SC-017-D6SC02933G-s001 [file SC-017-D6SC02933G-s001.pdf]

Supplementary Information (SI) for Chemical Science.
This journal is © The Royal Society of Chemistry 2026

Supplementary Information

Co-templating of polyoxoniobates and silicate/germanate trimer-rings in crystals and inorganic gels

Makenzie T. Nord,[a,b] Andrew P. Porter,[c,d] Detcho J. N. Aboa,[†e] Robert S. Minning,[†e] Karlie Bach,[a] Emily Hiatt,[a] Wesley T. Surta,[f] Lev N. Zakharov,[a] Aaron J. Rossini,[c,d] May Nyman[a]*

[*]*Corresponding author*

† *These authors contributed equally.*

a. *Department of Chemistry, Oregon State University, Corvallis, OR, 97331, USA. E-mail: may.nyman@oregonstate.edu*
b. *Current address: Nuclear and Radiochemistry (C-NR), Los Alamos National Laboratory, Mail Stop J-514, Los Alamos, NM, 87545, USA.*
c. *Ames National Laboratory, Ames, Division of Materials Science and Engineering, Iowa, 50011, USA*
d. *Department of Chemistry, Iowa State University, Ames, IA, 50011, USA.*
e. *Phosio Corporation, Corvallis, OR, 97330, USA.*
f. *Department of Mechanical, Industrial, and Manufacturing Engineering, Oregon State University, Corvallis, OR, 97331, USA.*

Table of Contents

1. Instrumentation 3
2. Synthesis 6
3. Precipitation of Na-Nb_6 before CO_2 infusion. 9
4. Crystallographic information. 10
5. ^{133}Cs NMR Spectroscopy. 25
6. Small-angle X-ray Scattering of Redissolved Crystals. 29
7. ^{29}Si NMR Spectroscopy. 31
8. Characterization of precipitates, SEM/EDS 35
9. SAXS time-study 40
10. Raman and FTIR Spectroscopy; Gel characterization 41
11. ^{31}P NMR Spectroscopy. 45
12. Thermogravimetry-mass spectroscopy and CHN analysis. 52
13. Optical Spectroscopy 55
14. Electronic Properties 56

1. Instrumentation

X-ray Crystallography. Diffraction intensities for **$Nb_{16}Si_3$-CO_3** and **$Nb_{16}Ge_3$-CO_3**were collected at 223 K (**$Nb_{16}Si_3$-CO_3**) and 173 K (**$Nb_{16}Ge_3$-CO_3**) on a Rigaku SynergyS single crystal diffractometer using CuKα radiation (1.54184 Å) equipped with a PhotonJet-S Cu source (λ = 1.54178 Å) and a HyPix-6000HE photon counting detector. Space groups were determined based on systematic absences (**$Nb_{16}Si_3$-CO_3**) and intensity statistics (**$Nb_{16}Ge_3$-CO_3**). Absorption corrections were applied by SADABS.[1] Structures were solved by direct methods and Fourier techniques and refined on F^2 using full matrix least-squares procedures. All non-H atoms were refined with anisotropic thermal parameters. H atoms in both structures were not found and not taken into consideration. X-ray diffraction from these crystals is very weak at high angles. The diffraction data for both samples were collected only up to $2\theta_{max}$ = 102.028° (**$Nb_{16}Si_3$-CO_3**) and 101.864° (**$Nb_{16}Ge_3$-CO_3**). However, it provides the appropriate number of measured reflections per the number of the refined parameters: 16398/1740 (**$Nb_{16}Si_3$-CO_3**) and 17293/1860 (**$Nb_{16}Ge_3$-CO_3**). A relatively high value of R_{int} in **$Nb_{16}Si_3$-CO_3** is also related to very weak reflections at high angles. It was found that the crystal **$Nb_{16}Si_3$-CO_3** used for data collection was a twin consisting of two domains in a ratio of 82/18. The structure was determined and refined from HKLF4 reflections corresponding exclusively to the largest domain. The refinement of the structure based on the full reflection data, HKL5, did not yield an improved structure or goodness of fit, due to the combination of twinning and complex counterion and solvent-molecule disorder. In the structure, there are many disordered Cs and Na counterions, along with solvent water molecules, forming a complex network surrounding the Nb-POM. Some of the Cs atoms are disordered over two, three, and even four positions. Some positions of the Cs, Na, and O atoms are partially occupied. The Na atoms were assigned based on the refinement for the occupation factors and the difference between the Na-O and Cs-O contacts. In the residual density, several relatively high peaks are observed, mainly near the Cs atoms, indicating complex disorder of counterions and solvent water molecules. The RIGU option was used to refine both structures. All calculations were performed by the Bruker SHELXL-2014/7 package.[2]

Small-angle X-ray Scattering (SAXS). SAXS data were collected using an Anton-Parr SAXSess instrument with Cu-Kα radiation and line collimation. A 2-D image plate was used to collect data over the q range 0.018–2.5 $Å^{-1}$, with a sample-to-image plate distance of 26.1 cm. For **$Nb_{16}Si_3$-CO_3** and **$Nb_{16}Ge_3$-CO_3**, a 1, 2, 5, or 10 mM solution was prepared by dissolving the crystals in DI water, which was then syringe filtered (0.45 μm), loaded into a 1.5 mm glass capillary, and sealed with wax. For the time studies, the reaction solutions were prepared at normal concentration (1 M Cs-Nb_6) and diluted to 10 mM before analysis. Na_4SiO_4 was used as prepared for the CO_2 desiccator (~ 0.7 M Si). DI water was used as the solvent background, and scattering was measured for 30 minutes.

SAXSQUANT software was used for data collection and processing (normalization, primary beam removal, and background subtraction). The IRENA30 macros[3] within IGOR Pro 8.0.4.02 (WaveMetrics) software were used to plot and analyze the data. Simulated scattering curves were generated using SolX, utilizing structural files (.xyz) containing the entire structure or portions of it.

Thermogravimetric analysis–mass spectroscopy (TGA-MS). TGA analysis was done using a TA Instruments SDT Q600 and a Hien Gas Analyzer HPR-20 QIC EGA mass spectrometer. 20-50 mg of dried crystalline material or wet or dry gels was loaded into an alumina crucible and heated from room temperature to 900 °C at a rate of 10 °C/min under argon (100 mL/min). The chamber was flushed with argon gas for 2 hours prior to analysis to minimize background noise.

CHN Analysis. The carbon content was characterized using an NC2500 ThermoQuest Elemental Analyzer and an ECS 4010 CHNSO Analyzer, both fitted with a Costech Zero Blank Autosampler. For each sample, 3-6 mg was loaded into tin capsules and run in duplicate, with duplicate masses differing by no more than ±0.5 mg. The reported results are the average of the duplicate measurements of carbon content.

Raman Spectroscopy. Raman spectra were collected using a Thermo Scientific DXR spectrometer with a 780 nm laser source, a 400 lines per millimeter grating, a 50 μm slit, and 16 scans per spectrum.

Fourier-Transform Infrared Spectroscopy (FTIR). FTIR was collected on a PerkinElmer Spectrum Two FTIR Spectrometer from 400 to 4000 cm^{-1} using 16 scans per spectrum.

Solution-state Nuclear Magnetic Resonance Spectroscopy (NMR). Solution-state NMR was collected on a 500 MHz Bruker Ascend spectrometer equipped with a BBO probe. All samples were prepared using a solvent volume ratio of 90-10 H_2O-D_2O. Under normal crystallization conditions, the final concentration of Cs_4SiO_4 in the reaction solution is ~ 0.7 M. To mimic these conditions, the concentration was maintained at 0.7 M, but the reaction was scaled up to 600 μL (from 250 μL) to ensure an adequate volume for analysis. For ^{29}Si, the experiments were carried out at 25 °C using Bruker's zgig pulse program, modified to 1128 scans with a recycle delay of 30 seconds to allow all silicon resonances to relax fully. Data processing was performed with custom Python scripts using nmrPipe to apodize, zero-fill, Fourier-transform, and apply 5 Hz of line broadening to each data set. Baseline correction was performed using pybaseline with an improved asymmetrically reweighted penalized least squares Whittaker smoothing function. The ^{133}Cs experiments were collected over 128 scans and performed at 5, 10, 25, 35, 45, and 55 °C. The probe was cooled, and the sample temperature was allowed to equilibrate for 5 minutes before

analysis began. An external standard (0.1 M $CsNO_3$) was measured before each sample, and the axis was calibrated to 0 ppm.

Solid-state NMR Spectroscopy. Most solid-state NMR (ssNMR) spectroscopy experiments were performed with a Bruker widebore NMR spectrometer with B_0 = 9.4 T, equipped with an AVANCE III HD console. ^{133}Cs and ^{29}Si NMR experiments at 9.4 T utilized a 4 mm HXY Bruker MAS probe configured in double resonance HX mode. π/2 and π pulses were scaled from ^{13}C hard pulse calibration. ^{133}Cs π/2 and π pulse durations were 25 μs and 50 μs, respectively, corresponding to an RF field of 10 kHz. ^{29}Si π/2 and π pulse lengths were calibrated to be 6.0 and 12.0 μs, corresponding to an RF field of ca. 42 kHz. Additional ^{133}Cs ssNMR experiments at 14.1 T utilized a 600 MHz Bruker wide-bore magnet with a Bruker AVANCE NEO console. ^{133}Cs and ^{31}P NMR experiments used a 4 mm Bruker HXY probe in double mode. For ^{31}P, an RF field of ~51 kHz, corresponding to 4.9 and 9.8 μs pulse length, was used as the π/2 and π pulse. For the ^{1}H→^{31}P CP, ^{1}H and ^{31}P spin lock pulses during the CP step had optimized RF fields of 99 kHz and 80 kHz, respectively, with contact times of 5 ms and 14 ms. All NMR experiments were indirectly referenced to ^{1}H frequency of TMS ($\delta_{iso}(^{1}H)$ = 1.82 ppm) using the IUPAC relative NMR frequency.[4]

Spectroscopic Ellipsometry (SE). The gel thin films were prepared on an n-type silicon (100) substrate. The silicon wafer was cleaned in sequential acetone, isopropanol, and DI water baths, followed by oxidation of the substrate surface to SiO_2 by O_2 plasma ashing for 15 min at 50 W and 150 mTorr. On the substrate, 50 μL (Nb-Si, Nb-Ge) or 25 μL (Nb-PO4) of syringe-filtered (0.45 μm) precursor solution was spin-coated at 6000 rpm for 3 minutes. A JA Woolam spectroscopic ellipsometer was used to characterize the thickness and refractive index of the films. Ellipsometer measurements were taken over two angles (55° and 60°), while recording the change in psi and delta over the 350-1000 nm wavelength range. The data was fitted using CompleteEASE software against a Cauchy model (**equation 1**) using a Levenberg-Marquardt algorithm to perform a least squares fit. When the software could not automatically fit the data, the model's variable parameters (thickness, A, B, and C) were manually varied until a convergent model with a satisfactorily low MSE was produced.

$$n(\lambda) = A + \frac{B}{\lambda^2} + \frac{C}{\lambda^4} \qquad (1)$$

UV-visible spectroscopy (UV-vis). The thin films were prepared on glass slides (2” X 2”), and spin coating was done using the same procedure described above for SE measurements. Thin-film UV-vis measurements were performed on an Avantes UV-vis spectrometer equipped with an AvaLight-DH-S Deuterium-Halogen light source and a 3648-pixel CCD camera and processed using the Avasoft 8 software. Data were collected from 800–260 nm with an integration time of 2.5 milliseconds.

Impedance Spectroscopy. Dielectric and impedance data were measured on cylindrical monoliths of the Si and Ge gels prepared by slow evaporation. Phosphate gels were investigated, but their poor mechanical properties prevent them from being electroded. Monoliths were electroded using a silver epoxy (from MG Chemicals). The dielectric properties as a function of frequency were measured using an HP 4192 LF Impedance analyzer from 10 Hz to 1 MHz. The permittivity data were disregarded due to the high measured loss (tan(δ)). The impedance data were measured using a Solartron SI 1260 Impedance Gain-Phase Analyzer with a 1296 Dielectric Interface, needed for insulating samples. Data was collected from 1 Hz to 1 MHz at a 100 mV AC bias and an integration time of 1 second. These data were fit using an equivalent circuit model containing parallel resistive and capacitive (constant phase) elements and a Warburg element in series. The fitting was performed using the ZView4 software package. The resistivity of the gel was calculated using:

$$\rho = R\left(\frac{A}{\ell}\right) \tag{2}$$

where ρ is the resistivity (Ω·cm), R is the measured resistance (Ω), A is the area of the monolith (cm^2), and ℓ is the length of the monolith (cm).

From the resistivity, the conductivity was calculated using:

$$\sigma = \frac{1}{\rho} \tag{3}$$

where ρ is the resistivity (Ω·cm).

2. Synthesis

Synthesis of cesium hexaniobate, $Cs_8[Nb_6O_{19}]\cdot 14H_2O$ (Cs-Nb_6): $CsNb_6$ was synthesized following a previously reported procedure.[5] Briefly, $Nb_2O_5\cdot 3\ H_2O$ (20.6115 g, ~129 mmol) is added in small aliquots to a 90 ˚C solution of 50 wt% CsOH in water (34 mL, 5.7 M). Each subsequent aliquot of Nb_2O_5 is added once the solution becomes clear, indicating complete dissolution of the previous aliquot. Dissolution is slow initially but speeds up for later aliquots. The resulting solution is gravity-filtered, and the solid product is isolated by precipitation with excess isopropanol (IPA, 600 mL) under constant stirring. The resulting white powder is vacuum filtered, washed with additional IPA, and dried in a vacuum oven overnight. Product mass = 41.9784 g, formulated $Cs_8Nb_6O_{19}\cdot 14\ H_2O$, yield = 89.8 % based on Nb.

Synthesis of cesium silicate solution, Cs_4SiO_4 (aq): 5 mL of CsOH (50 wt%, 5.7 M, 28.5 mmol Cs) is placed in a vial in a preheated sand bath (90˚C). Under constant stirring, fumed silica (SiO_2, 0.43 g, 7.125 mmol) is added to the warm solution and stirred until completely

dissolved (~30 minutes). The equivalency ratio is four cesium to one silicon (~1.4 M Cs_4SiO_4).

Synthesis of sodium germanate solution, Na_4GeO_4 (aq): 2 mL of NaOH (4 M, 8 mmol Na) is placed in a vial in a preheated sand bath (90˚C). Under constant stirring, germanium oxide (GeO_2, 0.209 g, 2 mmol) is added to the warm solution and stirred until completely dissolved (~45 minutes). The equivalency ratio is four sodium to one germanium (~1 M Na_4GeO_4).

Synthesis of cesium phosphate, tribasic, Cs_3PO_4: 5 mL of CsOH (50 wt%, 5.7 M, 28.5 mmol Cs) is placed in a vial, followed by the slow, dropwise addition of concentrated H_3PO_4 (14.6 M, 0.65 mL, 9.41 mmol PO_4^{3-}) because the neutralization reaction is highly exothermic. The equivalency ratio is three cesium to one phosphate (~1.7 M Cs_3PO_4).

Synthesis of $Nb_{16}Si_3$-CO_3: Crystalline $Nb_{16}Si_3$-CO_3 is synthesized by adding $CsNb_6$ (0.55 g, 1.5 mmol Nb, ~1 M Nb_6) to 125 µL of DI H_2O in a 4 mL vial, followed by the addition of 125 µL of 1.4 M Cs_4SiO_4 (0.72 M SiO_4^{4-}, 0.18 mmol Si). This gives a ratio of 8.3 Nb per 1 Si. The solution is stirred briefly, then placed uncapped in a desiccator prefilled with dry ice (2.4-2.8 lbs) and exposed to CO_2 for approximately 18 hours (overnight). Small, colorless, hexagonal $Nb_{16}Si_3$-CO_3 crystals begin appearing within 24 hours and are fully formulated as $Cs_{24}[Nb_7O_{22}(NbO(CO_3)_2)_9(Si_3O_9)]\cdot 19.6\ H_2O$ (yield = 80.4 % based on Nb).

Synthesis of $Nb_{16}Ge_3$-CO_3: Crystalline $Nb_{16}Ge_3$-CO_3 is synthesized by adding $CsNb_6$ (0.55 g, 1.5 mmol Nb, ~1 M Nb_6) to 150 µL of DI H_2O in a 4 mL vial, followed by the addition of 100 µL of 1 M Na_4GeO_4 (0.4 M GeO_4^{4-}, 0.10 mmol Ge). This gives a ratio of 15 Nb per 1 Ge. Upon adding the Na_4GeO_4 solution, a white precipitate forms, which we hypothesize is the much less soluble sodium hexaniobate cluster ($NaNb_6$). The solution is stirred briefly, then placed uncapped in a desiccator prefilled with dry ice and exposed to CO_2 for approximately 18 hours (overnight). After 18 hours, the initial precipitate redissolves, leaving the solution clear (**figure S1**). Colorless, rectangular crystals of $Nb_{16}Ge_3$-CO_3 begin to appear within 24-48 hours. The crystals are unstable and must be collected or analyzed within 48 hours of forming, or they tend to redissolve. $Nb_{16}Ge_3$-CO_3 is fully formulated as $Cs_{21}Na_3[Nb_7O_{22}(NbO(CO_3)_2)_9(Ge_3O_9)]\cdot 33.6\ H_2O$ (yield = 41.6 % based on Nb).

Synthesis of Nb-Si gel: Cs-Nb_6 (0.55 g, 1.5 mmol Nb) is dissolved in 250 µL of DI H_2O (~1 M Nb_6) in a 4 mL vial. The solution is heated for approximately 20 min on a 75 ˚C hot plate with constant stirring. Solid fumed silica (SiO_2, 35 mg, 0.58 mmol Si) is added to the warm Cs-Nb_6 solution and stirred until fully dissolved (~10 minutes). This gives a ratio of approximately 3 Nb per 1 Si. The solution is then placed uncapped in a desiccator prefilled with dry ice and exposed to CO_2 for approximately 18 hours (overnight). After 18 hours, a

clear viscous solution remains. The vial is covered with parafilm, perforated with a needle, and left to dry on the benchtop. A translucent, wet gel forms within 24 to 48 hours on the benchtop. Upon further drying (~ 1 month), a translucent, solid monolith/disk forms. For ssNMR, the gel is dried in a 60 ˚C oven overnight, at which point the gel/solid turns opaque.

Synthesis of Nb-Ge gel: Cs-Nb_6 (0.55 g, 1.5 mmol Nb) is dissolved in 250 µL of DI H_2O (~1 M Nb_6) in a 4 mL vial. The solution is heated for approximately 20 min on a 75 ˚C hot plate with constant stirring. Solid germanium oxide (GeO_2, 12 mg, 0.11 mmol Ge) is added to the warm Cs-Nb_6 solution and stirred until fully dissolved (~20 minutes). This gives a ratio of approximately 13 Nb per 1 Ge. The solution is then placed uncapped in a desiccator prefilled with dry ice and exposed to CO_2 for approximately 18 hours (overnight). After 18 hours, a clear viscous solution remains. The vial is covered with parafilm, perforated with a needle, and left to dry on the benchtop. A translucent, wet gel forms within 24 to 48 hours on the benchtop. Upon further drying (~ 1 month), a translucent, solid monolith/disk forms.

Synthesis of Nb-PO_4 gel: Cs-Nb_6 (0.55 g, 1.5 mmol Nb) is dissolved in 100 µL of DI H_2O (~1 M Nb_6) in a 4 mL vial, followed by the addition of 150 µL of 1.7 M Cs_3PO_4 (~ 1 M PO_4^{3-}, 0.25 mmol P). This gives a ratio of 6 Nb per 1 P. The solution is then placed uncapped in a desiccator prefilled with dry ice and exposed to CO_2 for approximately 18 hours (overnight). After 18 hours, a clear viscous solution remains. The vial is covered with parafilm, perforated with a needle, and left to dry on the benchtop. A translucent, wet gel forms within 24 to 48 hours on the benchtop. Upon further drying (~1 month), a translucent, solid monolith/disk forms. For ssNMR, the gel is dried in a 60 ˚C oven overnight, at which point the gel/solid turns opaque.

Thin-film preparation: The Nb-Si, Nb-Ge, and Nb-PO_4 all form extremely viscous solutions after 18 hours of CO_2. Due to their viscosity, these samples could not be spin-coated for thin film measurements. However, the samples can be diluted by 50% before CO_2 exposure and still form gels, although at longer time intervals (~5 days). Therefore, the thin film samples were prepared as described above. Then, an additional 250 µL of DI water was added, and the samples were placed in the desiccator.

3. Precipitation of Na-Nb_6 before CO_2 infusion.

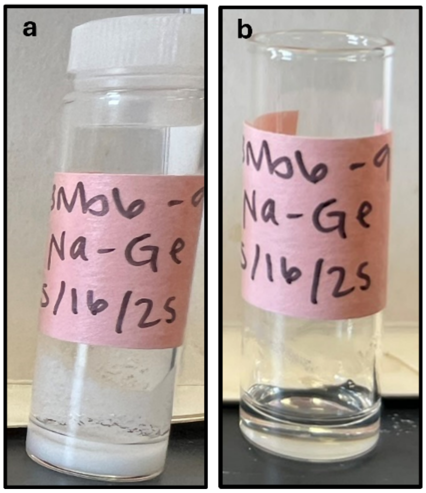

Figure S1. (a) precipitation of Na-Nb_6 when Na_4GeO_4 is added, and (b) after CO_2 exposure the precipitate redissolves and the new Nb-POM that forms (**$Nb_{16}Ge_3$-CO_3**) is more soluble than Nb_6 alone.

4. Crystallographic information.

Table S1. Crystallographic information for **$Nb_{16}Si_3$-CO_3**.

Identification	$Nb_{16}Si_3$-CO_3
CSD number	2494764
Empirical formula	$C_{18}Cs_{24}Nb_{16}O_{113.6}Si_3H_{39.2}$
Moiety formula	$Cs_{24}[Nb_7O_{22}[NbO(CO_3)_2]_9(Si_3O_9)]\cdot 19.6\ H_2O$
Formula weight	6833.51
Temperature/K	223(2)
Crystal system	monoclinic
Space group	C2/c
a/Å	28.8284(4)
b/Å	16.3406(2)
c/Å	65.4933(7)
α/°	90
β/°	92.4720(10)
γ/°	90
Volume/$Å^3$	30823.4(7)
Z	8
ρ_{calc} g/cm^3	2.945
μ/mm^{-1}	54.034
F(000)	24590
Crystal size/mm^3	0.16 × 0.01 × 0.005
Radiation	CuKα (λ = 1.54184)
2Θ range for data collection/°	5.402 to 102.028
Index ranges	$-25 \leq h \leq 28$, $-16 \leq k \leq 16$, $-63 \leq l \leq 65$
Reflections collected	100056
Independent reflections	16398 [R_{int} = 0.2324, R_{sigma} = 0.1091]
Data/restraints/parameters	16398/6615/1740
Goodness-of-fit on F^2	1.337
Final R indexes [I>=2σ (I)]	R_1 = 0.1178, wR_2 = 0.3254
Final R indexes [all data]	R_1 = 0.1292, wR_2 = 0.3366
Largest diff. peak/hole / e $Å^{-3}$	3.55/-3.38

Table S2. Crystallographic information for **$Nb_{16}Ge_3$-CO_3**.

Identification	$Nb_{16}Ge_3$-CO_3
CSD number	2494765
Empirical formula	$O_{127.56}Ge_3Nb_{16}C_{18}Cs_{21}Na_3H_{67.12}$
Moiety formula	$Cs_{21}Na_3[Nb_7O_{22}[NbO(CO_3)_2]_9(Ge_3O_9)]\cdot33.6\ H_2O$
Formula weight	6889.12
Temperature/K	173(2)
Crystal system	triclinic
Space group	P-1
a/Å	15.6974(2)
b/Å	22.9741(4)
c/Å	23.8925(3)
α/°	107.0550(10)
β/°	93.7840(10)
γ/°	93.1270(10)
Volume/Å^3	8194.9(2)
Z	2
ρ_{calc} g/cm^3	2.792
μ/mm^{-1}	46.254
F(000)	6271
Crystal size/mm^3	0.1 × 0.05 × 0.01
Radiation	CuKα (λ = 1.54184)
2Θ range for data collection/°	5.66 to 101.864
Index ranges	-15 ≤ h ≤ 15, -23 ≤ k ≤ 22, -23 ≤ l ≤ 23
Reflections collected	57340
Independent reflections	17293 [R_{int} = 0.0922, R_{sigma} = 0.0728]
Data/restraints/parameters	17293/6447/1860
Goodness-of-fit on F^2	1.033
Final R indexes [I>=2σ (I)]	R_1 = 0.0963, wR_2 = 0.2859
Final R indexes [all data]	R_1 = 0.1108, wR_2 = 0.3017
Largest diff. peak/hole / e Å^{-3}	2.53/-2.61

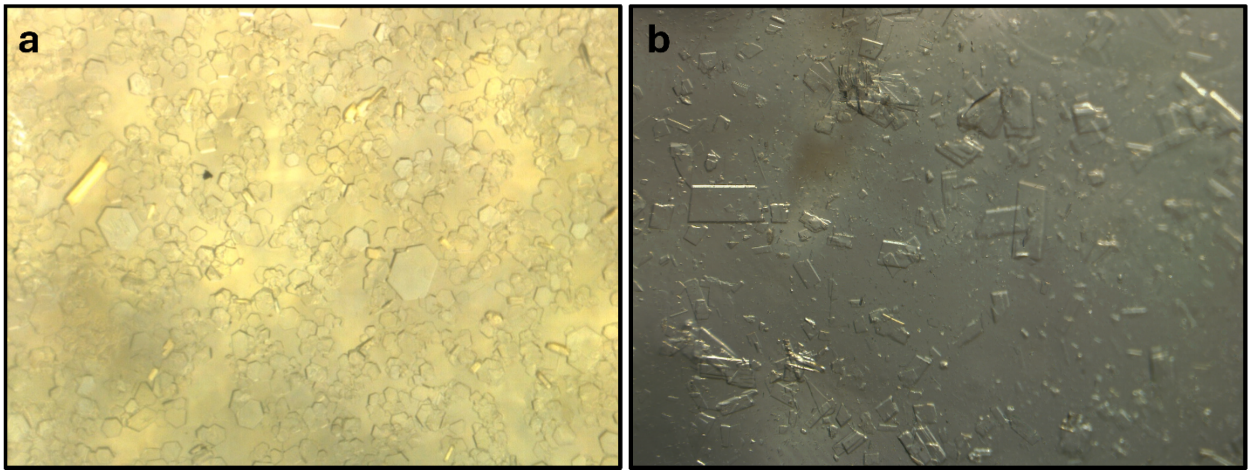

Figure S2. Microscope images of (a) **$Nb_{16}Si_3$-CO_3** and (b) **$Nb_{16}Ge_3$-CO_3**, showing their different morphologies.

Table S3. Bond valences (BVS) of **$Nb_{16}Si_3$-CO_3** with the coordination number and atom site.

Atom	BVS	coordination number	site description
NB1	4.95	6	Nb_7 unit connected to hexaniobate and chains
NB2	5.07	6	hexaniobate Nb-atom connected to chains
NB3	5.08	6	hexaniobate Nb-atom connected to chains
NB4	5.00	6	hexaniobate Nb-atom connected to chains
NB5	4.96	7	Nb-atom within the chains/tentacles (part of Nb_{10} plane)
NB6	5.24	7	Nb-atom within the chains/tentacles (part of Nb_{10} plane)
NB7	5.01	7	Nb-atom within the chains/tentacles (part of Nb_{10} plane)
NB8	4.86	6	hexaniobate Nb-atom with a terminal 'yl' oxo
NB9	5.02	7	Nb-atom within the chains/tentacles
NB10	4.93	6	hexaniobate Nb-atom with a terminal 'yl' oxo
NB11	5.15	7	Nb-atom within the chains/tentacles
NB12	5.31	7	Nb-atom within the chains/tentacles
NB13	4.98	6	hexaniobate Nb-atom with a terminal 'yl' oxo
NB14	5.20	7	Nb-atom within the chains/tentacles
NB15	5.03	7	Nb-atom within the chains/tentacles
NB16	4.95	7	Nb-atom within the chains/tentacles
SI1	4.08	4	part of Si_3O_9 cyclic trimer
SI2	4.01	4	
SI3	4.18	4	
O10	2.01		μ_2 bridge connecting chains/tentacles to Si cyclic trimer (Nb-O-Si)
O15	2.04		
O16	2.03		

O17	2.00		
O30	1.95		
O35	1.86		
O19	1.94		chelating O-atom from carbonate (Nb-O-C)
O20	2.18		
O22	2.29		
O24	1.94		
O25	1.76		
O27	2.22		
O28	1.80		
O29	2.16		
O32	1.68		
O33	1.96		
O38	1.67		
O40	2.29		
O42	2.06		
O43	1.85		
O44	1.87		
O45	1.79		
O48	1.99		
O49	2.11		
O50	1.91		
O51	1.82		
O53	1.79		
O54	1.82		
O55	1.74		
O56	2.14		
O58	1.90		
O61	2.11		
O62	2.41		
O69	1.82		
O71	1.90		
O74	2.01		
O75	2.27		
O76	2.07		
O78	1.51		
O80	2.14		

O82	1.97		
O87	2.13		
O7	1.99		
O9	2.03		μ_2 bridging O atom (Si-O-Si)
O12	1.94		
O2	1.91		μ_2 Nb-O-Nb bridge connecting Nb_7 unit to hexaniobate
O3	1.90		μ_2 Nb-O-Nb bridge connecting Nb_7 unit to Nb in chains (Nb_{10} plane)
O4	1.87		μ_2 Nb-O-Nb bridge connecting Nb_7 unit to Nb in chains (Nb_{10} plane)
O5	1.96		μ_2 Nb-O-Nb bridge in chain/tentacle
O6	1.90		Nb-O-Nb bridge connecting Nb_7 unit to Nb in chains (Nb_{10} plane)
O8	1.65		μ_2 Nb-O-Nb bridge in hexaniobate
O11	1.89		Nb-O-Nb bridge connecting Nb_7 unit to hexaniobate
O13	1.94		Nb-O-Nb bridge connecting Nb_7 unit to hexaniobate
O14	1.81		μ_2 Nb-O-Nb bridge in hexaniobate
O18	1.81		μ_2 Nb-O-Nb bridge in hexaniobate
O21	1.84		μ_2 Nb-O-Nb bridge in hexaniobate
O23	1.77		μ_2 Nb-O-Nb bridge in hexaniobate
O26	1.85		μ_2 Nb-O-Nb bridge in hexaniobate
O31	1.70		μ_2 Nb-O-Nb bridge in hexaniobate
O34	1.80		μ_2 Nb-O-Nb bridge in hexaniobate
O36	1.82		Terminal Nb=O in hexaniobate
O37	1.56		Terminal Nb=O in hexaniobate
O39	1.92		μ_2 Nb-O-Nb bridge connecting Nb of hexaniobate to Nb of chain/tentacle (w/in the Nb_{10} plane)
O41	1.77		μ_6 O-atom in center of hexaniobate
O46	1.85		μ_2 Nb-O-Nb bridge in chain/tentacle
O47	1.90		μ_2 Nb-O-Nb bridge in hexaniobate
O52	1.82		μ_2 Nb-O-Nb bridge connecting Nb of hexaniobate to Nb of chain/tentacle (w/in the Nb_{10} plane)
O59	1.84		Terminal Nb=O at the end of chain/tentacle
O60	1.73		Terminal Nb=O in hexaniobate
O63	1.80		μ_2 Nb-O-Nb bridge in chain/tentacle

O64	1.97		μ_2 Nb-O-Nb bridge in chain/tentacle
O65	1.59		Terminal Nb=O at the end of chain/tentacle
O68	1.72		Terminal Nb=O at the end of chain/tentacle
O72	1.92		μ_2 Nb-O-Nb bridge connecting Nb of hexaniobate to Nb of chain/tentacle (w/in the Nb_{10} plane)
O73	1.87		μ_2 Nb-O-Nb bridge in chain/tentacle
O77	1.92		μ_2 Nb-O-Nb bridge in chain/tentacle
O67	1.99		O-atom of C=O carbonate
O79	1.76		
O81	1.85		
O83	1.81		
O85	2.02		
O86	1.97		
O88	1.84		
O90	1.57		
O91	2.06		
O94	1.77		
O97	1.60		
O99	1.60		
O100	1.83		
O103	1.63		
O107	1.54		
O108	1.74		
O109	1.65		
O110	1.59		

Table S4. Bond valences (BVS) of **$Nb_{16}Ge_3$-CO_3** with the coordination number and atom site.

Atom	BVS	coordination number	site description
Nb1	4.84	6	hexaniobate Nb-atom with a terminal 'yl' oxo
Nb2	5.16	6	hexaniobate Nb-atom with a terminal 'yl' oxo
Nb3	5.01	6	hexaniobate Nb-atom with a terminal 'yl' oxo
Nb4	4.79	6	hexaniobate Nb-atom connected to chains
Nb5	4.83	7	hexaniobate Nb-atom connected to chains
Nb6	4.87	7	hexaniobate Nb-atom connected to chains
Nb7	5.03	7	Nb_7 unit connected to hexaniobate and chains
Nb8	4.99	6	Nb-atom within the chains/tentacles (part of Nb_{10} plane)

Nb9	5.20	7	Nb-atom within the chains/tentacles (part of Nb_{10} plane)
Nb10	5.10	6	Nb-atom within the chains/tentacles (part of Nb_{10} plane)
Nb11	5.06	7	Nb-atom within the chains/tentacles
Nb12	5.05	7	Nb-atom within the chains/tentacles
Nb13	5.13	6	Nb-atom within the chains/tentacles
Nb14	5.14	7	Nb-atom within the chains/tentacles
Nb15	4.99	7	Nb-atom within the chains/tentacles
Nb16	5.35	7	Nb-atom within the chains/tentacles
Ge1	4.38	4	part of Ge_3O_9 cyclic trimer
Ge2	4.19	4	
Ge3	3.98	4	
O9	2.09		μ_2 bridge connecting chains/tentacles to Ge cyclic trimer (Nb-O-Ge)
O10	1.86		
O14	1.91		
O18	2.00		
O43	1.97		
O86	1.92		
O7	1.90		chelating O-atom from carbonate (Nb-O-C)
O8	1.87		
O11	1.83		
O16	1.88		
O17	1.86		
O19	1.86		
O20	1.98		
O23	1.71		
O24	1.82		
O27	2.01		
O29	1.91		
O32	2.08		
O44	1.71		
O45	1.79		
O55	1.68		
O57	2.00		
O58	1.77		

O59	1.83		
O60	2.23		
O64	2.01		
O65	2.09		
O68	1.85		
O69	1.86		
O70	2.36		
O71	1.88		
O72	1.79		
O74	1.88		
O76	2.00		
O77	2.00		
O79	1.93		
O89	1.93		
O91	1.89		
O94	1.81		
O99	2.12		
O100	1.98		
O106	2.15		
O12	2.00		μ_2 bridging O atom (Ge-O-Ge)
O13	2.07		
O15	1.95		
O1	1.86		Nb-O-Nb bridge connecting Nb_7 to Nb in chains (Nb_{10})
O3	1.89		Nb-O-Nb bridge connecting Nb_7 to Nb in chains (Nb_{10})
O5	1.80		Nb-O-Nb bridge connecting Nb_7 to hexaniobate
O6	1.84		Nb-O-Nb bridge connecting Nb_7 to Nb in chains (Nb_{10})
O22	1.75		Terminal Nb=O at the end of chain/tentacle
O25	1.73		μ_6 O-atom in center of hexaniobate
O26	1.67		Nb-O-Nb bridge in hexaniobate
O31	1.72		Nb-O-Nb bridge in hexaniobate
O40	1.72		Terminal Nb=O at the end of chain/tentacle
O46	1.84		Nb-O-Nb bridge in chain/tentacle
O48	1.90		Nb-O-Nb bridge connecting Nb_7 to hexaniobate
O49	1.77		Nb-O-Nb bridge in hexaniobate
O51	1.83		μ_2 Nb-O-Nb bridge connecting Nb of hexaniobate to Nb of chain/tentacle (w/in the Nb_{10} plane)
O52	1.78		Nb-O-Nb bridge in hexaniobate
O54	1.98		Nb-O-Nb bridge in chain/tentacle

O56	1.45		Terminal Nb=O in hexaniobate
O61	1.80		Nb-O-Nb bridge in hexaniobate
O62	1.77		Nb-O-Nb bridge in hexaniobate
O66	2.03		Nb-O-Nb bridge in chain/tentacle
O78	1.55		Terminal Nb=O in hexaniobate
O80	1.93		Nb-O-Nb bridge in chain/tentacle
O83	1.71		Nb-O-Nb bridge in hexaniobate
O84	1.74		Nb-O-Nb bridge in hexaniobate
O85	1.47		Terminal Nb=O in hexaniobate
O88	1.84		μ_2 Nb-O-Nb bridge connecting Nb of hexaniobate to Nb of chain/tentacle (w/in the Nb_{10} plane)
O90	1.88		Nb-O-Nb bridge connecting Nb_7 to hexaniobate
O92	1.90		Nb-O-Nb bridge in chain/tentacle
O95	1.75		μ_2 Nb-O-Nb bridge connecting Nb of hexaniobate to Nb of chain/tentacle (w/in the Nb_{10} plane)
O96	1.53		Terminal Nb=O at the end of chain/tentacle
O97	1.76		Nb-O-Nb bridge in hexaniobate
O102	1.97		Nb-O-Nb bridge in chain/tentacle
O28	1.60		O-atom of C=O carbonate
O30	1.68		
O33	1.85		
O36	1.80		
O37	1.68		
O38	1.38		
O39	1.78		
O41	1.59		
O42	1.85		
O50	1.65		
O53	1.67		
O73	1.88		
O75	1.68		
O87	1.92		
O93	1.87		
O98	2.02		
O104	1.88		
O107	1.90		

Table S5. Comparison of the Si-O and Ge-O bond distances in the $\mathbf{Nb_{16}Si_3\text{-}CO_3}$ **and** $\mathbf{Nb_{16}Ge_3\text{-}CO_3}$ phases to the previously reported $Cs_6Si_3O_9$ and $Cs_6Ge_3O_9$ phases.[5,6] The Si-O and Ge-O bond distances for $\mathbf{Nb_{16}Si_3\text{-}CO_3}$ **and** $\mathbf{Nb_{16}Ge_3\text{-}CO_3}$ fall within the error of the literature values.

Sample	Atom 1	Atom 2	Distance (1,2) Å	Average distance (Å)
$\mathbf{Nb_{16}Si_3\text{-}CO_3}$	Si3	O15	1.583	1.62 ± 0.0178
	Si1	O17	1.599	
	Si2	O9	1.600	
	Si3	O30	1.607	
	Si3	O7	1.609	
	Si1	O16	1.612	
	Si2	O10	1.613	
	Si1	O12	1.622	
	Si3	O9	1.629	
	Si1	O7	1.636	
	Si2	O35	1.638	
	Si2	O12	1.641	
$Cs_6Si_3O_9$	Si3	O8	1.584	1.63 ± 0.0405
	Si1	O5	1.595	
	Si2	O7	1.596	
	Si3	O9	1.597	
	Si2	O6	1.599	
	Si1	O4	1.604	
	Si3	O2	1.661	
	Si2	O1	1.668	
	Si1	O1	1.674	
	Si3	O3	1.675	
	Si1	O3	1.674	
	Si2	O2	1.682	
$\mathbf{Nb_{16}Ge_3\text{-}CO_3}$	Ge1	O9	1.673	1.73 ± 0.0313
	Ge1	O18	1.693	
	Ge2	O43	1.697	
	Ge2	O13	1.720	
	Ge1	O12	1.727	
	Ge3	O10	1.735	
	Ge3	O86	1.745	
	Ge2	O15	1.749	
	Ge3	O13	1.753	
	Ge1	O15	1.764	
	Ge2	O14	1.765	

	Ge3	O12	1.769	
$Cs_6Ge_3O_9$	Ge3	O8	1.716	1.76 ± 0.0422
	Ge1	O4	1.718	
	Ge1	O5	1.724	
	Ge2	O7	1.725	
	Ge2	O6	1.725	
	Ge3	O9	1.731	
	Ge2	O1	1.791	
	Ge2	O2	1.792	
	Ge1	O3	1.798	
	Ge3	O3	1.802	
	Ge1	O1	1.813	
	Ge3	O2	1.818	

Table S6. Bond angles of the Nb-O-Nb in the tentacles that are bridged to the silicate or germanate trimer (**figure 1e**). The Nb-O-Nb bond angle is larger, on average, for **$Nb_{16}Ge_3$-CO_3** due to germanate's (Ge(IV)) larger ionic radius (0.39 Å versus 0.26 Å for Si(IV)).

Sample	Atoms (1, 2, 3)	Angle (°)
**$Nb_{16}Si_3$-CO_3**	Nb16, O63, Nb15	142.8
	Nb14, O73, Nb9	146.1
	Nb12, O46, Nb11	145.6
	average bond angle (°)	144.8
**$Nb_{16}Ge_3$-CO_3**	Nb0K, O01Q, Nb0M	149.5
	Nb0B, O01X, Nb0R	151.9
	Nb0G, O02U, Nb0H	149.5
	average bond angle (°)	150.3

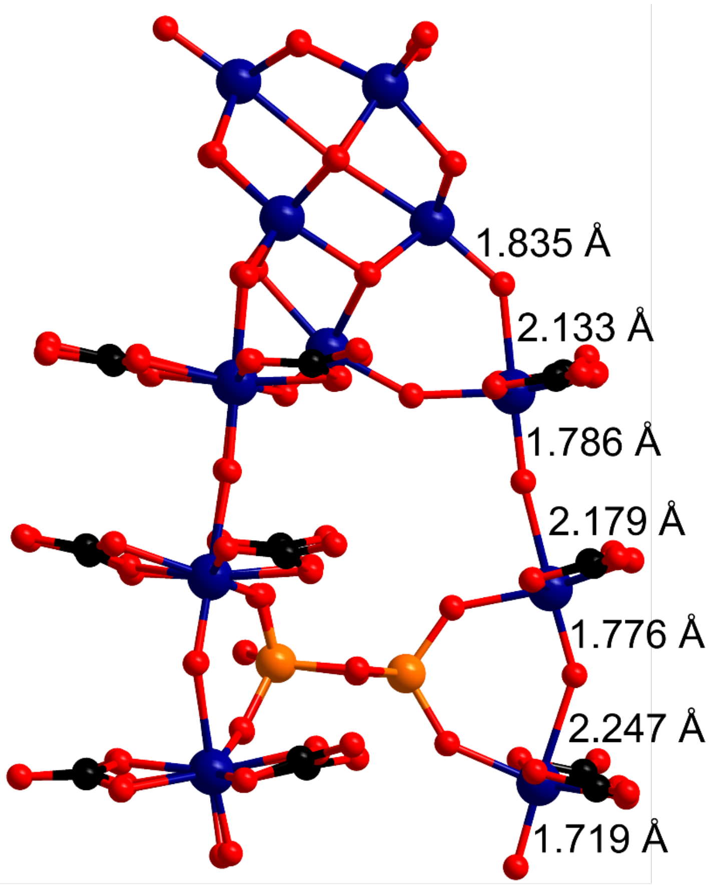

Figure S3. Preservation of the Jahn-Teller distortion down the tentacles of the jellyfish.

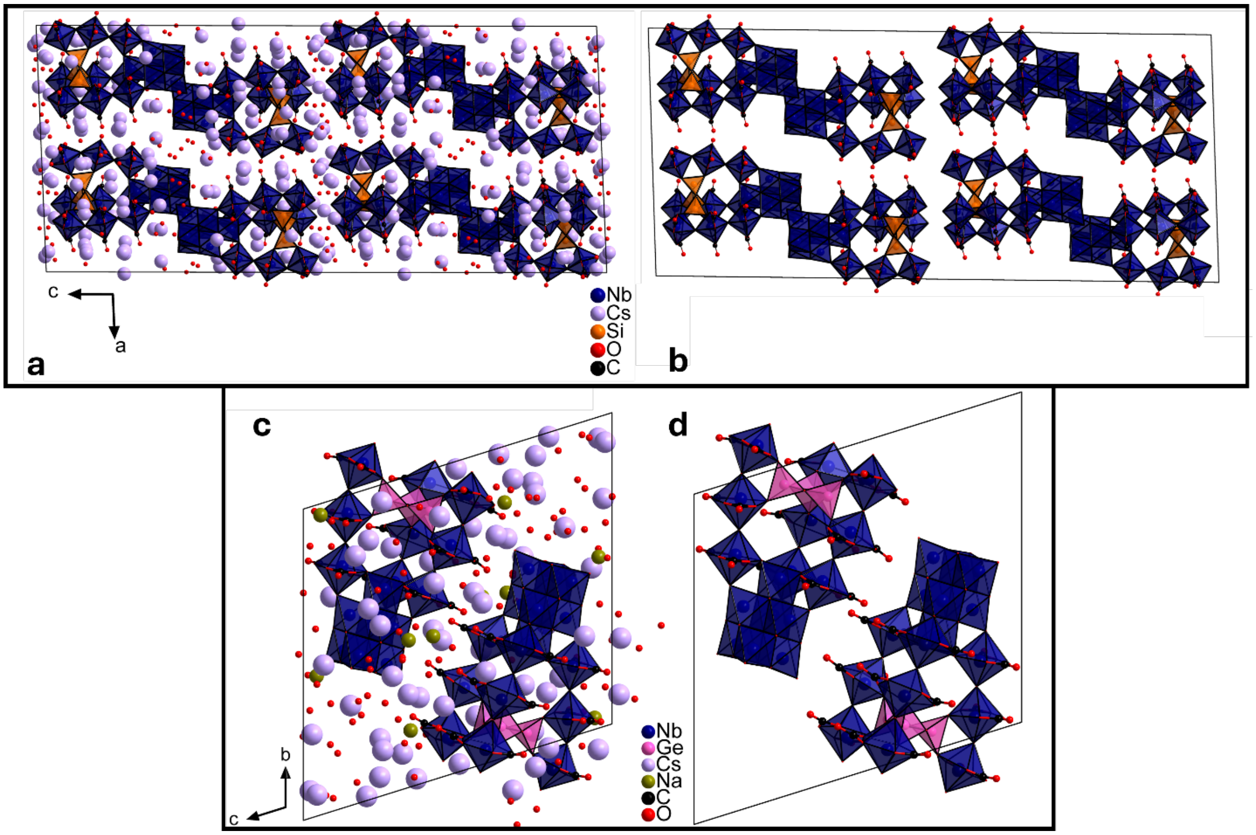

Figure S4. (a) and (b) the unit cell packing arrangement of **$Nb_{16}Si_3$-CO_3**, viewed along the b-axis, with (a) depicting the countercations (Cs^+) and lattice water (H-atoms omitted for clarity). (c) and (d) the unit cell packing of **$Nb_{16}Ge_3$-CO_3** viewed along the *a*-axis with (c) showing the countercations (Cs^+, Na^+) and lattice water (H-atoms omitted for clarity).

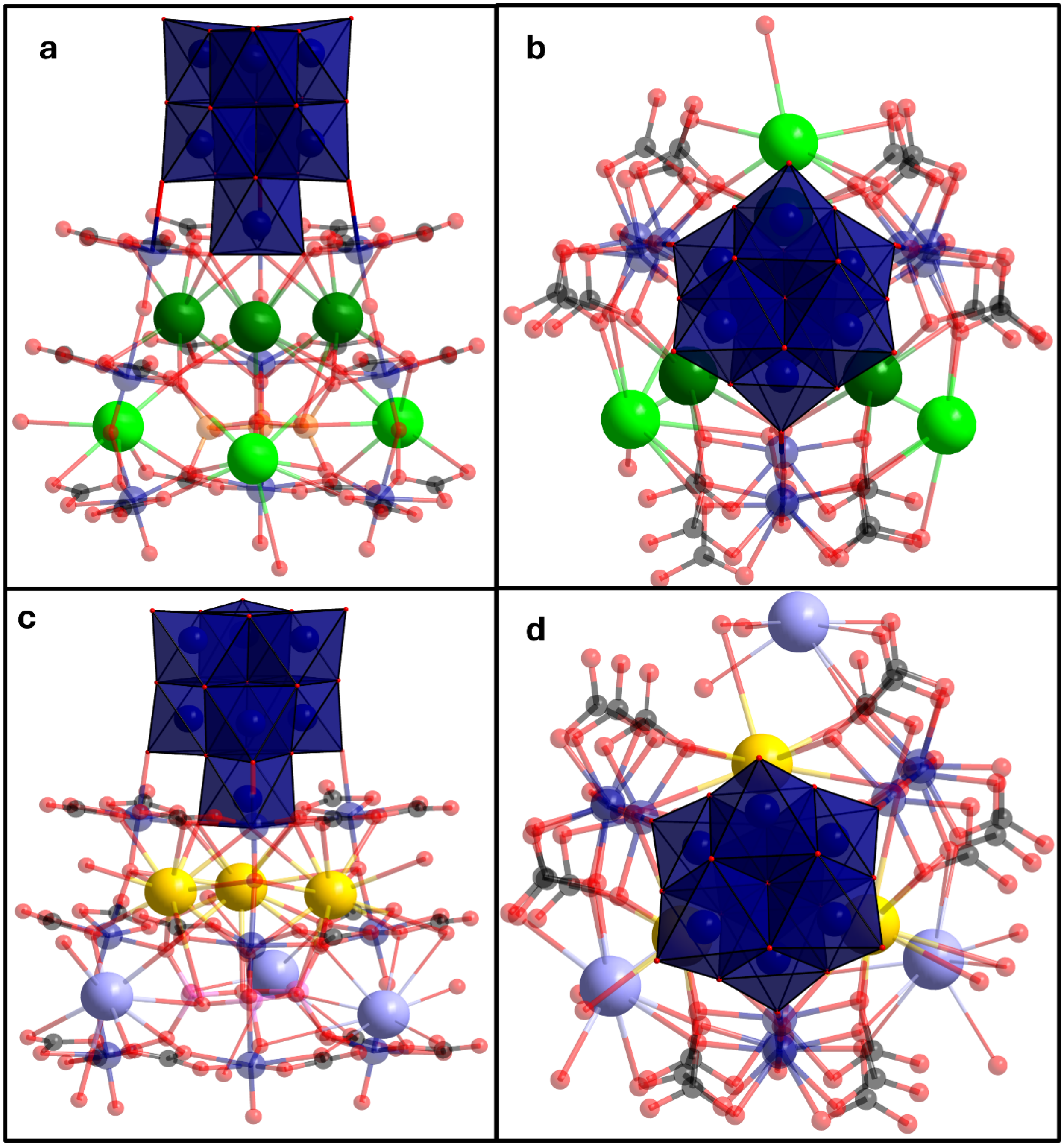

Figure S5. The crown-like arrangement of Cs^+ countercations, tucked up within the tentacles of the jellyfish structures for (a, b) **$Nb_{16}Si_3$-CO_3** (Cs atoms in dark and light green) and (c, d) **$Nb_{16}Ge_3$-CO_3** (Cs atoms in yellow and light purple). The Cs environment in both structures is similar, showing its importance for crystallization.

Table S7. Detailed Cs-O bond lengths and Cs coordination number for **$Nb_{16}Si_3$-CO_3** and **$Nb_{16}Ge_3$-CO_3**, showing the similarities between the Cs bonding environments.

Sample	Atom 1	Atom 2	average bond length (Å)	Cs coordination number
$Nb_{16}Si_3$-CO_3	Cs1	O43	3.33	11
	Cs2	O38	3.30	11
	Cs3	O33	3.29	11
	Cs14	O78	3.56	9
	Cs22	O123	3.42	10
	Cs24	O61	3.51	8
			avg. bond length	avg coord. number
			3.4	10.0
$Nb_{16}Ge_3$-CO_3	Cs1	O71	3.39	12
	Cs2	O16	3.28	12
	Cs3	O8	3.38	13
	Cs10	O91	3.29	9
	Cs14	O67	3.29	9
	Cs16	O203	3.34	9
			avg. bond length	avg coord. number
			3.3	10.7

5. ^{133}Cs NMR Spectroscopy.

Table S8. Summary of the ^{133}Cs ssNMR experimental parameters.

Figure	Compound	B_0 (T)	MAS (kHz)	Recycle Delay (s)	# of scans	Exp. Time (hr)
Figure 3b	$Nb_{16}Si_3$-CO_3	9.4	10	1.3	1670	0.6
Figure 3b	Nb-Si gel	9.4	10	1.3	10240	3.7
Figure S6	$Nb_{16}Si_3$-CO_3	14.1	10	20	512	2.8
Figure S7	Nb-PO_4 gel	14.1	10	40[a]	512	5.7
Figure S7	Nb-PO_4 dry gel	14.1	10	60[b]	512	8.5

[a]*The maximum recycle delay was 40 s. A series of NMR experiment were acquired under the same conditions using a recycle delay of 40 s, 10 s, and 0.1 s.*
[b]*The maximum recycle delay was 40 s. A series of NMR experiment were acquired under the same conditions using a recycle delay of 60 s, 10 s, and 0.1 s.*

Table S9. Solution ^{133}Cs NMR summary of peak positions and full width at half maximum (FWHM).

Sample	peak position (ppm) [FWHM, Hz]	peak position (ppm) [FWHM, Hz]
0.1 M $CsNO_3$	0 [2.172]	
0.1 M $Cs_8Nb_6O_{19}$	21.2766 [3.743]	
Cs_4SiO_4 (pH 14)	24.0420 [1.416]	
Cs_4GeO_4 (pH 14)	36.7977 [5.342]	
Cs-germanate (17 Cs: 1 Ge)	34.1403 [5.455]	
$Nb_{16}Si_3$-CO_3 (0.1 M, 5°C)	21.6904 [55.723]	50.9418 [423.392]
$Nb_{16}Si_3$-CO_3 (0.1 M, 10°C)	21.2728 [53.803]	51.4257 [432.243]
$Nb_{16}Si_3$-CO_3 (0.1 M, 25°C)	20.4897 [57.354]	51.20 [451.552]
$Nb_{16}Si_3$-CO_3 (0.1 M, 35°C)	19.3213 [59.213]	
$Nb_{16}Si_3$-CO_3 (0.1 M, 45°C)	18.5772 [65.611]	
$Nb_{16}Si_3$-CO_3 (0.1 M, 55°C)	17.9106 [73.511]	
$Nb_{16}Si_3$-CO_3 (0.1 M, 25°C after heating)	20.2433 [61.50]	53 [644.430]
$Nb_{16}Ge_3$-CO_3 (0.05 M 5°C)	14.0525 [48.329]	
$Nb_{16}Ge_3$-CO_3 (0.05 M 25°C)	12.0614 [42.211]	

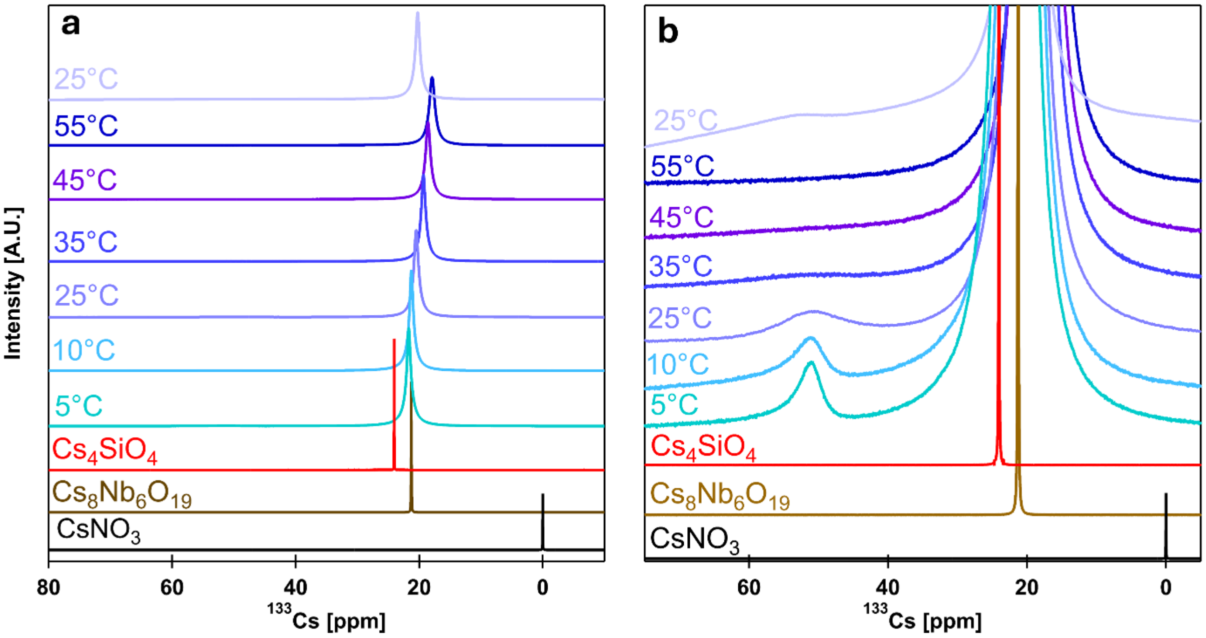

Figure S6. Variable temperature solution ^{133}Cs NMR of **$Nb_{16}Si_3$-CO_3**; (a) shows the full data collection window and (b) is an expanded view of the second peak, indicating bonding to the POM. Cs_4SiO_4, $Cs_8[Nb_6O_{19}]$, and $CsNO_3$ are shown for comparison.

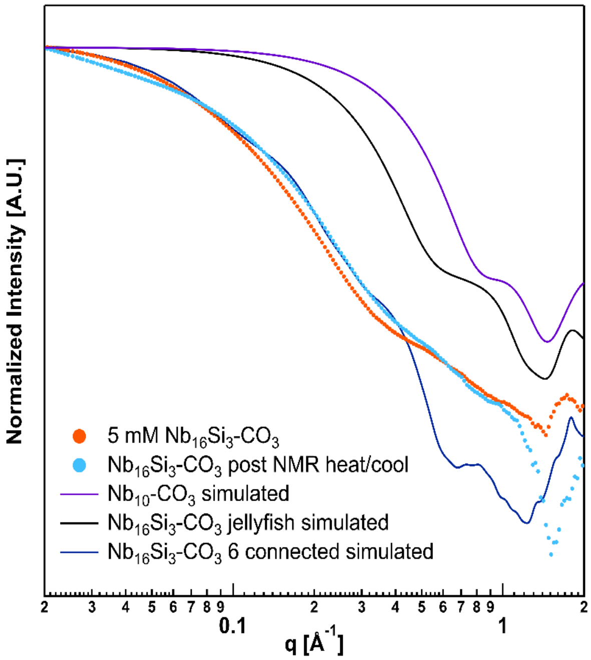

Figure S7. SAXS of freshly prepared **$Nb_{16}Si_3$-CO_3** (5 mM) compared to the sample after the ^{133}Cs NMR heating/cooling cycle. Both scattering curves are similar, indicating the cluster is stable and intact after heating, and still matches a 6-connected cluster model.

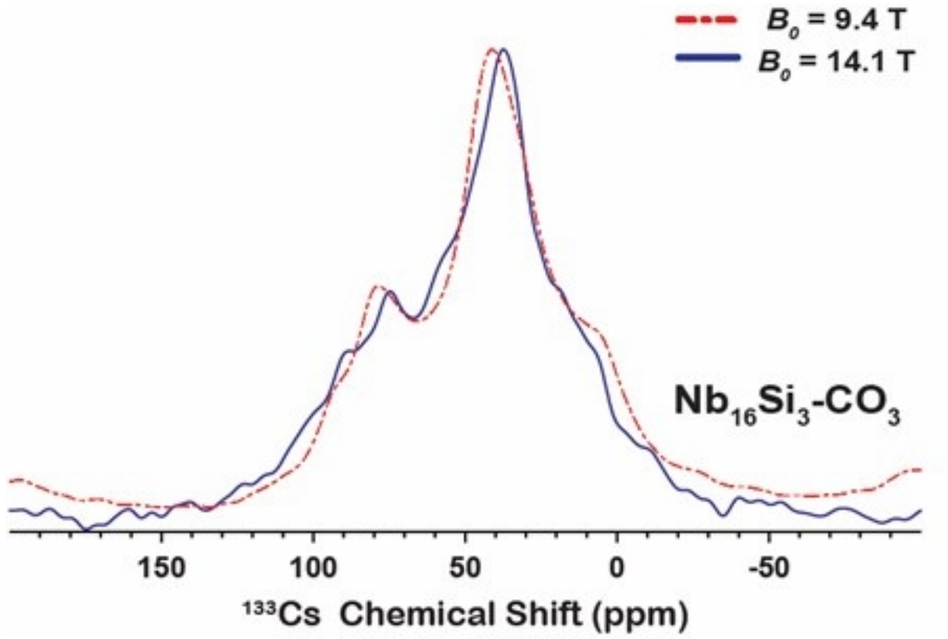

Figure S8. ^{133}Cs ssNMR spectra of **$Nb_{16}Si_3$-CO_3** acquired at magnetic fields of 9.4 T and 14.1 T. The line shape at 9.4 T (red dashed line) matches the line shape of the 14.1 T NMR spectrum, suggesting that the quadrupolar broadening is negligible.

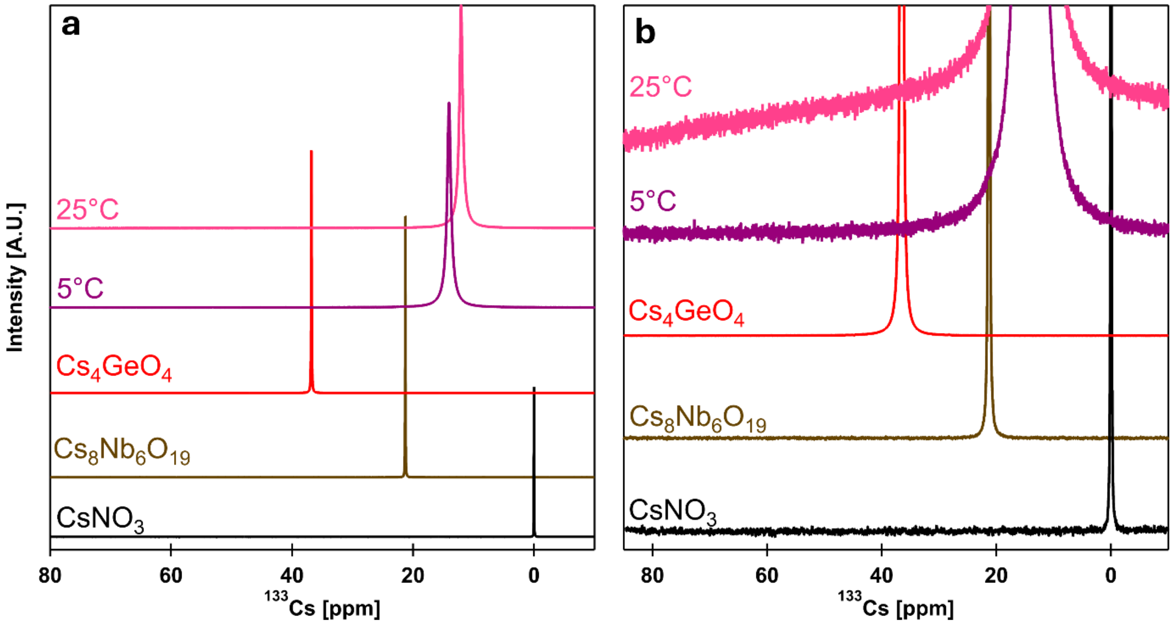

Figure S9. [133]Cs NMR comparing the Cs-Nb_6 and Cs_4GeO_4 starting material to the crystallized **$Nb_{16}Ge_3$-CO_3** at 5 °C and 25 °C. (a) shows the full data collection range, and (b) shows the expanded region, indicating the POM-bound Cs is not present.

6. Small-angle X-ray Scattering of Redissolved Crystals.

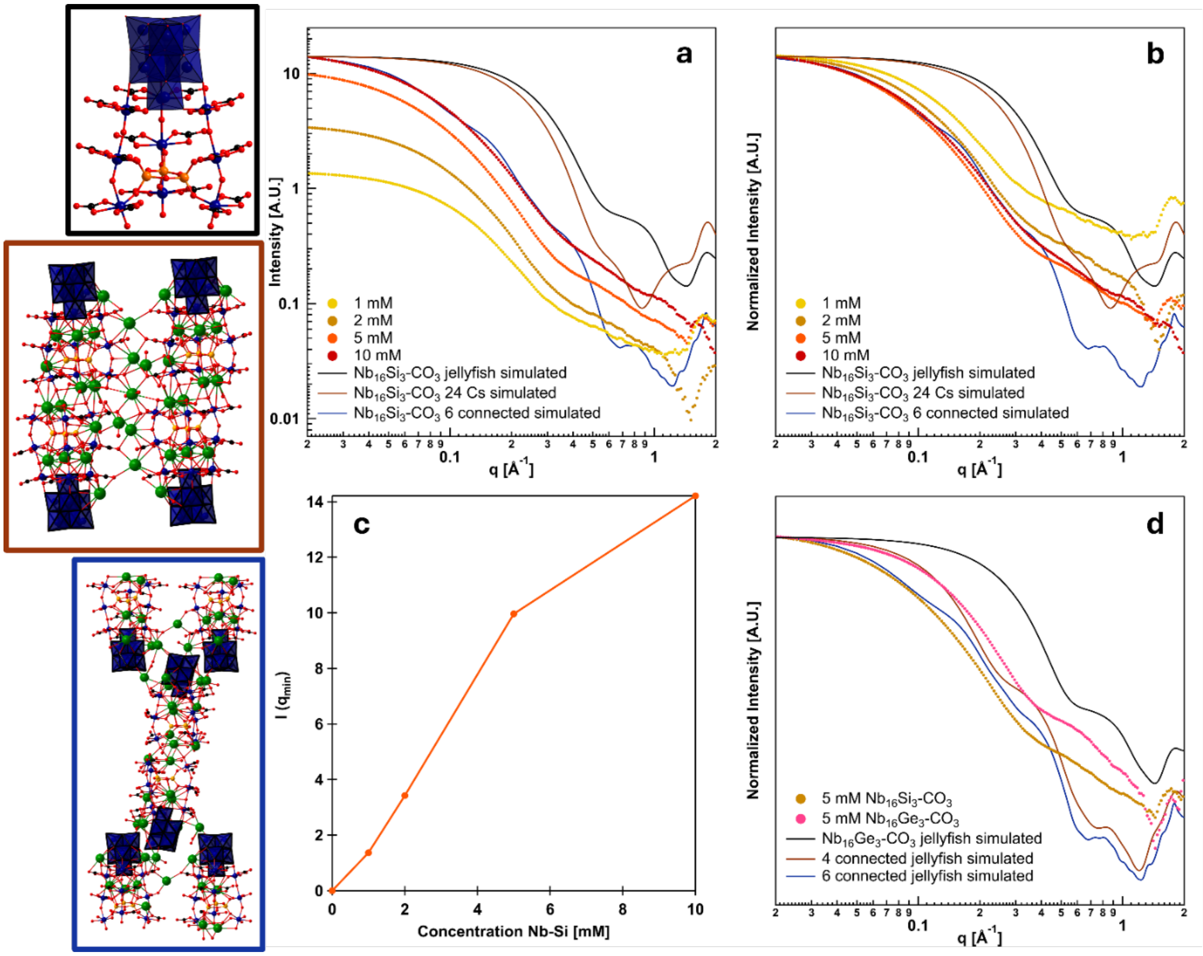

Figure S10. Experimental scattering curves of 1, 2, 5, and 10 mM **$Nb_{16}Si_3$-CO_3** (yellow, gold, orange, and red circles, respectively) compared to the simulated cluster scattering (solid lines), shown on the left with colors representing the simulated curves in the figure (i.e., single cluster (black), Cs-linked tetramer (brown), and Cs-linked hexamer (dark blue) shown at the original scattering intensity (a), and (b) normalized scattering intensity highlighting that no simulated scattering curve is a perfect match to the experimentally observed one. (c) Intensity (q_{min}) as a function of concentration of **$Nb_{16}Si_3$-CO_3** to determine if association changes with increasing concentration. The scattering intensity increases linearly until 10 mM, suggesting similar connectivity between Cs^+ and POMs. At 10 mmolar, the scattering intensity likely deviates from linearity due to X-ray attenuation from the Cs. Due to this, we used a maximum concentration of 5 mM for all other SAXS measurements of the clusters. (d) Experimental scattering of **$Nb_{16}Si_3$-CO_3** more closely matches the 6-connected hexamer (dark blue), whereas **$Nb_{16}Ge_3$-CO_3** is a better fit with a 4-connected tetramer (brown).

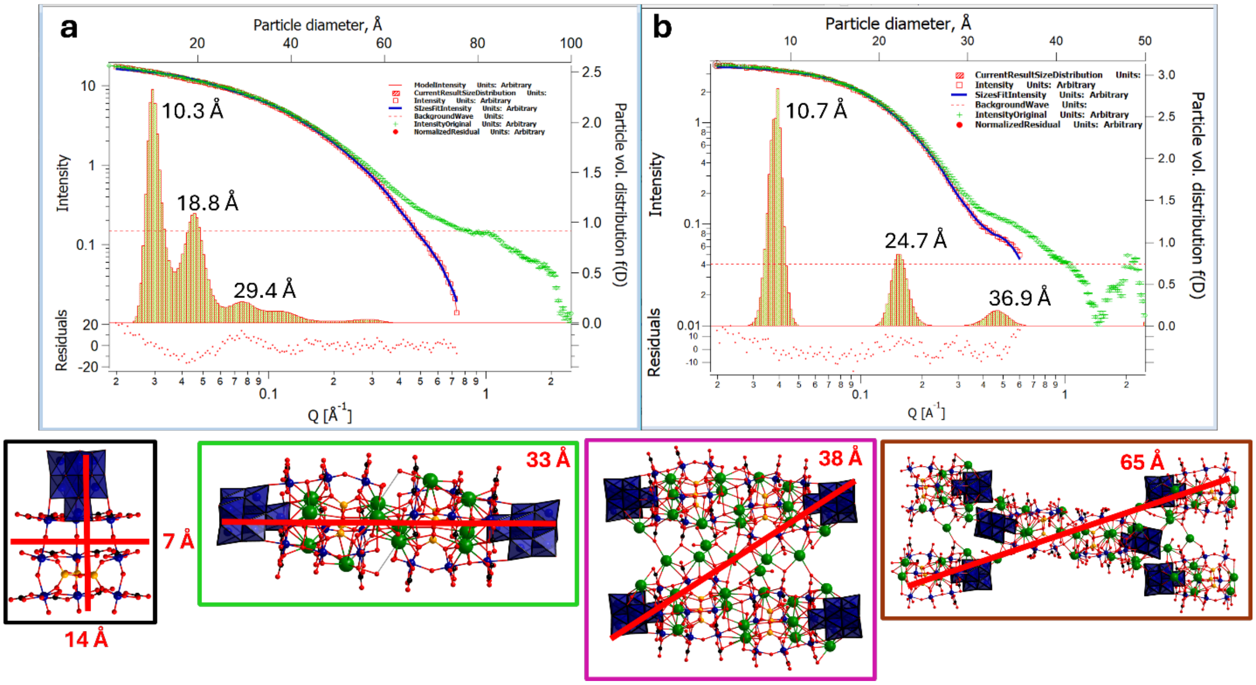

Figure S11. Size distribution analysis using the SAXS data of (a) **$Nb_{16}Si_3$-CO_3** and (b) **$Nb_{16}Ge_3$-CO_3**. Relevant cluster aggregates and sizes are represented below.

7. ^{29}Si NMR Spectroscopy.

Table S10. ^{29}Si NMR summary of pH, chemical shifts, and full width at half maximum for control experiments, crystal, and gel forming conditions.

Sample	pH	chemical shift (ppm) [FWHM, Hz]						
Cs_4SiO_4 (0.7 M Si)	14	-71.9751 [10.71]	-79.7632 [15.299]	-81.7109 [9.22]	-81.9458 [8.36]	-87.9128 [18.7]	-88.9571 [13.68]	-89.4971 [10.20]
Na_4SiO_4 (0.7 M Si)	14	-71.5653 [13.218]	-79.4981 [14.94]	-81.5012 [12.153]	-87.8188 [16.96]	-89.4701 [11.87]		
Cs_4SiO_4 (0.7 M Si)	12	-72.0626 [9.13]	-80.4631 [10.29]					
$CsNb_6$ + Cs_4SiO_4, 0 hrs CO_2	14	-71.1937 [19.421]	-79.9847 [22.422]	-80.8598 [9.29]	-82.1526 [17.41]	-82.3429 [6.09]		
$CsNb_6$ + Cs_4SiO_4, 1 hrs CO_2	14	-72.1032 [11.320]	-77.3029 [17.778]	-79.7277 [16.403]	-80.3022 [14.42]	-82.4097 [19.785]	-82.6605 [26.99]	-86.4991 [9.68]
$CsNb_6$ + Cs_4SiO_4, 2 hrs CO_2	13	-72.0030 [5.797]	-80.5779 [5.093]					
$CsNb_6$ + Cs_4SiO_4, 3 hrs CO_2	12	-80.6370 [4.34]						
$CsNb_6$ + Cs_4SiO_4, 3.5 hrs CO_2	12	-80.6351 [4.49]						
$CsNb_6$ + Cs_4SiO_4, 4 hrs CO_2	11.5	-80.6167 [4.54]						
$CsNb_6$ + Cs_4SiO_4, 6 hrs CO_2	11.5	-80.5841 [7.74]						

$CsNb_6$ + Cs_4SiO_4, 8 hrs CO_2	11	no signal						
$CsNb_6$ + Cs_4SiO_4, 18 hrs CO_2	10	-88.7213 [8.85]						
$Nb_{16}Si_3$-CO_3 rediss. (0.1 M)	10	-88.6322 [9.345]						
$CsNb_6$ + SiO_2 (gel cond.), 0 hrs CO_2	14	-72.0853 [9.658]	-80.1362 [19.903]	-80.4469 [33.366]	-82.4928 [30.662]	-88.5105 [39.331]	-89.6940 [24.864]	
$CsNb_6$ + SiO_2 (gel cond.), 2 hrs CO_2	12	-80.5433 [8.52]						
$CsNb_6$ + SiO_2 (gel conds), 4 hrs CO_2	11.5	-80.5428 [9.15]						
$CsNb_6$ + SiO_2 (gel cond), 8 hrs CO_2	11	-80.5236 [10.32]						
Cs_4SiO_4 (0.7 M Si), 3 hrs CO_2	11.5	-72.1174 [1.672]	-80.7383 [3.13]					
Cs_4SiO_4 (0.7 M Si), 4 hrs CO_2	11	-71.9229 [2.541]						

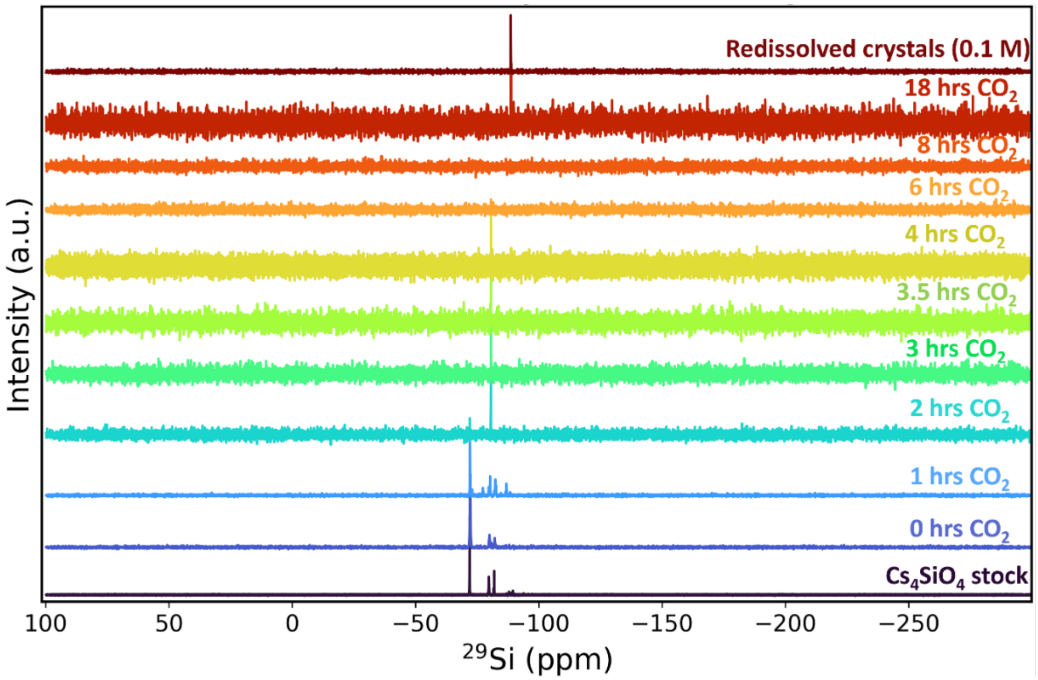

Figure S12. The full ^{29}Si spectral window was used to collect data for studying the reaction mechanism of Cs-Nb_6 with Cs_4SiO_4 and CO_2 infusion.

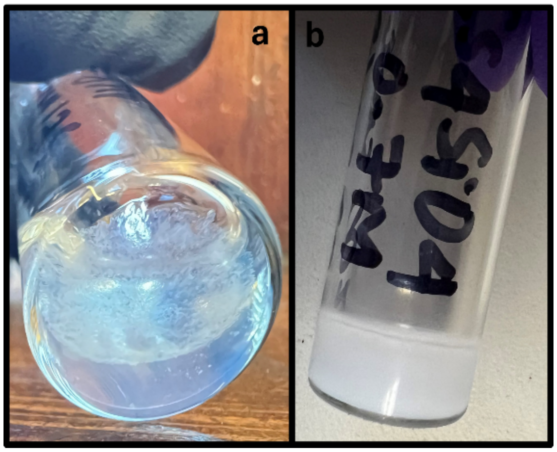

Figure S13. (a) Cs-Nb_6 and Cs_4SiO_4 (0.7 M), after 8 hours of CO_2 exposure, have a gel-like precipitate, which is most apparent between 6-8 hours. The gel-like precipitate is mostly redissolved after 18-24 hours when crystal growth begins. (b) Cs_4SiO_4 (0.7 M) without niobium present after 3 hours of CO_2 exposure. There is more precipitation compared to (a), and it does not redissolve or clear up over time (likely a mixture of SiO_2 and Cs_2CO_3).

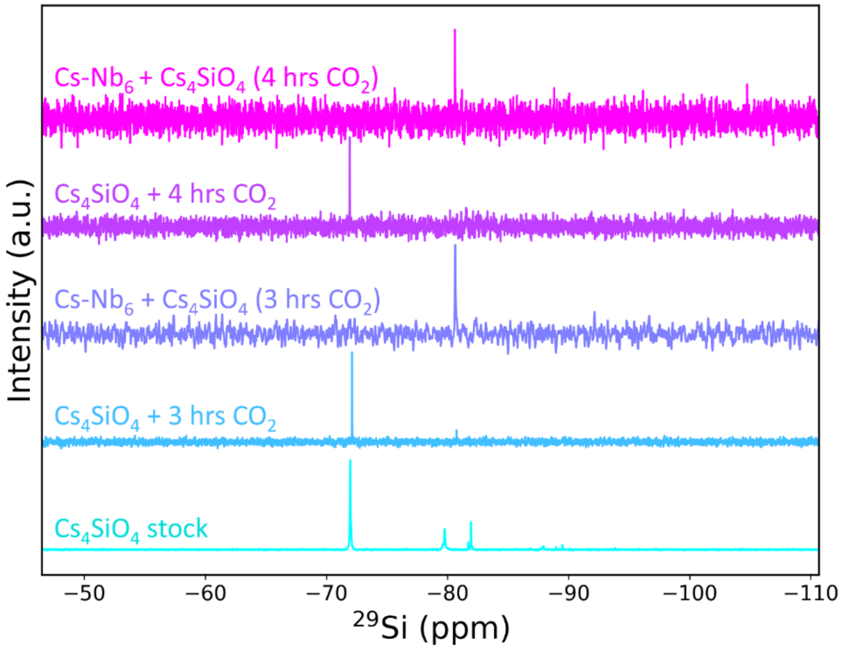

Figure S14. Solution ^{29}Si NMR spectra of Cs_4SiO_4 with and without Cs-Nb_6 at 3- and 4-hours CO_2 exposure. Without Cs-Nb_6 (3 hrs), the Cs_4SiO_4 spectrum has two peaks at -72.1 and -80.7 ppm, showing that both free and oligomeric silicate are present. This contrasts with the 3-hour CO_2 spectrum with Cs-Nb_6, which only has one peak at -80.6 ppm, showing that the presence of niobium encourages oligomerization. Making the same comparison after 4 hours of CO_2, the oligomeric peak for Cs_4SiO_4 alone is gone, and only the peak for free silicate remains, whereas with niobium, the oligomeric peak (trimer) is still present.

8. Characterization of precipitates, SEM/EDS

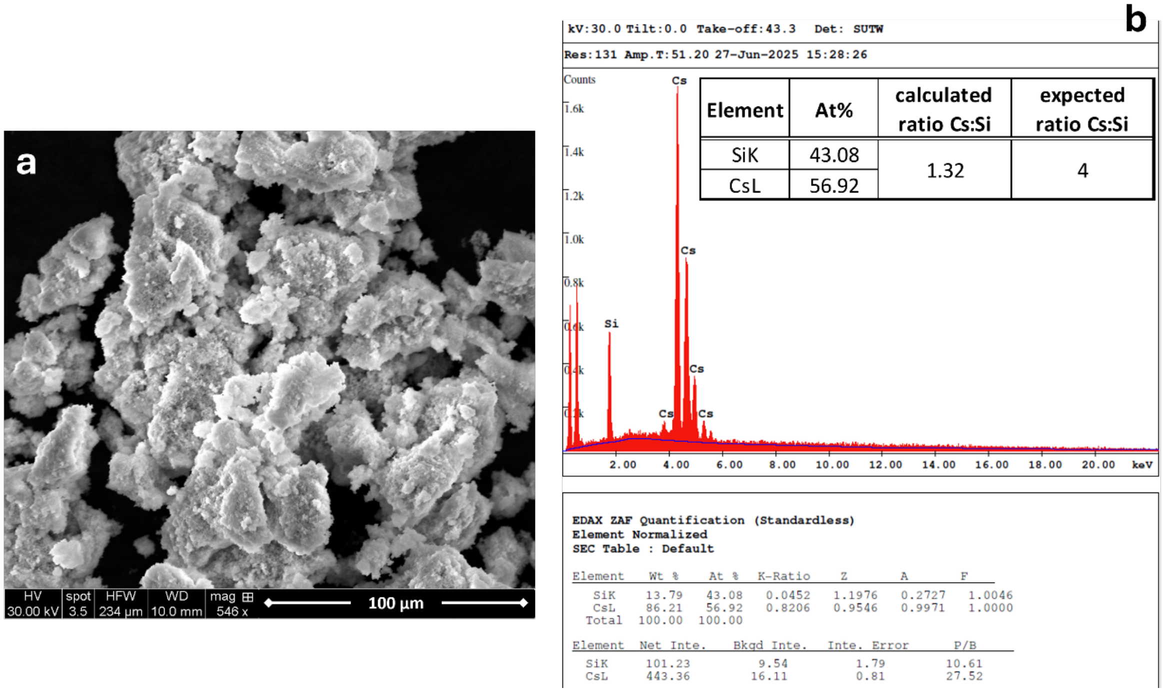

Figure S15. SEM (a) and (b) EDS of the cesium silicate precipitate after 4 hours of CO_2 exposure of the Cs_4SiO_4 solution.

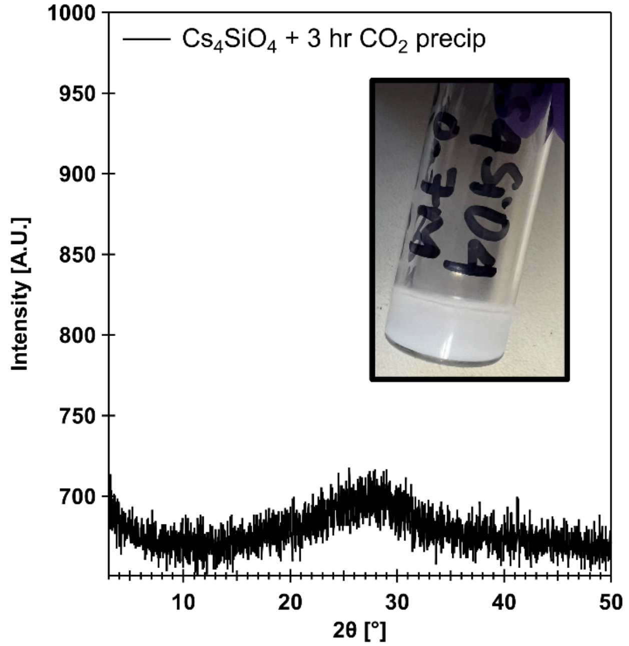

Figure S16. Powder X-ray diffraction of the precipitate obtained with Cs_4SiO_4 after 3 hours of CO_2 (inset picture) indicates it is amorphous (no Bragg diffraction peaks).

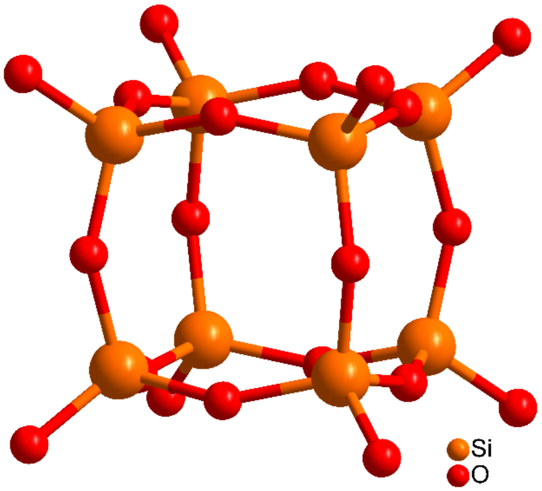

Figure S17. Structure of octasilicate, $[Si_8O_{20}]^{8-}$, previously reported by Smolin et al.,[8] and Wiebcke et al.,[9] and charge balanced by tetramethylammonium (TMA) counterions. Fully formulated as $TMA_8[Si_8O_{20}] \cdot 65\ H_2O$.

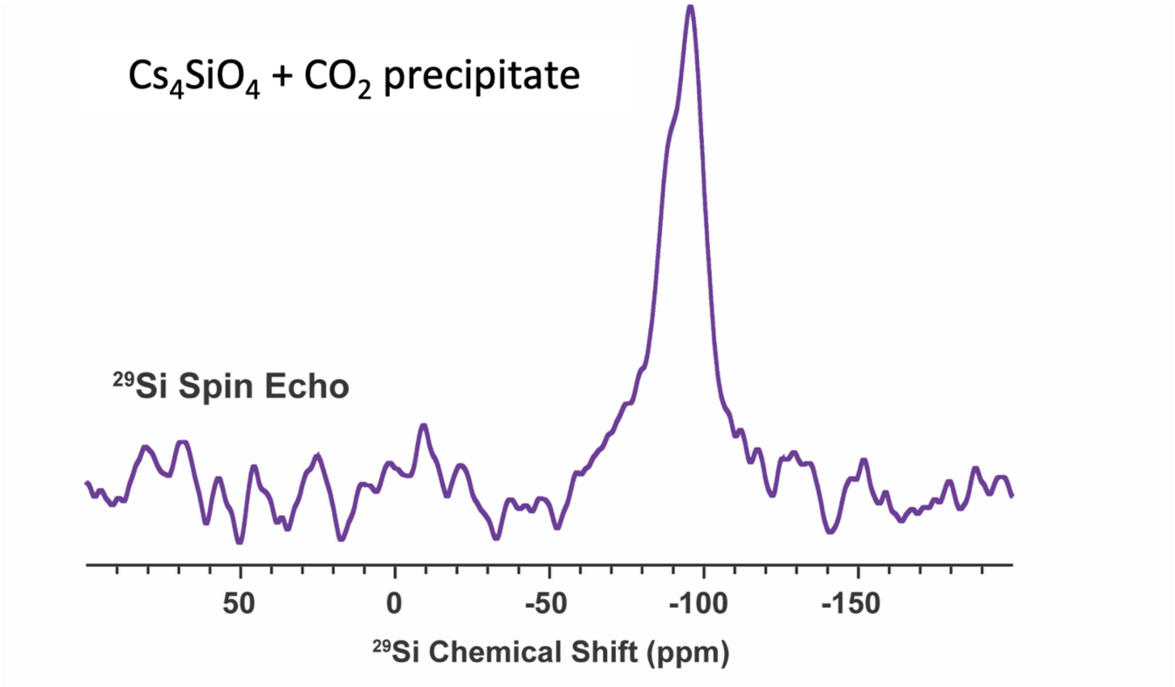

Figure S18. MAS ^{29}Si ssNMR spectrum of the precipitate obtained after exposing Cs_4SiO_4 to CO_2.

Table S11. Summary of ^{29}Si ssNMR experimental parameters.

Figure	Compound	B_0 (T)	MAS (kHz)	Recycle Delay (s)	# of scans	Exp. Time (hr)
Figure 5[a]	**$Nb_{16}Si_3$-CO_3**	9.4	10	200	1024	56
Figure 5[a]	Nb-Si gel	9.4	10	200	512	28
Figure S18	$Cs_4SiO_4 + CO_2$	9.4	10	250	352	24

[a]*CPMG detection was used. Each CPMG echo was 4 rotor cycles in duration, with 80 spin echoes acquired.*

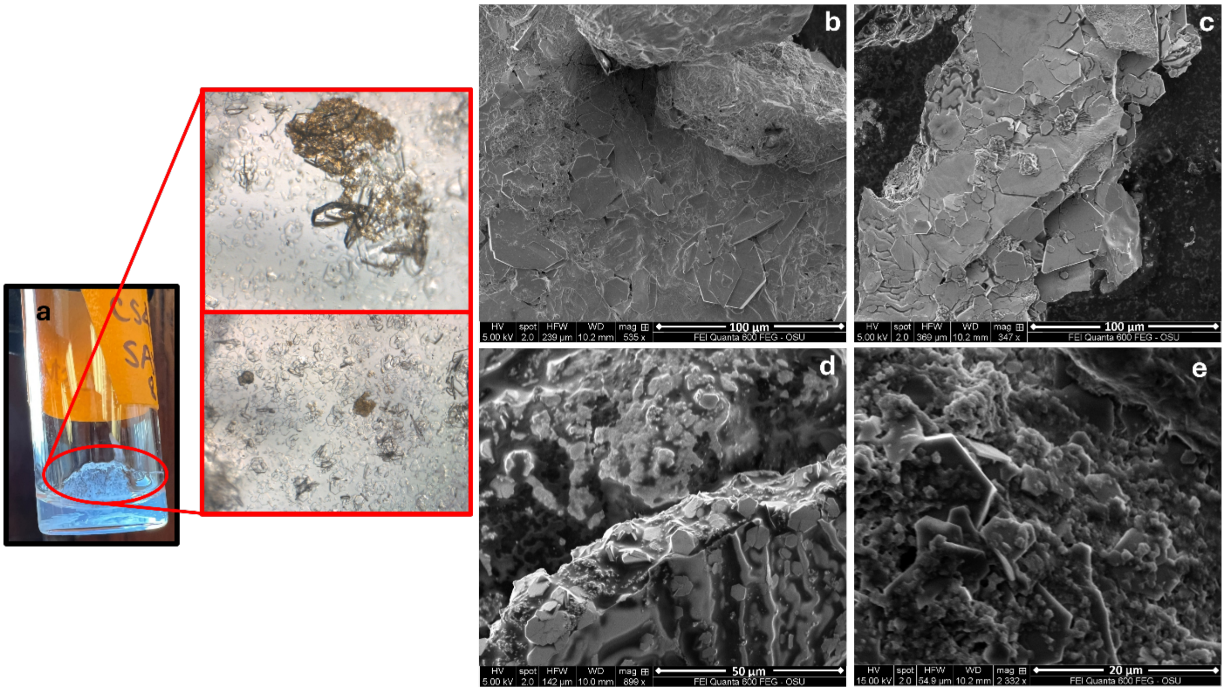

Figure S19. (a) macroscopic view of the gel-like precipitate in the $Nb_{16}Si_3$-CO_3 reaction system after 8 hours of CO_2, with red boxes being the view under the microscope. (b) – (e) SEM images of the gel-like precipitate. $Nb_{16}Si_3$-CO_3 crystals do appear to be growing out of the larger pieces of precipitate.

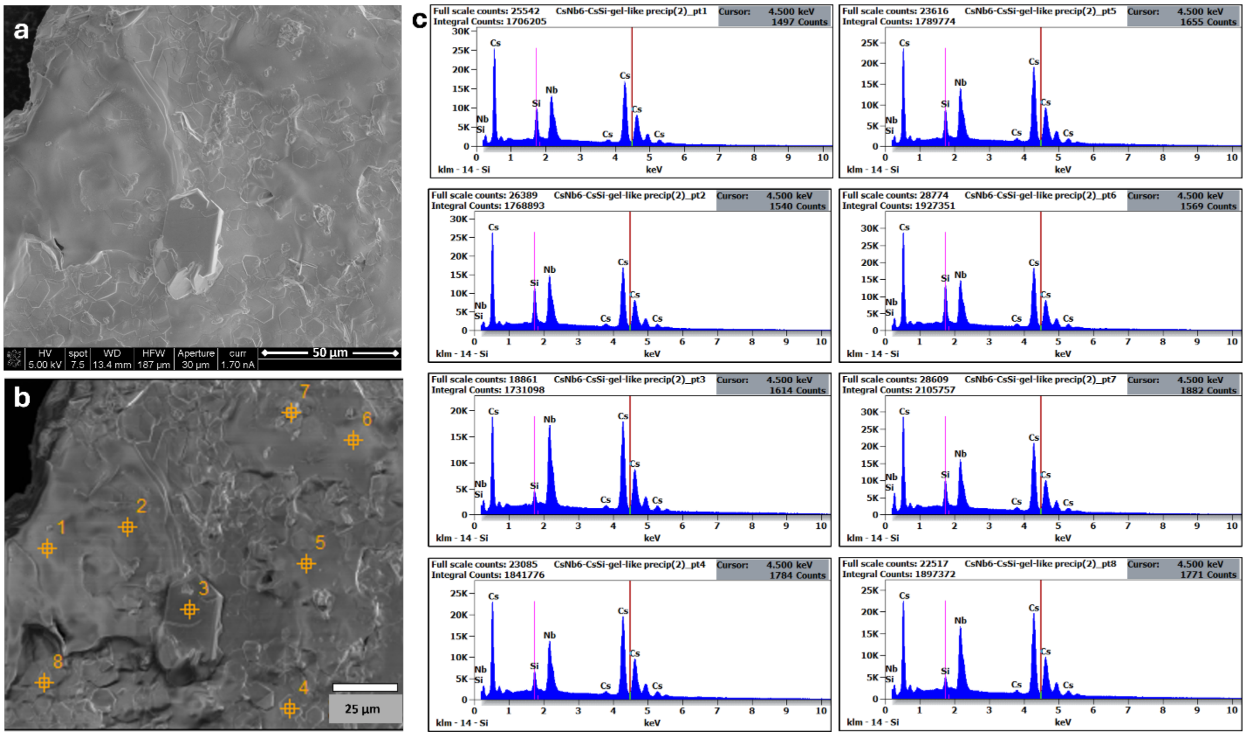

Figure S20. (a) and (b) SEM images corresponding to the point-by-point EDS analysis, with the spectra of each point shown in (c). We chose points that had the typical $\mathbf{Nb_{16}Si_3\text{-}CO_3}$ morphology (points 3, 4, and 8) and compared them to the larger piece (points 1, 2, 5, 6, and 7) they were 'growing' out of. Summarizing the EDS results in **Table S12**, we see that the points corresponding to the crystals have a lower Si content compared to the larger precipitate.

Table S12. Summary of the point-by-point EDS analysis of the gel-like precipitate of $Nb_{16}Si_3$-CO_3, showing that the Si content is lower for the points on crystals (points 3, 4, and 8, marked with '#'). Compared to the larger precipitate, their ratio of niobium to silicate is closer to what we expect from the crystal formula.

	At% Si	At% Nb	At% Cs	ratio Nb: Si	expected ratio Nb: Si
**Pt 1**	19.3	22.9	57.6	1.2	5.3
**Pt 2**	21.5	24.2	54.2	1.1	
**#Pt 3**	6.52	30.7	62.6	4.7	
**#Pt 4**	11.1	23	65.8	2.1	
**Pt 5**	16.1	22.7	61.1	1.4	
**Pt 6**	23.7	21.9	54.2	0.9	
**Pt 7**	16.1	23.2	60.6	1.4	
**#Pt 8**	6.87	27.9	65.1	4.1	

9. SAXS time-study

Table S13. Concentration of niobium, silicon, and cesium or sodium for relevant SAXS solutions, along with their scattering intensity at minimum q (Å).

Sample	Concentration (M)			
	Nb	Si	Cs or Na	I_0 (q_{min})
$Nb_{16}Si_3$-CO_3 (5 mM Nb_{16})	0.08	0.02	0.12	9.78
Cs-Nb_6 + Cs_4SiO_4 + 6 hrs CO_2 (10 mM Nb_6)	0.06	0.01	0.10	3.65
Cs-Nb_6 + Cs_4SiO_4 + 18 hrs CO_2 (10 mM Nb_6)	0.06	0.01	0.10	3.34
Na_4SiO_4 + 6 hrs CO_2 (1 M Si)	0	1.0	4	3.65

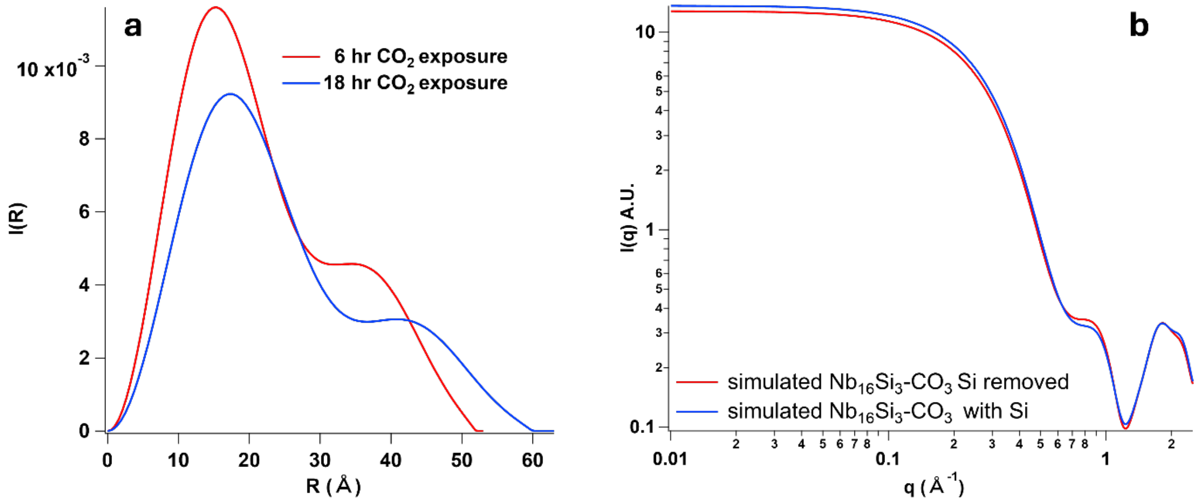

Figure S21. (a) SAXS analysis showing pair-distance distribution function (PDDF) of Cs-Nb_6 with Cs_4SiO_4 after 6 and 18 hours of CO_2 exposure. The size of the cluster at 18 hours is slightly larger compared to that after 6 hours. (b) Simulations of **$Nb_{16}Si_3$-CO_3** with and without the cyclic trimer. Little change in the scattering indicates that the Nb-POM contributes most to the overall intensity.

10. Raman and FTIR Spectroscopy; Gel characterization

Table S14. The percentage of each component present in the gel, as well as their normalized ratios.

Sample	% Alkali	% X (Si, Ge or PO_4)	% Nb	Ratio Alkali: X	Ratio X: Nb	Ratio Nb: Alkali
$Nb_{16}Si_3$-CO_3	62	4.1	34	0.63	0.35	0.02
$Nb_{16}Ge_3$-CO_3	60	2.5	37	0.51	0.38	0.11
Nb-Si gel	49	14	37	0.61	0.38	0.02
Nb-Ge gel	55	3.2	41	0.56	0.42	0.02
Nb-PO_4 gel	53	6.8	40	0.54	0.41	0.05

Table S15. Raman frequencies (cm^{-1}) of **$Nb_{16}Si_3$-CO_3** and the Nb-Si gel, compared to literature values. Frequencies observed in both the crystalline and gel systems match literature values for the Si-O and Si-O oligomeric bonds, suggesting that the trimer is likely present in the Nb-Si gel.

**$Nb_{16}Si_3$-CO_3**	**average bond distance (Å)**	**Nb coord. number**	***predicted Nb-O frequency of crystals (cm^{-1})**	**frequency of Cs_4SiO_4 (cm^{-1})**	**frequency of crystals (cm^{-1})**	**frequency of gel (cm^{-1})**	**Literature frequency[10–12] (cm^{-1})**
μ_6-O-Nb_6	2.37	6	274.2		315.4	314.4	
μ_2-O-Nb (chains)	2.26	7	342.6		383.3	390.6	
μ_2-O-Nb (chains)	2.18	7	394.2	446.0	438.3		435.8 (Si-O-Si)
μ_2-O-Nb (chains)	2.14	7	425.3		488.4	487.0	
Nb-O carbonate	2.12	7	447.3		534.7	543.4	
μ_2-O-Nb (Nb_7)	2.01	6	548.5		562.7		565.3 (Si-O-Si)
Nb to μ_2-O-Si	1.98	7	580.9	603.0	658.1		683.8 (Si-O-Si)
μ_2-O-Nb_2 (Nb_6)	1.97	6	594.3		692.8		697.9; 708, 693 (ring mode)
Nb to μ_2-O-Si	1.96	7	603.7		729.0	714.5	
Nb-O (Nb_7)	1.88	6	710.2	772.0	777.2	771.4[a]	788.4 (O-Si-O)
Nb-O (chains)	1.81	6	810.7		822.1	821.1	
Nb-O (chains)	1.78	7	855.6		848.6	844.2	857.2 (O-Si-O)
Nb-O (chains)	1.78	7	858.4		858.7		858.1 (O-Si-O)
Nb=Oyl (Nb_6)	1.77	6	864.4		890.5	889.6	
Nb=Oyl (chains)	1.72	7	956.2	926	955.6		947/935 (Si-O)
$CsHCO_3$					1012		1010/994 (Si-O)
Cs_2CO_3				1060	1054	1061	

** Predicted frequencies were calculated using Hardcastle and Wachs' correlation[13] between frequency and Nb-O bond lengths ($y = 25922 \exp(-1.9168x)$, where x is the Nb-O bond length (Å)), combined with the bond distances in the $Nb_{16}Si_3$-CO_3 crystal structure. a - small shoulder*

Table S16. Raman frequencies (cm^{-1}) of **$Nb_{16}Ge_3$-CO_3** and the Nb-Ge gel, compared to literature values. Frequencies observed in both the crystalline and gel systems match literature values for the Ge-O and Ge-O oligomeric bonds, suggesting that the trimer is likely present in the Nb-Ge gel.

$Nb_{16}Ge_3$-CO_3	average bond distance (Å)	Nb coord. number	*predicted Nb-O frequency of crystals (cm^{-1})	frequency of Na_4GeO_4 (cm^{-1})	frequency of crystals (cm^{-1})	frequency of gel (cm^{-1})	Literature frequency[14,15] (cm^{-1})
μ_6-O-Nb_6	2.37	6	273.7				
μ_2-O-Nb (chains)	2.28	7	330.6		313.42	312.45	
μ_2-O-Nb (chains)	2.20	7	383.0		399.23	385.25	
μ_2-O-Nb (chains)	2.14	7	429.6		442.62		439 (Ge-O)
Nb-O carbonate	2.13	7	440.3		483.12	482.16	485 (Ge-O)
μ_2-O-Nb (Nb_7)	2.00	6	556.8		537.6	543.39	525; 527 (Ge-O-Ge)
Nb to μ_2-O-Ge	1.99	6	568.7		561.71	561.22	563 (Ge-O-Ge)
μ_2-O-Nb_2 (Nb_6)	1.98	7	585.3		577.13^a		500-570 (ring band)
Nb to μ_2-O-Ge	1.95	7	620.1	663.0	658.13^a	657.17^a	620 (ring strain)
Nb-O (Nb_7)	1.88	6	704.4		713.57	712.61	
Nb-O (chains)	1.82	6	784.3	777.2	771.91^a		790 (Ge-O-Ge)
Nb-O (chains)	1.77	6	877.6		803.25^a		
Nb-O (chains)	1.76	7	886.1		826.87	816.26	
Nb=Oyl (Nb_6)	1.75	7	905.2		844.23	844.23	
Nb=Oyl (chains)	1.72	7	955.3		887.62	888.58	
$CsHCO_3$					1014.4	1017.3	
Cs_2CO_3				1068.4	1057.3	1058.3	

** predicted frequencies were calculated using Hardcastle and Wachs' correlation[13] between frequency and Nb-O bond lengths (y = 25922 exp(-1.9168x), where x is the Nb-O bond length (Å)), combined with the bond distances in the $Nb_{16}Ge_3$-CO_3 crystal structure.*
a - small shoulder

Table S17. Raman frequencies (cm^{-1}) of Cs-Nb_6 and the Nb-PO_4 gel, compared to literature values. Frequencies observed in the gel match literature values and correspond to both free phosphate and O-P-O bonding modes.

**$[Nb_6O_{19}]^{8-}$**	**average bond distance (Å)**	**Nb coord. number**	***predicted Nb-O frequency (cm^{-1})**	**frequency of Cs-Nb_6 (cm^{-1})**	**frequency of Cs_3PO_4 (cm^{-1})**	**frequency of Nb-PO_4 gel (cm^{-1})**	**Literature frequency[16–19] (cm^{-1})**
μ_6-O-Nb_6	2.36		281.5			311.97	
						379.95	
						445.52	420 (free PO_4^{3-})
				468.18		483.12	483 (internal PO_4^{3-})
				535.19	530.85^a	566.53	564 (free PO_4^{3-})
μ_2-O-Nb_2	1.95		612.3			631.61	631 (internal PO_4^{3-})
		6		742.98		716.47	
						814.34	
				829.76		844.23	
					853.87	866.4	
Nb=Oyl	1.80		816.0	880.39	989.83	888.1	
$CsHCO_3$						1016.8	886-1046 (P-O-P)
Cs_2CO_3				1068.4	1067.9	1058.3	
						1124.8	1118 (O-P-O)

** predicted frequencies were calculated using Hardcastle and Wachs' correlation[13] between frequency and Nb-O bond lengths (y = 25922 exp(-1.9168x), where x is the Nb-O bond length (Å)), combined with the bond distances in the $Cs_8Nb_6O_{19}$ crystal structure.*

a – shoulder

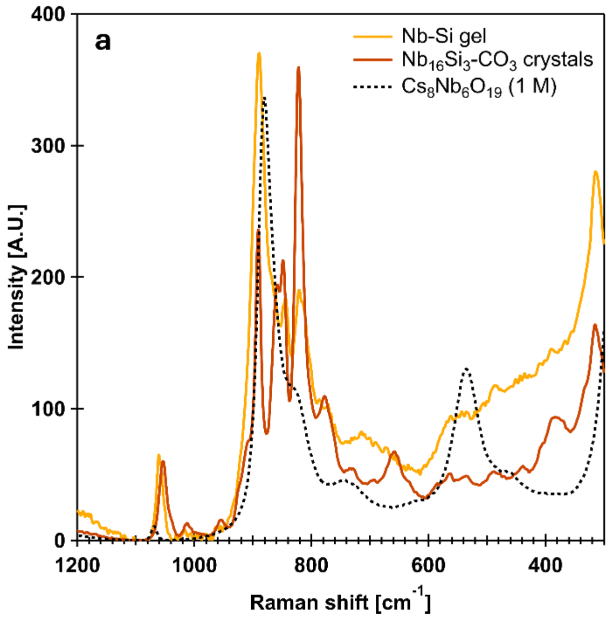

Figure S22. Raman comparison of the gel and crystal systems for (a) **$Nb_{16}Si_3$-CO_3** and Nb-Si gel, and (b) **$Nb_{16}Ge_3$-CO_3** and Nb-Ge gel. In both cases, the gels have many similar bonding frequencies, indicating that the gel system has an intact Nb-POM that is part of a larger gel network with Si or Ge oligomers.

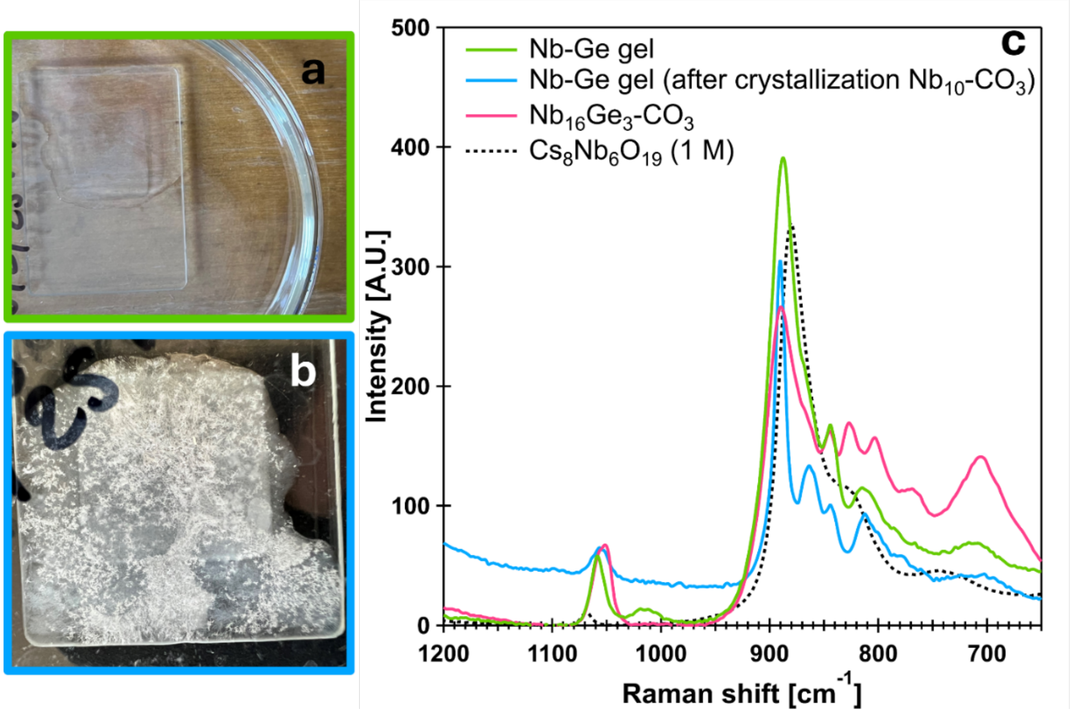

Figure S23. (a) The Nb-Ge gel was placed on a slide to dry and eventually (~3 weeks later) crystallized on the slide (b). X-ray crystallography confirmed that these crystals were Nb_{10}-CO_3. (c) Raman spectra of the recrystallized material compared to the fresh Nb-Ge gel have similar Nb-O features from ~ 900-800 cm^{-1}, proving that the gel contains an intact Nb-carbonate POM.

11. ^{31}P NMR Spectroscopy.

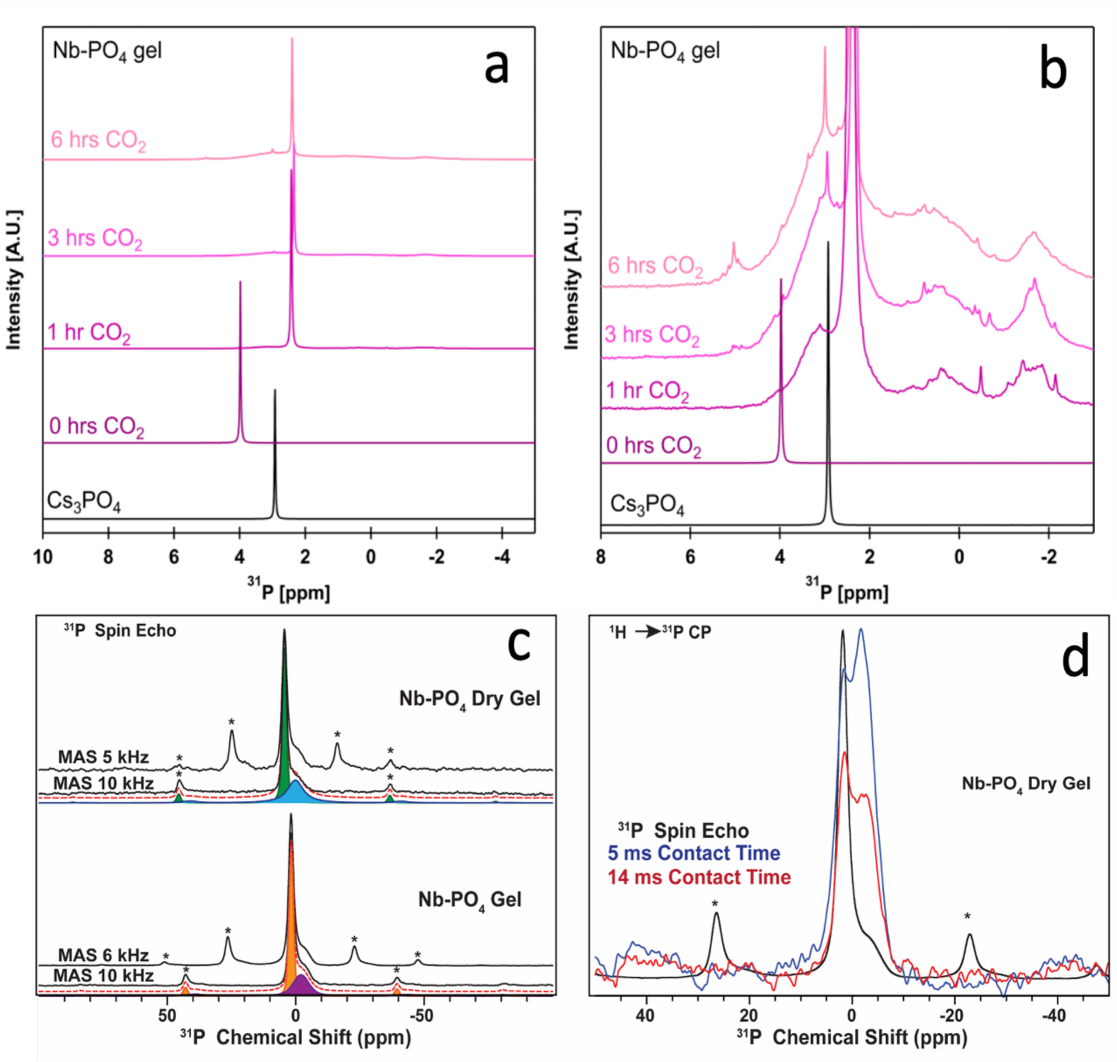

Figure S24. (a) ^{31}P solution NMR spectra of Cs-Nb_6 with Cs_3PO_4 over time with continued CO_2 exposure. Magnified regions are shown in (b). Significant changes to the spectrum occur after one hour of CO_2, with multiple new broad peaks appearing up and downfield from Cs_3PO_4 or Cs_3PO_4 with Cs-Nb_6 (0 hours). A peak at ~ 5 ppm grows in intensity over time and is similar to the previously reported chemical shifts of PNb_{12} and PNb_{14}.[20] (c) MAS ^{31}P ssNMR spin echo spectra of the **Nb-PO$_4$** gel and **Nb-PO$_4$** dried gel obtained with a magnetic field of 14.1 T. (d) ^{1}H→^{31}P CP of **Nb-PO$_4$** dried gel with various contact times. Asterisks (*) indicate spinning sidebands.

Table S18. Summary of ^{31}P ssNMR experimental parameters.

Figure	Compound	B_0 (T)	MAS (kHz)	Recycle Delay (s)	# of scans	Exp. Time (hr)
Figure S25	Nb-PO_4 gel	14.1	6	26	624	4.5
Figure S25	Nb-PO_4 gel	14.1	10	26	128	0.9
Figure S25	Nb-PO_4 dry gel	14.1	5	26	16	0.1
Figure S25	Nb-PO_4 dry gel	14.1	10	26	16	0.1
Figure S25	Nb-PO_4 dry gel	14.1	10	0.3	8192	0.7
Figure S25	Nb-PO_4 dry gel	14.1	10	0.3	8192	0.7

Table S19. Chemical shifts (ppm) and full width at half maximum for the ^{31}P NMR spectra.

Sample	ppm	FWHM (Hz)
1 M Cs_3PO_4	2.9391	5.851
Cs-Nb_6-Cs_3PO_4 t = 0 hrs CO_2	3.9821	6.841
Cs-Nb_6-Cs_3PO_4 t = 1 hrs CO_2	3.1138	147.392
	2.4311	9.291
	0.4097	broad multiplet
	-0.475	14.174
	-1.4156	40.692
	-1.8407	broad multiplet
	-2.1409	22.279
	-6.6935	8.455
	-6.9593	6.852
Cs-Nb_6-Cs_3PO_4 t = 3 hrs CO_2	5.0555	40.752
	4.8696	23.928
	3.9329	shoulder
	2.9533	17.403
	2.3523	33.806
	0.7909	35.401
	0.6951	broad multiplet
	0.5585	broad multiplet
	-0.3412	19.252
	-0.4378	10.413
	-0.667	34.664
	-1.6747	26.432
	-2.1245	23.998

	-8.7875	8.87
Cs-Nb_6-Cs_3PO_4 t = 6 hrs CO_2	5.1537	12.043
	5.0382	17.351
	4.9445	9.386
	3.3771	shoulder
	3.0026	22.544
	2.4056	7.404
	0.7815	35.475
	0.5687	34.64
	-0.3982	28.469
	-1.6408	broad multiplet
	-8.8384	8.673
	-9.2833	13.769
	-10.7515	13.809

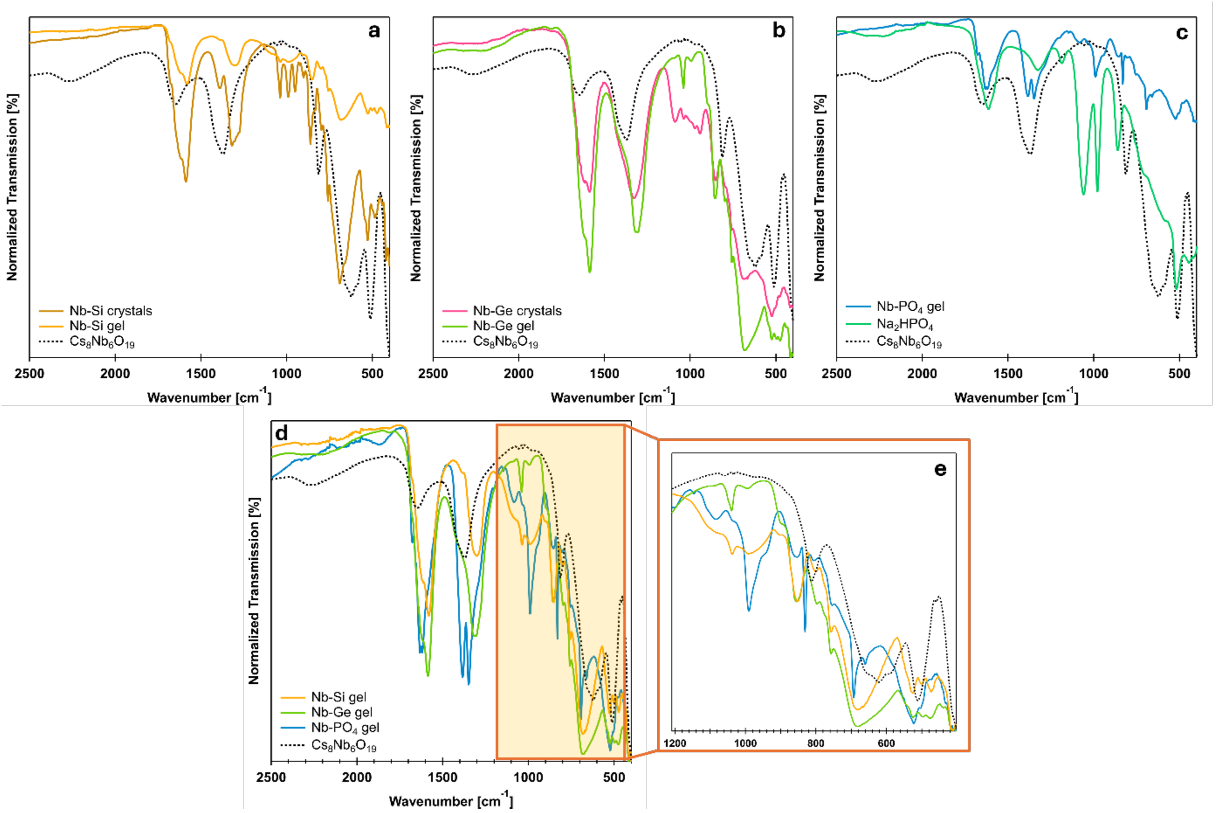

Figure S25. FTIR spectra of the crystalline and gel materials compared to the starting material, Cs-Nb_6. (a) **$Nb_{16}Si_3$-CO_3** and Nb-Si gel. (b) **$Nb_{16}Ge_3$-CO_3** and Nb-Ge gel, (c) Nb-PO_4 and Na_2HPO_4, (d) comparing all three gels (Nb-Si, Nb-Ge, and Nb-PO_4), with magnified spectra in (e) highlighting the new features observed in the gels. Peak assignments are summarized in **Tables S17-S19**.

Table S20. FTIR wavenumbers (cm^{-1}) associated with Cs-Nb_6, Cs_2CO_3, the **$Nb_{16}Si_3$-CO_3** crystals, Nb-Si gel, and reported Si-O literature values. Literature frequencies match those of the crystals and gels, demonstrating that the cyclic trimer is present in the gel material.

**$Cs_8Nb_6O_{19}$ frequency (cm^{-1})**	**Cs_2CO_3 frequency (cm^{-1})**	**Nb-Si crystals frequency (cm^{-1})**	**Nb-Si gel frequency (cm^{-1})**	**Literature frequency[10,12] (cm^{-1})**	**Motion**
		484^{t}	474^{s}	435.8	Si-O_{br}, SiOSi, OSiO
			500^{s}		
512^{s}		528^{s}	525^{s}	546	SiOSi, OSiO, Nb-O
623^{b}					Nb-O
		691^{b}	684vb	683.8	Si-O_{br}, SiOSi, $O_{br}SiO_{br}$
				697.9	SiOSi, OSiO
		759^{a}	757^{a}	743.7	OSiO
		797^{a}	801^{t}	788.4	OSiO
813^{s}					Nb-O
	878	861^{s}	853^{b}	858.1	Si-O, SiOSi, OSiO, (CO_3^{2-})[21]
		901^{t}			
		951^{s}			
		991^{s}			
1033^{t}	1041	1038^{s}	1038^{t}	1022.5	Si-O, SiOSi, OSiO, (CO_3^{2-})[21]
		1276^{a}			
		1319^{b}	1303^{b}		
1371^{b}	1380	1391^{a}			(CO_3^{2-})[21]
	1450				(CO_3^{2-})[21]
1648^{b}			1582^{b}		Si-O, SiOSi, Nb-O
2271vb		2412^{b}			Nb-O

s - sharp
b - broad
vb - very broad
t - small
a - shoulder

Table S21. FTIR wavenumbers (cm^{-1}) associated with Cs-Nb_6, Cs_2CO_3, the **$Nb_{16}Ge_3$-CO_3** crystals, Nb-Ge gel, and reported literature values of other germanate phases. Literature frequencies match those of the crystals and gels, demonstrating that the cyclic trimer is present in the gel material.

$Cs_8Nb_6O_{19}$ frequency (cm^{-1})	Cs_2CO_3 frequency (cm^{-1})	Nb-Ge crystals frequency (cm^{-1})	Nb-Ge gel frequency (cm^{-1})	Literature frequency[14,15] (cm^{-1})	Motion
512[s]		478[a]	475[t]	515	GeOGe (3-membered ring), Nb-O
		524[b]	524[t]	520	ring band (3-membered ring)
623[b]		683[vb]	681[b]	500-600	GeOGe, Nb-O
		757[a]	758[a]		
			797[a]	798	Ge-O
813[s]		851[s]	855[s]	848	Ge-O intracycle, Nb-O
	878			800-850	Ge ions (4 or 6 coordinate), CO_3^{2-}
		943[t]		937	Ge-O ring
		971[t]			
1033[t]	1041	1038[t]	1039[s]		CO_3^{2-}
		1089[a, b]			
1371[b]	1380	1327[b]	1305[b]	1377-1415	GeOGe, Nb-O
	1450	1587[b]	1586[b]		CO_3^{2-}
1648[b]		1621[a]	1621[a]		Nb-O
2271[vb]					Nb-O

s - sharp
b - broad
vb - very broad
t - small
a - shoulder

Table S22. FTIR wavenumbers (cm^{-1}) from Cs-Nb_6, Cs_2CO_3, Na_2HPO_4, and the Nb-PO_4 gel, and reported literature values of other phosphate phases. Literature frequencies match those of the gels. In particular, the frequencies between ~980 and 1200 cm^{-1} imply connectivity between the phosphate groups.

**$Cs_8Nb_6O_{19}$ frequency (cm^{-1})**	**Cs_2CO_3 frequency (cm^{-1})**	**Na_2HPO_4 frequency (cm^{-1})**	**Nb-PO_4 gel frequency (cm^{-1})**	**Literature frequency[17,18,22] (cm^{-1})**	**Motion**
		444[t]			
512[s]		517[s]	523[b]	500-564	isolated PO_4^{3-}, Nb-O
			661[a, t]		
		583[a]		581; 500-564	POP; PO_4^{3-}
623[b]			693[s]		Nb-O
		710[a]		710	POP
			755[a, t]		
813[s]	878	860[s]	831[s]		Nb-O
		980[s]	990[s]	986; 997, 981-994	2(PO), POP; pyrophosphate, tripolyphosphate
1033[t]	1041	1060[s]		1061, 1064	2(PO), Nb-O
			1084[b, t]	1084	pyrophosphate
			1146[t]	1149; 1140 - 1144	2(PO); pyrophosphate
		1186[t]		1188; 1170-1197	2(PO); pyrophosphate
		1326[b]	1348[b]	1289	2(PO)
1371[b]	1380		1384[b]		(CO_3^{2-})[21]
	1450				(CO_3^{2-})[21]
1648[b]		1614[b]	1627[b]		Nb-O
			1676[a]		Nb-O
2271[vb]					Nb-O

s - sharp
b - broad
vb - very broad
t - small
a - shoulder

12. Thermogravimetry-mass spectroscopy and CHN analysis.

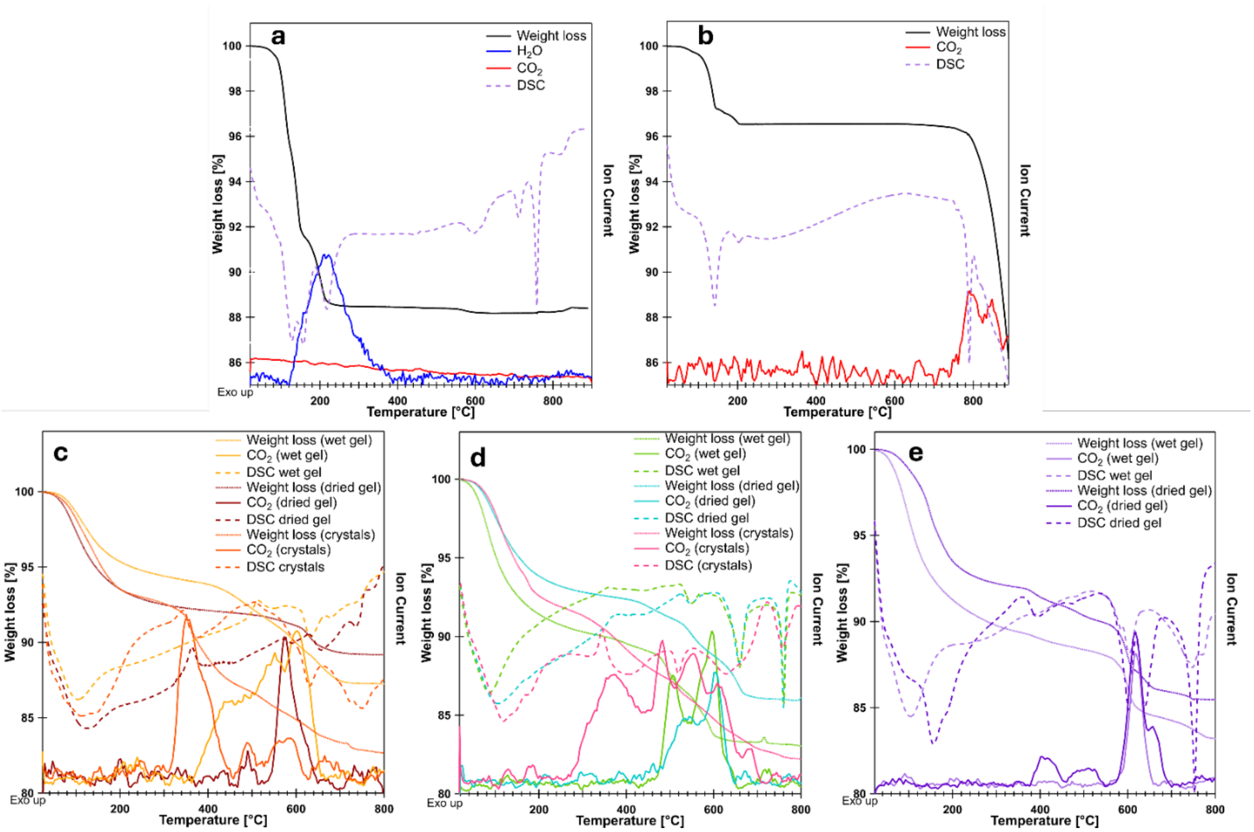

Figure S26. TGA-MS and DSC traces (exothermic is up) of (a) fresh $Cs_8Nb_6O_{19}$, (b) Cs_2CO_3, (c) $Nb_{16}Si_3$-CO_3 crystals, and the Nb-Si gel (wet and dry), (d) $Nb_{16}Ge_3$-CO_3 crystals and the Nb-Ge gel (wet and dry), and (e) Nb-PO_4 wet and dry gel. In (b) the CO_2 signal is cut off at 800 °C due to our instrument capabilities, and the weight loss drops dramatically due to the volatilization of Cs_2O at high temperatures.[23]

Table S23. Summary of TGA-MS data showing the release temperatures of water and CO_2, and the carbonate bonding environment (i.e., alkali or niobium-bound carbonate).

Sample	H_2O release temperature (°C)	CO_2 release temperature (°C)	Assignment
$Cs_8Nb_6O_{19}$. 13.86 H_2O (fresh)	113.4		Lattice water
	142.0		Lattice water
	204.9		Lattice water
Cs_2CO_3	141.8		Water
		738.4	Alkali carbonate
$Nb_{16}Si_3$-CO_3 crystals	116.1		Lattice water
		349.2	Nb-bound carbonate
		489.1	Nb-bound carbonate
		568.6	Nb-bound carbonate
		664.7	Alkali-carbonate
$Nb_{16}Ge_3$-CO_3 crystals	120.7		Lattice water
		370.9	Nb-bound carbonate
		481.5	Nb-bound carbonate
		553.9	Nb-bound carbonate
		608.9	Alkali-carbonate
		682.9	Alkali-carbonate
Nb-Si wet gel	105.2		Water
		465.9	Nb-carbonate
		546.8	Nb-carbonate
		599.0	Alkali-carbonate
Nb-Si dry gel	112.7		Water
		489.0	Nb-carbonate
		570.0	Nb-carbonate
Nb-Ge wet gel	92.81		Water
		503.9	Nb-carbonate
		600.7	Alkali carbonate
Nb-Ge dry gel	108.9		Water
		534.2	Nb-carbonate
		587.5	Alkali carbonate
Nb-PO_4 wet gel	102.8		Water
		619.2	Alkali-carbonate
Nb-PO_4 dry gel	97.66		Water
		398.8	Nb-bound carbonate
		499.0	Nb-bound carbonate
		599.4	Alkali-carbonate
		634.68	Alkali-carbonate
Cs_4SiO_4 4 hr CO_2 precipitate	109.45		Water
		213.4	Bicarbonate
		259.87	Bicarbonate
		402.94	Si-bound carbonate
		739.85	Alkali-carbonate

Table S24. Summary of CHN results and TGA results comparing the weight percents and number of CO_2 per niobium (based on the crystal structure or the Cs-Nb_6 starting material for the gels). By combining this data, we estimate the ratios of each component present.

Sample	% C (CHN)	Expected wt% C	wt% CO_3 (CHN)	wt% CO_3 (TGA)	CO_3 per Nb (CHN)	CO_3 per Nb (TGA)	wt % H_2O (TGA)	H_2O per Nb (TGA)	*X per Nb	formula ratios
$Nb_{16}Si_3$-CO_3	2.9	3.2	10.6	11.0	1.0	1.1	6.5	1.6	0.2	1 Nb : 1 CO_3 : 0.2 Si : 1.6 H_2O
$Nb_{16}Ge_3$-CO_3	2.8	3.1	10.1	10.4	0.99	0.98	7.5	1.7	0.2	1 Nb : 1 CO_3 : 0.2 Ge : 1.7 H_2O
SiO_2 wet gel	0.6		2.3	3.2	0.28	0.28	6.8	1.4	0.2	1 Nb : 0.3 CO_3 : 0.2 Si : 1.4 H_2O
SiO_2 dried gel	1.9		6.9	7.3	0.66	0.65	5.2	1.1	0.2	1 Nb : 0.7 CO_3 : 0.2 Si : 1.1 H_2O
GeO_2 wet gel	1.6		5.9	7.3	0.72	0.66	9.0	2.0	0.1	1 Nb : 0.7 CO_3 : 0.1 Ge : 1.8 H_2O
GeO_2 dried gel	1.8		6.5	7.0	0.61	0.61	6.2	1.3	0.1	1 Nb : 0.6 CO_3 : 0.1 Ge : 1.2 H_2O
PO_4 wet gel	1.5		5.5	6.6	0.78	0.60	8.8	2.0	0.2	1 Nb : 0.8 CO_3 : 0.2 P : 2 H_2O
PO_4 dried gel	2.1		7.7	8.1	0.93	0.71	6.6	1.4	0.2	1 Nb : 0.9 CO_3 : 0.2 P : 1.5 H_2O
Cs_4SiO_4 3hr CO_2 precip	1.5		5.5	12.9			20			

* *Where X = Si, Ge, or PO_4, which were calculated by the moles used in the original synthesis.*

13. Optical Spectroscopy

Table S25. Properties of the Nb-X thin-films as measured from spectroscopic ellipsometry and UV-vis spectroscopy.

Sample	Average roughness (nm)	Average thickness (nm)	Refractive index at 550 nm	*Average transmittance from 365 - 900 nm
Nb-Si	9.5	1497	1.566	89.0
Nb-Ge	1.3	805.8	1.598	90.4
Nb-PO_4	5.6	1084	1.568	90.1

**An average was taken due to the oscillations seen in the spectra (typical of thin-film interferences).*

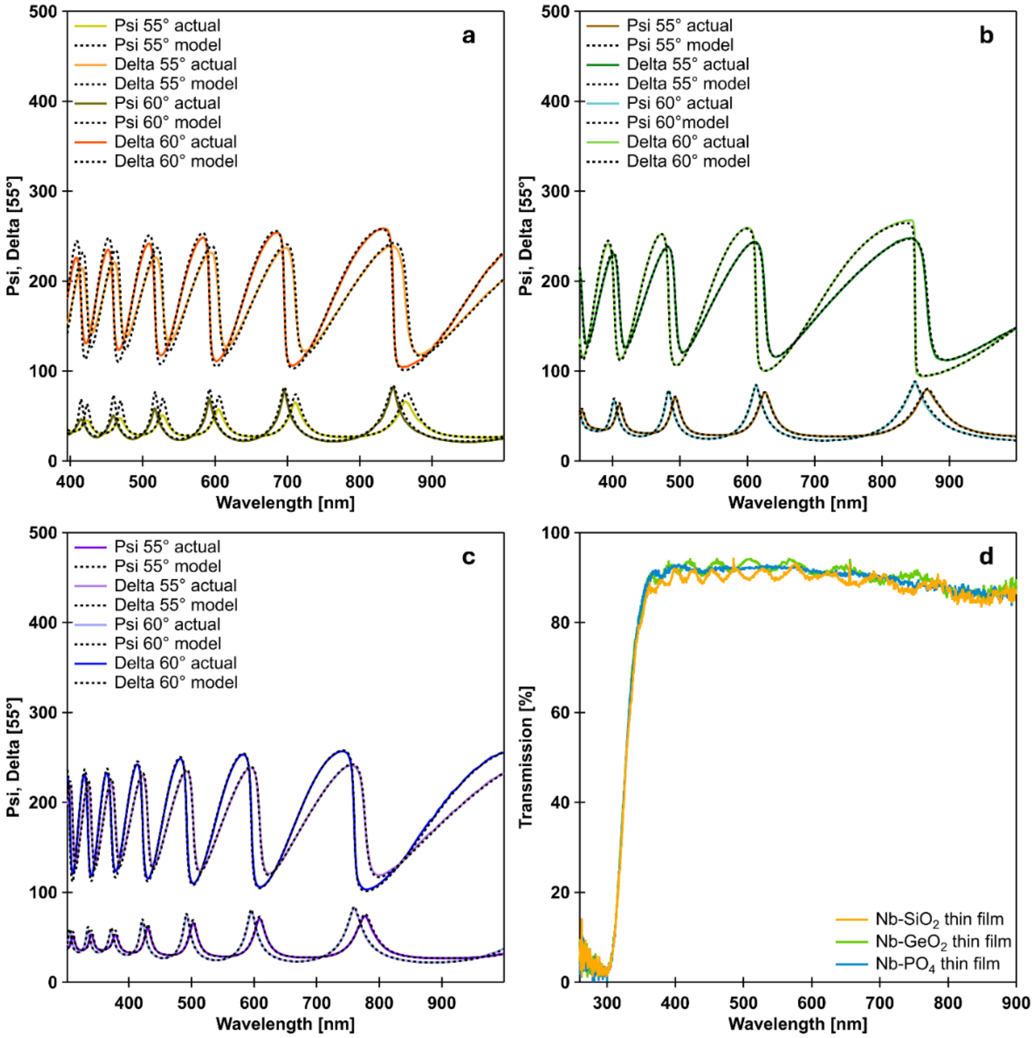

Figure S27. SE plots of (a) Nb-Si gel, (b) Nb-Ge gel, and (c) Nb-PO_4 gel, showing the fitting of psi and delta with the experimental data. (d) UV-vis spectra of the Nb-Si, Ge, and PO_4 gels as thin films on glass slides, highlighting their high optical transparency. Nb-Ge is the most transparent, followed by Nb-PO_4 and Nb-Si, which has the lowest transmittance.

14. Electronic Properties

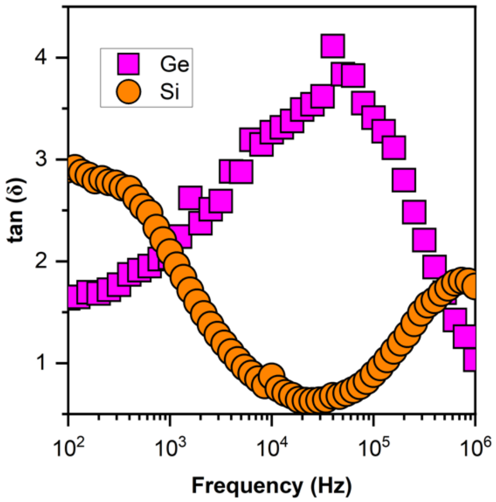

Figure S28. Dielectric loss tangent (tan(δ)) data as a function of frequency from 100 Hz to 1 MHz. The Si gels are shown as orange circles, and the Ge gels are shown as magenta squares.

Table S26. Impedance spectroscopy data for the Nb-Si and Nb-Ge gels, with resistance (R), resistivity (ρ), conductance and conductivity (σ), and capacitance (C), for ionic (i-subscript) and electronic (e-subscript) mechanisms.

	Nb-Si gel	Nb-Ge gel
R_i (kW)	180.4	117.3
r_i (W·cm)	1.21E+06	2.58E+05
σ_i (S)	5.50E-06	8.50E-06
s_i (conductivity, S·cm^{-1})	8.28E-07	3.88E-06
C_i (nF)	1.101	0.695
R_e (kW)	2.66E+16	2.27E+04
r_e (W·m)	1.78E+20	4.99E+07
σ_e (S)	3.8E-19	4.40E-08
s_e (conductivity, S·cm^{-1})	5.61E-21	2.01E-08
C_e (nF)	184.4	11.7

Supplementary Information References:

1 S. G. M, *SADABS*.
2 G. M. Sheldrick, *Acta Crystallogr. Sect. C Struct. Chem.*, 2015, **71**, 3–8.
3 J. Ilavsky and P. R. Jemian, *J. Appl. Crystallogr.*, 2009, **42**, 347–353.
4 R. K. Harris, E. D. Becker, S. M. Cabral De Menezes, R. Goodfellow and P. Granger, *Magn. Reson. Chem.*, 2002, **40**, 489–505.
5 M. Nyman, T. M. Alam, F. Bonhomme, M. A. Rodriguez, C. S. Frazer and M. E. Welk, *J. Clust. Sci.*, 2006, **17**, 197–219.
6 C. Hoch and C. Röhr, *Z. Für Naturforschung B*, 2001, **56**, 423–430.
7 C. Hoch and C. Röhr, *Z. Für Naturforschung B*, 2001, **56**, 1245–1256.
8 Y. Smolin I., Y. Shepelev F., R. Pomes, D. Hoebbel and W. Wieker, *Sov Phys Crystallogr Engl Transl*, 1979, **24**, 19–23.
9 M. Wiebcke, M. Grube, H. Koller, G. Engelhardt and F. Jurgen, *Microporous Mater.*, 1993, **2**, 55–63.
10 J. D. Kubicki and D. Sykes, *Phys. Chem. Miner.*, 1993, **19**, 381–391.
11 S. Hoffmann and T. F. Fässler, *Inorg. Chem.*, 2006, **45**, 7968–7972.
12 J. Hanuza, M. Ptak, M. Mączka, K. Hermanowicz, J. Lorenc and A. A. Kaminskii, *J. Solid State Chem.*, 2012, **191**, 90–101.
13 F. D. Hardcastle and I. E. Wachs, *Solid State Ion.*, 1991, **45**, 201–213.
14 M. Tillard, P. Hermet, A. Haidoux, D. Granier and P. Armand, *J. Solid State Chem.*, 2021, **295**, 121925.
15 J. Alvarado-Rivera, D. A. Rodríguez-Carvajal, M. del C. Acosta-Enríquez, M. B. Manzanares-Martínez, E. Álvarez, R. Lozada-Morales, G. C. Díaz, A. de Leon and M. E. Zayas, *J. Am. Ceram. Soc.*, 2014, **97**, 3494–3500.
16 P. N. de Aza, C. Santos, A. Pazo, S. de Aza, R. Cuscó and L. Artús, *Chem. Mater.*, 1997, **9**, 912–915.
17 W. Jastrzębski, M. Sitarz, M. Rokita and K. Bułat, *Spectrochim. Acta. A. Mol. Biomol. Spectrosc.*, 2011, **79**, 722–727.
18 P. Goj, B. Handke and P. Stoch, *Sci. Rep.*, 2022, **12**, 17495.
19 A. Lanir and N. T. Yu, *J. Biol. Chem.*, 1979, **254**, 5882–5887.
20 J.-H. Son and W. H. Casey, *Chem. Commun.*, 2015, **51**, 1436–1438.
21 M. H. Brooker and J. Wang, *Spectrochim. Acta Part Mol. Spectrosc.*, 1992, **48**, 999–1008.
22 X.-H. Guan, Q. Liu, G.-H. Chen and C. Shang, *J. Colloid Interface Sci.*, 2005, **289**, 319–327.
23 T. R. Briere and A. H. Sommer, *J. Appl. Phys.*, 1977, **48**, 3547–3550.
